# Comparative Transcriptional Profiling of 3 Murine Models of SLE Nephritis Reveals Both Unique and Shared Regulatory Networks

**DOI:** 10.1371/journal.pone.0077489

**Published:** 2013-10-22

**Authors:** Ramalingam Bethunaickan, Celine C. Berthier, Weijia Zhang, Matthias Kretzler, Anne Davidson

**Affiliations:** 1 Center for Autoimmunity and Musculoskeletal Diseases, Feinstein Institute for Medical Research, Manhasset, New York, New York, United States of America; 2 Department of Internal Medicine, Nephrology, University of Michigan, Ann Arbor, Michigan, United States of America; 3 Department of Medicine, Mount Sinai Medical Center, New York, New York, United States of America; INSERM-Université Paris-Sud, France

## Abstract

**Objective:**

To define shared and unique features of SLE nephritis in mouse models of proliferative and glomerulosclerotic renal disease.

**Methods:**

Perfused kidneys from NZB/W F1, NZW/BXSB and NZM2410 mice were harvested before and after nephritis onset. Affymetrix based gene expression profiles of kidney RNA were analyzed using Genomatix Pathway Systems and Ingenuity Pathway Analysis software. Gene expression patterns were confirmed using real-time PCR.

**Results:**

955, 1168 and 755 genes were regulated in the kidneys of nephritic NZB/W F1, NZM2410 and NZW/BXSB mice respectively. 263 genes were regulated concordantly in all three strains reflecting immune cell infiltration, endothelial cell activation, complement activation, cytokine signaling, tissue remodeling and hypoxia. STAT3 was the top associated transcription factor, having a binding site in the gene promoter of 60/263 regulated genes. The two strains with proliferative nephritis shared a macrophage/DC infiltration and activation signature. NZB/W and NZM2410 mice shared a mitochondrial dysfunction signature. Dominant T cell and plasma cell signatures in NZB/W mice reflected lymphoid aggregates; this was the only strain with regulatory T cell infiltrates. NZW/BXSB mice manifested tubular regeneration and NZM2410 mice had the most metabolic stress and manifested loss of nephrin, indicating podocyte loss.

**Conclusions:**

These findings identify shared inflammatory mechanisms of SLE nephritis that can be therapeutically targeted. Nevertheless, the heterogeneity of effector mechanisms suggests that individualized therapy might need to be based on biopsy findings. Some common mechanisms are shared with non-immune–mediated renal diseases, suggesting that strategies to prevent tissue hypoxia and remodeling may be useful in SLE nephritis.

## Introduction

Lupus nephritis is a devastating complication of SLE for which current treatment is insufficiently effective and excessively toxic. Histologic analysis of renal biopsies does not always predict outcome or response to therapy. Furthermore, the study of pathogenic mechanisms of SLE nephritis is hampered by the small amount of biopsy material, the invasiveness of repeat biopsies and by therapeutic interventions begun prior to biopsy. It therefore remains essential to study animal models [1] in which the whole organ can be studied without the confounding effects of medications.

In these studies, we used transcriptional profiling to define similarities and differences in the renal inflammatory process between three well-characterized models of SLE nephritis. Female NZB/W F1 mice develop high titers of IgG2a anti-dsDNA autoantibodies and proliferative glomerulonephritis similar to class IV human lupus nephritis [1], [2]. NZM2410 mice, while genetically similar to NZB/W, express high levels of IL-4 and IgG1 and IgE autoantibodies; they develop rapidly progressive glomerulosclerosis with scant lymphocytic infiltrate [3]. Male NZW/BXSB mice carry the *Yaa* (Y linked autoimmune acceleration) locus containing a reduplication of the *Tlr7* gene [4]. They develop anti-RNA and anti-cardiolipin antibodies and proliferative glomerulonephritis with severe tubulointerstitial inflammation [5]. Not surprisingly, differences in both pathogenic mechanisms and responses to immunologic interventions have been observed in the three models [6], [7]. These differences parallel the emerging appreciation of heterogeneity in human SLE nephritis [8], [9].

Our first goal was to identify shared expression profiles between the three models during active untreated nephritis; these define major driving forces in disease pathogenesis and may help in the development of broadly applicable treatment strategies. We next wished to identify profiles associated with proliferative nephritis, a subtype with a poor prognosis. Our final goal was to determine whether different strains with similar or different histologic lesions have unique features that could guide the choice of lupus model to study specific features of disease.

While a core set of regulated genes was shared among the three models, there were many differences, even between the two genetically similar strains and between the two strains with proliferative disease. Unique gene expression profiles accounted for approximately one third of the expression profile in each strain and revealed individual features of potential pathogenic significance. Our findings reflect the heterogeneity of renal responses to immune complex deposition and inflammation and suggest that targeting the appropriate effector mechanism in individual patients may improve the treatment of SLE nephritis.

## Materials and Methods

### Ethics statement

This study was carried out in strict accordance with the recommendations in the Guide for the Care and Use of Laboratory Animals of the National Institutes of Health. The protocol was approved by the Institutional Animal Care and Use Committee (IACUC) of the Feinstein Institute (Protocol Number: 2007-054). All surgery was performed under ketamine/xylazine anesthesia, and all efforts were made to minimize suffering.

### Mouse models

Kidneys were harvested after cardiac perfusion with 60 ml of sterile saline. A detailed description of the samples used for microarray analysis and derivation of the gene sets of interest has been previously published [10].

**NZBW:** NZB/NZW F1 female kidneys were harvested at the age of 6 (n = 7) or 16 (n = 8) weeks (no serum autoantibodies, immune complex deposition, or proteinuria) and 36–40 weeks (established proteinuria >300 mg/dl for >2 wk, n = 10) [11].

**NZW/BXSB:** Male (NZW×BXSB) F1 kidneys were obtained at the age of 8 weeks (no serum autoantibodies or proteinuria n = 4), 17 weeks (autoantibodies but no proteinuria, n = 6) and 18–21 weeks (established proteinuria >300 mg/dl for >2 wk and histologic glomerular score >2, n = 12; 6 randomly used for microarray).

**NZM2410:** NZM2410 kidneys were obtained at 6–8 weeks (no autoantibodies, renal immune complex deposition or proteinuria, n = 5) and 22–30 weeks (proteinuria >300 mg/dL for 7–10 days, n = 7; 5 randomly used for microarray). NZM2410 mice were harvested early after proteinuria onset as most die within 14 days [12].

### Histologic assessment of kidneys

H & E stained sections were scored for glomerular and interstitial damage by a single blinded observer using a semi-quantitative scale from 0–4 as previously described [11].

Paraffin embedded sections were dewaxed and deparaffinized. Endogenous peroxidase activity was blocked with 3%H_2_O_2_ and antigen retrieval performed in 10 mM citrate buffer pH6. Slides were stained with at anti-Ki67 (Dako, Carpinteria, CA) or PCNA (Invitrogen Life Technologies, Grand Island, NY) followed by biotin conjugated anti-rat Ig and ABC developer (Vectastain, Burlingame, CA).

### RNA purification and microarray hybridization

RNA extraction, cDNA synthesis, hybridization, microarray processing, data normalization and filtering were performed as previously described [10], [13]. Microarray gene expression data were normalized and batch-corrected together, and quality controls were used before further analyses. Significantly regulated genes were analyzed by creating biological literature-based networks using Genomatix Pathway System software (GePS) (www.genomatix.de). Canonical pathways were analyzed using Ingenuity Pathway Analysis software (IPA) (www.ingenuity.com). Principal component analysis was performed using ArrayTrack™ software (http://www.fda.gov/ArrayTrack). Gene expression datasets are on Gene Expression Omnibus (GEO) at http://www.ncbi.nlm.nih.gov/geo/ (accession numbers GSE32583, GSE44691 and #GSE49898).

### Quantitative Real Time PCR (qPCR)

We selected to validate by qRT-PCR 158 genes including some that were highly significant, some that had a lower fold change and several genes involved in pathways of interest such as mitochondrial function, ER stress and control of circadian rhythm. A number of cytokine genes that are not expressed in normal kidneys and did not pass the cut-off on the arrays, were also tested by qRT-PCR.

Validation of selected genes was performed by qPCR as previously described (LightCycler480, Roche Diagnostics - [11], [13]; primers on request). Data were analyzed using a comparative cycle threshold (2-ΔΔCT) method, normalized to β-actin and expressed as fold induction relative to a pre-nephritic calibrator of the same strain [11], [13].

### Statistical Analysis

The TIGR MultiExperiment Viewer (TMEV) application [14 #5174] was used for statistical analysis of microarray and qPCR data. For the array study, statistical unpaired analyses for each comparison were performed using the Significance Analysis of Microarrays (SAM) method and the following criteria: q-value ≤0.001 and fold change ≥1.4 for the upregulated genes and ≤0.7 for the downregulated genes. We used more stringent filter criteria than in our mouse-human comparison [10] so as to capture the maximum number of genes with differential expression. To avoid ambiguity between species, mouse genes were converted to the corresponding human orthologs using the NCBI homolog (Build 64) and Genomatix annotated ortholog databases (**Table S1**).

For the qPCR data, unpaired SAM and t-test were performed. Unsupervised hierarchical clustering with bootstrap procedures was performed using Euclidean metrics with average or complete linkage and visualized using TMEV. Data were scaled to the mean of the young mice in each strain, given a value of 1. Genes regulated between two groups with a fold change of >2 and q-value <0.05 were considered significant.

### BrDU staining of renal cells

Groups of 3-6 NZB/W and NZW/BXSB mice were fed BrDU as previously described [13] for 12–15 days. Kidneys were perfused and harvested as above and flow cytometry was performed as previously described [13].

## Results

**Figure 1:**
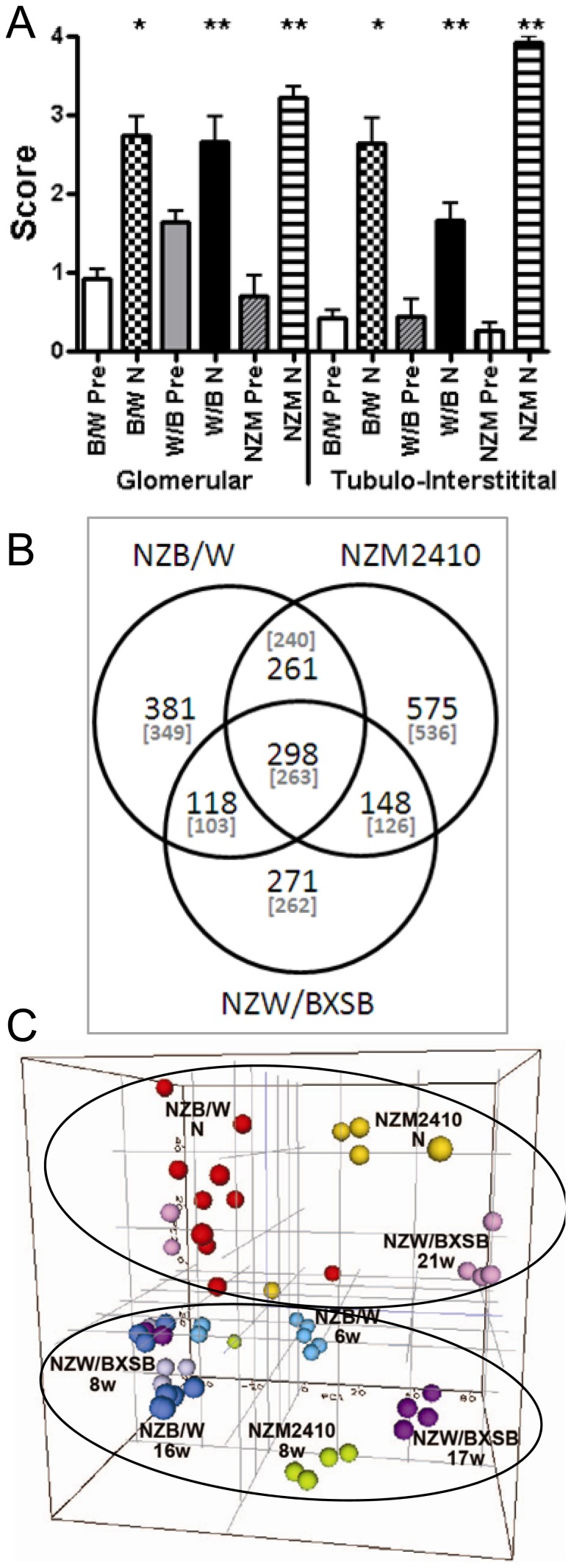
A. Glomerular and interstitial damage scores in pre-nephritic (Pre) and nephritic (N) mice (mean + SD) of NZB/W (B/W), NZW/BXSB (W/B) and NZM2410 NZM) strains (* p<0.001; ** p<0.01). The high tubulointerstitial score in the NZM2410 strain reflects severe tubular atrophy. B. Shared and unique gene expression profiles of each of the three strains. Parentheses indicate the number of genes with a human ortholog. C. 3D principal component analysis from the 14780 genes passing the cutoff value (see Materials and Methods) after normalization and batch correction of the arrays from the 3 mouse strains together.

The clinical characteristics and histologic renal appearance of the three strains have previously been reported [11], [12], [15], [16]; renal damage scores are displayed in **Figure 1A**. Differentially regulated genes were identified by comparing the renal profiles of the nephritic mice to their prenephritic control groups. 955 (442 down, 513 up), 755 (208 down, 547 up) and 1168 (628 down, 540 up) genes were differentially regulated in NZB/W, NZW/BXSB and NZM2410 models respectively (**Figure 1B** and **Table S1**). Principal component analysis of all genes expressed in the 3 strains showed a clear separation of the nephritic vs. non-nephritic mice as well as differences between strains (**Figure 1C**).

### Profiles shared by all three strains

**Figure 2:**
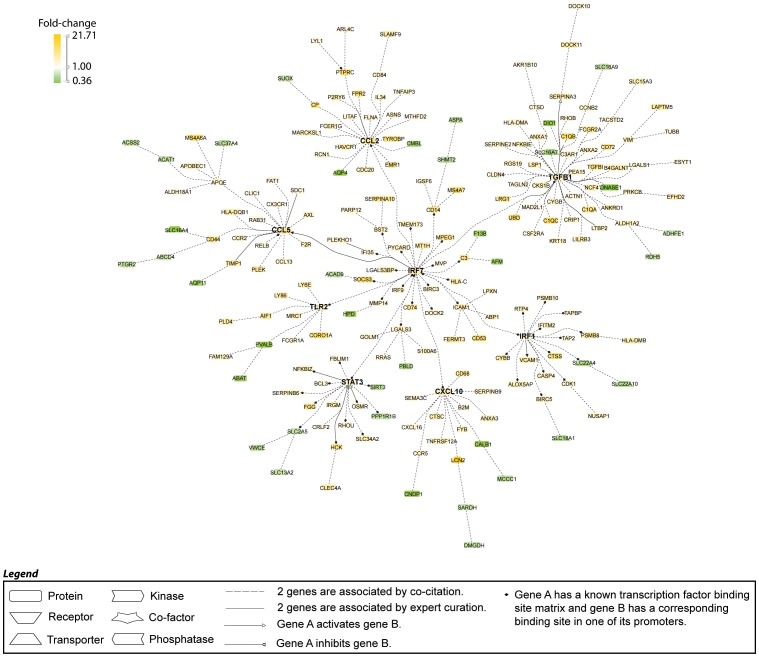
Literature-based analysis of genes shared among all three strains using Genomatix Pathway System (GePS) software. 263 human gene orthologs were regulated in the same direction in the nephritic vs. prenephritic kidneys in NZB/W, NZM2410 and NZW/BXSB. The picture shows the 204 genes that were co-cited in PubMed abstracts in the same sentence. Orange represents the genes that are upregulated and green represents the genes that are downregulated in nephritic compared to prenephritic mice.

263 genes (67 down and 196 up) were regulated in the same direction in all 3 strains. Transcriptomic network analysis using Genomatix Pathway System was performed to identify the major nodes along with their gene clusters (**Figure 2** and data not shown). These nodes reflect transcripts associated with macrophage activation (CD68, activating Fc receptors, FPR2, C type lectins, cathepsins, HLA-DM), classical complement pathway proteins, endothelial activation (VCAM-1, ICAM-1), metabolic stress and proteasome activity (HPD, CNDP1, UBD), tissue remodeling and fibrosis (TIMP-1, MMP14, TGFB1) and tubular damage (HAVCR1, LCN2). Three cytokines, IL1f6 (IL-36), IL-34 and TGFβ, were shared by all three strains as well as a limited group of chemokines and chemokine receptors, including CCL2, CCL5, CCL9 and their receptors CCR2 and CCR5 as well as CXCL10 and CXCL16. Other genes of interest include the TWEAK receptor FN14, whose expression on intrinsic renal cells promotes glomerulonephritis [17], ANKRD1, which is associated with proteinuria in SLE nephritis [18], TLRs 2 and 13, and several proteinase inhibitors of the Serpin family involved in the coagulation pathway. SMPDL3B, the renal target of Rituximab [19] was also upregulated in all three strains. Transcription factor analysis identified STAT3 as the top transcription factor having binding sites in the promoter regions of 60 of the 263 genes (**Table S2**), followed by transcription factors involved in interferon signaling (IRF1 and IRF7) and RELB (**Table 1** **,** **Figure 2**). Accordingly, we identified 48, 34 and 32 of a panel of 195 interferon inducible genes [20], [21], [22] in NZB/W, NZM2410 and NZW/BXSB strains respectively (not shown). Consistent with these findings, the top ingenuity pathways included antigen processing and presentation, dendritic cell maturation, complement activation, and pathways reflecting innate and adaptive immune activation (**Table 1**).

**Table 1:** Top 10 canonical pathways significantly regulated sorted by Benjamini-Hochberg Multiple Testing corrected p-value (p-value<0.05), as assessed by IPA (Ingenuity Pathway Analysis).

Canonical pathways(number of genes regulated in the pathway/number of genes in the pathway)	B-H Multiple testing corrected p-value	Regulated molecules in the pathway
**From the 3 mouse model overlap (263 genes)** * *STAT3, IRF1, * ***RELB*** *, * ***IRF7*** *, IRF9* †		
Antigen Presentation Pathway (8/40)	9.70E-06	B2M, HLA-DMA, HLA-C, HLA-DMB, **PSMB8**, CD74, TAP2, TAPBP
Dendritic Cell Maturation (14/207)	1.90E-04	B2M, HLA-DMA, **ICAM1**, FCGR2A, TYROBP, NFKBIE, **RELB**, **IL36A**, HLA-DMB, HLA-DQB1, FCGR1A, TLR2, HLA-C, FCER1G
OX40 Signaling Pathway (7/94)	4.09E-03	B2M, HLA-DMA, HLA-C, NFKBIE, FCER1G, HLA-DMB, HLA-DQB1
Role of Pattern Recognition Receptors in Recognition of Bacteria and Viruses (9/106)	4.09E-03	TLR2, **IRF7**, **C3**, C1QC, **C1QA**, C1QB, **CCL5**, C3AR1, PRKCB
Graft-versus-Host Disease Signaling (6/50)	4.09E-03	HLA-DMA, HLA-C, FCER1G, **IL36A** , HLA-DMB, HLA-DQB1
Complement System (5/35)	5.22E-03	**C3**, C1QC, **C1QA**, C1QB, C3AR1
Altered T Cell and B Cell Signaling in Rheumatoid Arthritis (8/92)	5.22E-03	TLR2, HLA-DMA, **TGFB1**, **RELB**, FCER1G, **IL36A**, HLA-DMB, HLA-DQB1
Allograft Rejection Signaling (6/95)	5.73E-03	B2M, HLA-DMA, HLA-C, FCER1G, HLA-DMB, HLA-DQB1
Role of Hypercytokinemia / hyperchemokinemia in the Pathogenesis of Influenza (5/44)	5.73E-03	CXCL10, **CCR5**, **CCL2**, **IL36A** , **CCL5**
Cytotoxic T Lymphocyte-mediated Apoptosis of Target Cells (6/85)	7.40E-03	B2M, HLA-DMA, HLA-C, FCER1G, HLA-DMB, HLA-DQB1
**From the NZB/W only (349 genes)** * *CEBPB, IRF4, FLI1, IRF8, IKZF1* †		
CTLA4 Signaling in Cytotoxic T Lymphocytes (14/98)	3.06E-06	FYN, PTPN6, PIK3R5, PIK3C2G, CD3D, CD28, LCK, SYK, LAT, PPM1L, CD86, HLA-DOB, PTPN22, LCP2
CD28 Signaling in T Helper Cells (13/132)	3.47E-04	FYN, PTPN6, PIK3R5, PIK3C2G, CD3D, CD28, LCK, SYK, LAT, CD86, HLA-DOB, VAV1, LCP2
PKCθ Signaling in T Lymphocytes (11/143)	5.25E-03	FYN, CD28, LCK, LAT, PIK3R5, PIK3C2G, CD86, HLA-DOB, VAV1, CD3D, LCP2
Natural Killer Cell Signaling (10/116)	5.25E-03	FYN, LCK, PTPN6, SYK, LAT, PIK3R5, PIK3C2G, SH3BP2, VAV1, LCP2
T Cell Receptor Signaling (10/109)	5.25E-03	BTK, FYN, CD28, LCK, LAT, PIK3R5, PIK3C2G, VAV1, CD3D, LCP2
Role of NFAT in Regulation of the Immune Response (13/198)	6.23E-03	FYN, CD79B, PIK3R5, PIK3C2G, CD3D, BTK, CD28, LCK, SYK, LAT, CD86, HLA-DOB, LCP2
iCOS-iCOSL Signaling in T Helper Cells (10/123)	6.41E-03	CD28, LCK, IL2RG, LAT, PIK3R5, PIK3C2G, HLA-DOB, VAV1, CD3D, LCP2
B Cell Development (5/33)	9.83E-03	IL7R, SPN, CD79B, CD86, HLA-DOB
Crosstalk between Dendritic Cells and Natural Killer Cells (8/95)	9.83E-03	CSF2RB, TLR4, CD28, IL2RG, LTB, CD86, CD83, HLA-F
Primary Immunodeficiency Signaling (6/62)	9.83E-03	IL7R, BTK, LCK, IL2RG, ADA, CD3D
**From the NZM2410 only (536 genes)** * ***JUN*** *, NFKB1, MYC, EGR1, WT1* †		
Putrescine Degradation III (5/30)	2.64E-02	ALDH1B1, ALDH1A1, SAT2, SMOX, ALDH9A1
Aryl Hydrocarbon Receptor Signaling (14/161)	2.64E-02	ALDH1B1, NFKB2, NFKB1, CCND1, ALDH9A1, MYC, GSTT1, GSTM2, ALDH1A1, **JUN**, CDKN1A, DHFR, GSTO2, NFE2L2
LPS/IL-1 Mediated Inhibition of RXR Function (18/239)	2.64E-02	ECSIT, ALDH1B1, NR1H4, IL1R1, ALDH9A1, **SOD3**, CHST15, GSTT1, GSTM2, ALDH1A1, **JUN**, IL1RN, ACSL5, ALAS1, HS6ST2, FABP1, SMOX, GSTO2
TCA Cycle II (Eukaryotic) (5/41)	7.08E-02	IDH3G, ACO2, DLST, SDHC, ACO1
Guanine and Guanosine Salvage I (2/9)	8.55E-02	PNP, HPRT1
Cysteine Biosynthesis/Homocysteine Degradation (2/8)	8.55E-02	CBS, CTH
Role of IL-17F in Allergic Inflammatory Airway Diseases (6/47)	1.03E-01	IGF1, CCL7, CREB3, IL17RC, NFKB2, NFKB1
Tryptophan Degradation X (Mammalian, via Tryptamine) (4/29)	1.03E-01	ALDH1B1, ALDH1A1, SMOX, ALDH9A1
Valine Degradation I (4/35)	1.45E-01	HIBADH, BCKDHA, ACAD8, ACADSB
Dopamine Degradation (4/37)	1.45E-01	ALDH1B1, ALDH1A1, SMOX, ALDH9A1
**From the NZW/BXSB only (262)** * *LHX1* †		
Cell Cycle Control of Chromosomal Replication (6/31)	1.50E-03	MCM3, MCM6, MCM2, CDT1, DBF4, MCM7
Hepatic Fibrosis / Hepatic Stellate Cell Activation (10/146)	2.24E-02	MYL9, COL1A1, LY96, MYH14, ACTA2, IGFBP3, **IL6** , PDGFB, PDGFRB, COL3A1
Atherosclerosis Signaling (8/136)	6.32E-02	COL1A1, MSR1, PLA2G5, CD36, SERPINA1, **IL6** , PDGFB, COL3A1
Complement System (4/35)	6.32E-02	C5AR1, C8A, C2, C8G
DNA Double-Strand Break Repair by Homologous Recombination (3/17)	9.33E-02	LIG1, POLA1, BRCA1
LXR/RXR Activation (7/136)	1.57E-01	LY96, MSR1, APOH, CD36, SERPINA1, **IL6** , GC
Role of BRCA1 in DNA Damage Response (5/65)	1.57E-01	RFC4, RBL1, BRCA1, CHEK1, RFC3
Intrinsic Prothrombin Activation Pathway (3/35)	3.93E-01	COL1A1, F13A1, COL3A1
Role of CHK Proteins in Cell Cycle Checkpoint Control (4/57)	4.36E-01	RFC4, BRCA1, CHEK1, RFC3
Estrogen Biosynthesis (3/49)	4.96E-01	CYP2D6, CYP2F1, HSD17B2
**From the NZB/W with NZM2410 overlap (240 genes)** *		
Mitochondrial Dysfunction (10/174)	1.30E-02	SDHA, NDUFS5, SDHB, NDUFS1, **SOD2**, UQCRC2, LRRK2, NDUFB10, **COX15**, AIFM1
Valine Degradation I (4/35)	3.03E-02	ECHS1, AUH, ALDH6A1, BCKDHB
TCA Cycle II (Eukaryotic) (4/41)	3.81E-02	SDHA, SUCLA2, SDHB, IDH3B
Oleate Biosynthesis II (Animals) (3/18)	4.06E-02	FADS2, ALDH6A1, FADS1
Ethanol Degradation II (4/43)	4.50E-02	ALDH4A1, ACSL3, PECR, ALDH7A1
D-glucuronate Degradation I (2/13)	4.50E-02	CRYL1, DCXR
Fatty Acid β-oxidation I (4/45)	4.89E-02	ACSL3, ECHS1, AUH, HADH
Methylmalonyl Pathway (2/12)	4.89E-02	PCCA, MCEE
Arginine Degradation I (Arginase Pathway) (2/13)	4.89E-02	ALDH4A1, ARG2
Molybdenum Cofactor Biosynthesis (2/15)	4.89E-02	GPHN, NFS1
**From the NZM2410 with NZW/BXSB overlap (126 genes)** *		
Leucine Degradation I (2/26)	2.54E-01	IVD, ACADM
LXR/RXR Activation (5/136)	2.54E-01	TNFRSF1A, LPL, CLU, ABCA1, CYP51A1
Asparagine Degradation I (1/4)	3.65E-01	ASPG
Thiamin Salvage III (1/5)	3.65E-01	TPK1
Sertoli Cell-Sertoli Cell Junction Signaling (5/195)	4.68E-01	TUBB6, CLDN1, TNFRSF1A, CLDN16, CLDN7
Triacylglycerol Degradation (2/32)	4.68E-01	LPL, MGLL
Cell Cycle Control of Chromosomal Replication (2/31)	4.68E-01	CDC6, MCM4
Methionine Salvage II (Mammalian) (1/9)	4.68E-01	BHMT2
Fatty Acid β-oxidation I (2/45)	4.68E-01	IVD, ACADM
LPS/IL-1 Mediated Inhibition of RXR Function (5/239)	4.68E-01	ALDH1L2, ACOX2, TNFRSF1A, ACOX3, ABCA1
**From the NZW/BXSB with NZB/W overlap (103 genes)** * *SPI1* †		
Hepatic Fibrosis / Hepatic Stellate Cell Activation (7/146)	1.27E-02	**COL1A2**, FN1, IL10RA, MYH9, **EGF**, IFNGR1, **MMP2**
Caveolar-mediated Endocytosis Signaling (5/85)	1.49E-02	**ITGB2**, **ITGAM**, CD48, **EGF**, ITGAX
Atherosclerosis Signaling (5/136)	6.73E-02	IL33, **COL1A2**, **ITGB2**, F3, SELPLG
Inhibition of Matrix Metalloproteases (3/40)	6.73E-02	THBS2, **MMP2**, LRP1
IL-8 Signaling (6/205)	6.73E-02	**ITGB2**, **ITGAM**, **EGF**, IKBKE, **MMP2**, ITGAX
Leukocyte Extravasation Signaling (6/201)	6.73E-02	**ITGB2**, NCF1, **ITGAM**, THY1, **MMP2**, SELPLG
Role of Pattern Recognition Receptors in Recognition of Bacteria and Viruses (4/106)	1.29E-01	IFIH1, CLEC7A, TLR7, CLEC6A
Activation of IRF by Cytosolic Pattern Recognition Receptors (3/72)	1.31E-01	IFIH1, IKBKE, IFIT2
Extrinsic Prothrombin Activation Pathway (2/20)	1.31E-01	F5, F3
Colorectal Cancer Metastasis Signaling (6/258)	1.31E-01	TLR7, **EGF**, IFNGR1, **MMP2**, ADCY7, LRP1

Genes tested by RT-PCR are highlighted in bold and underlined.

* (number of genes regulated in the same direction).

† top transcription factors for each analysis as assessed by GePS.

### Profiles shared by two strains, based on the defined filter criteria

**Figure 3:**
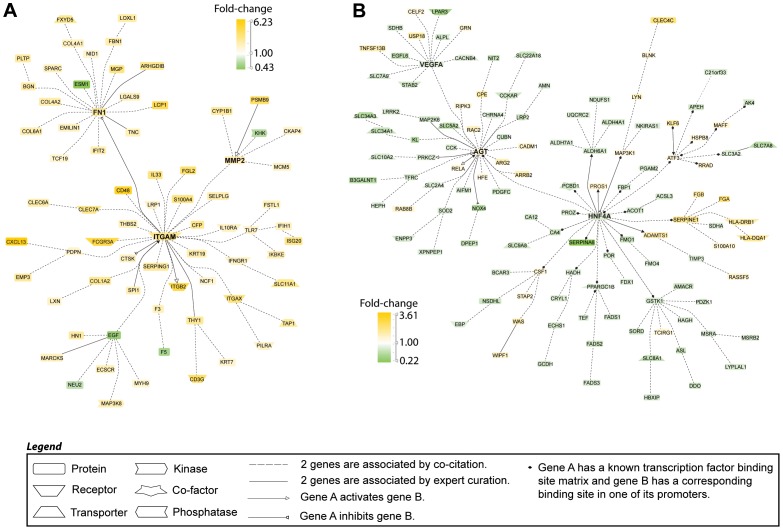
Literature-based analysis of limited gene expression patterns using Genomatix Pathway System (GePS) software. **A**. 103 shared genes were regulated in the same direction in the nephritic vs. prenephritic kidneys in NZW/BXSB and NZB/W mouse models. The picture shows the 72 that were co-cited in the same sentence of PubMed abstracts. **B**. 240 genes were regulated in the same direction in the nephritic vs. prenephritic kidneys in NZB/W and NZM2410 mouse models. The picture shows the 124 genes that were co-cited in PubMed abstracts in the same sentence. Orange represents the genes that are upregulated and green represents the genes that are downregulated in nephritic compared to prenephritic mice.

As described in the methods section, we applied stringent filter criteria to capture the maximum number of genes with differential expression, allowing us to identify the most important LN related genes in the development of the disease in the different mouse models. This filter does however exclude those genes with a trend towards differences in expression. **Table 2** shows the regulation and associated significance in each mouse model of selected genes of interest. 103 genes were preferentially regulated (16 down and 87 up) in the NZB/W and NZW/BXSB strains with proliferative glomerulonephritis. These included the two chains of the CD11b molecule as well as other macrophage/DC expressed genes (IKBKE, FCGR3A, CXCL13) [11], [15]. These strains also had increased collagen type IV expression indicating expansion of basement membrane, as well as of collagen type 1, fibronectin, thrombospondin-2 and MMP2, indicating interstitial fibrosis and remodeling (**Figure 3A**). Transcription factor analysis revealed SPI-1 (PU.1) as the top transcription factor (**Table 1**), consistent with its role in inflammatory macrophage function and antigen presentation [23].

**Table 2:** Expression and significance of selected genes.

	NZB/W		NZM2410		NZW/BXSB	
Gene name	Fold-change	q-value	Fold-change	q-value	Fold-change	q-value
*Genes shared by two strains, based on the defined filter criteria*						
IKBKE	**1.72**	**0.0000**	1.638	0.0012	**1.43**	**0.0000**
FCGR3A	**3.87**	**0.0000**	1.654	0.0015	**2.06**	**0.0005**
CXCL13	**6.22**	**0.0000**	1.781	0.0079	**2.50**	**0.0000**
COL4A1	**1.59**	**0.0000**	*1.131*	*0.1691*	**1.76**	**0.0000**
FN1	**1.99**	**0.0000**	1.614	0.0029	**1.94**	**0.0000**
THBS2	**1.62**	**0.0006**	1.726	0.0112	**1.59**	**0.0000**
MMP2	**1.66**	**0.0000**	1.423	0.0101	**1.87**	**0.0000**
PROS1	**1.64**	**0.0000**	**1.53**	**0.0006**	1.34	0.0005
FGA	**3.61**	**0.0000**	**4.53**	**0.0005**	*1.44*	*0.2188*
FGB	**2.57**	**0.0004**	**4.82**	**0.0005**	1.83	0.0273
SERPINE1	**2.93**	**0.0000**	**5.83**	**0.0005**	1.72	0.0043
SERPINA6	**0.22**	**0.0000**	**0.04**	**0.0000**	*np*	
KRT20	2.37	0.0030	**17.06**	**0.0000**	**2.18**	**0.0000**
CLDN1	1.45	0.1531	**2.89**	**0.0000**	**1.79**	**0.0000**
CLDN7	1.56	0.0039	**2.71**	**0.0004**	**1.55**	**0.0000**
CLU	1.62	0.0048	**3.04**	**0.0000**	**1.96**	**0.0000**
CXCL1	1.84	0.0341	**5.47**	**0.0000**	**2.35**	**0.0000**
CXCL2	1.67	0.0250	**4.77**	**0.0000**	**2.93**	**0.0000**
*Genes unique to one model, based on the defined filter criteria*						
CD3D	**2.28**	**0.0000**	1.61	0.0057	1.31	0.0023
PTPN22	**2.60**	**0.0000**	2.03	0.0139	*0.86*	*0.2124*
FYN	**1.48**	**0.0000**	*1.09*	*0.3296*	*1.09*	*0.1870*
LCP2	**1.42**	**0.0000**	1.26	0.0062	1.24	0.0058
HLA-DOB	**1.78**	**0.0000**	*np*		*np*	
LCK	**1.73**	**0.0000**	1.43	0.0042	1.30	0.0027
SYK	**1.70**	**0.0000**	*1.15*	*0.1163*	1.29	0.0004
HELLS	1.67	0.0011	1.80	0.0016	**3.38**	**0.0000**
MCM2	1.33	0.0024	1.24	0.0212	**1.67**	**0.0002**
MCM3	1.39	0.0004	1.22	0.0315	**1.78**	**0.0000**
MCM6	*1.30*	*0.1408*	1.39	0.0084	**2.01**	**0.0000**
MCM7	1.33	0.0000	1.37	0.0000	**1.47**	**0.0003**
AURKB	*Np*		*np*		**1.81**	**0.0000**
COL5A1	1.43	0.0250	*1.03*	*0.4772*	**2.04**	**0.0000**
COL5A2	*1.02*	*0.9999*	0.83	0.0397	**1.68**	**0.0000**
COL6A2	1.41	0.0097	*1.12*	*0.4009*	**1.85**	**0.0000**
COL6A3	*1.15*	*0.4089*	*1.05*	*0.9999*	**2.03**	**0.0003**
NFKB1	1.27	0.0017	**1.43**	**0.0000**	1.34	0.0000
MYC	1.72	0.0015	**2.50**	**0.0006**	1.50	0.0013
EGR1	*0.85*	*0.9999*	**3.16**	**0.0006**	*0.63*	*0.2291*
JUN	*1.10*	*0.4309*	**2.17**	**0.0000**	*0.85*	*0.2382*
SOD3	0.80	0.0250	**0.59**	**0.0005**	*0.94*	*0.1747*

The genes passing the defined filter criteria are highlighted in bold. np: genes not passing the Affymetrix negative controls cut-off. In italic are the genes not significantly regulated (q-value>0.05).

NZM2410 mice shared 240 genes with the NZB/W strain (**Figure 3B**) and 126 genes with NZW/BXSB. NZB/W and NZM2410 mice shared genes associated with the coagulation and fibrinolytic cascades (Protein S, fibrinogen and plasminogen activator inhibitor - SERPINE) with HNF4A being the top transcription factor (**Tables 1** **and** **2**). Decreased expression of Serpina6 (corticosteroid binding protein) was also shared between these two strains (**Table 2**). The top shared ingenuity pathway was mitochondrial dysfunction **(** **Table 1** **)**. Upregulated genes shared between NZM2410 and NZW/BXSB included epithelial cell proteins (Keratin20, Claudin1, Claudin7, CLU) and the chemokines CXCL1 and CXCL2 that both bind to CXCR2 (**Table 2**).

### Unique gene profiles, based on the defined filter criteria

Each strain also expressed unique genes during nephritis, comprising 37, 35 and 46% of the total genes in the expression profiles of NZB/W, NZW/BXSB and NZM2410 kidneys respectively. In NZB/W mice, the major signal was derived from T cells, with 4 of the top 5 canonical pathways involving T cell activation (**Tables 1** **and** **2**). High levels of immunoglobulin transcripts in this strain reflected plasma cell infiltration. The top transcription factor was C/EBPβ a regulator of monocyte/macrophage proliferation and differentiation [24]. In NZW/BXSB mice there was evidence of chromosomal replication/chromatin remodeling (HELLS, MCM2-7, AURKB) suggesting cellular proliferation (**Table 2**). In addition, increased expression of collagen V and VI genes indicated a greater degree of interstitial fibrosis than in the other two strains (**Table 2**). The top transcription factor, LHX1, has essential roles in multiple steps of epithelial tubular morphogenesis during kidney organogenesis [25]. NZM2410 mice differentially regulated genes involved in multiple metabolic pathways, indicating cellular stress. Top transcription factors in this strain included JUN, a mediator of TGFβ induced fibrosis related to ER stress [26], NFKB, MYC and EGR1 (**Tables 1** **and** **2**).

### Validation by RT-PCR

**Figure 4:**
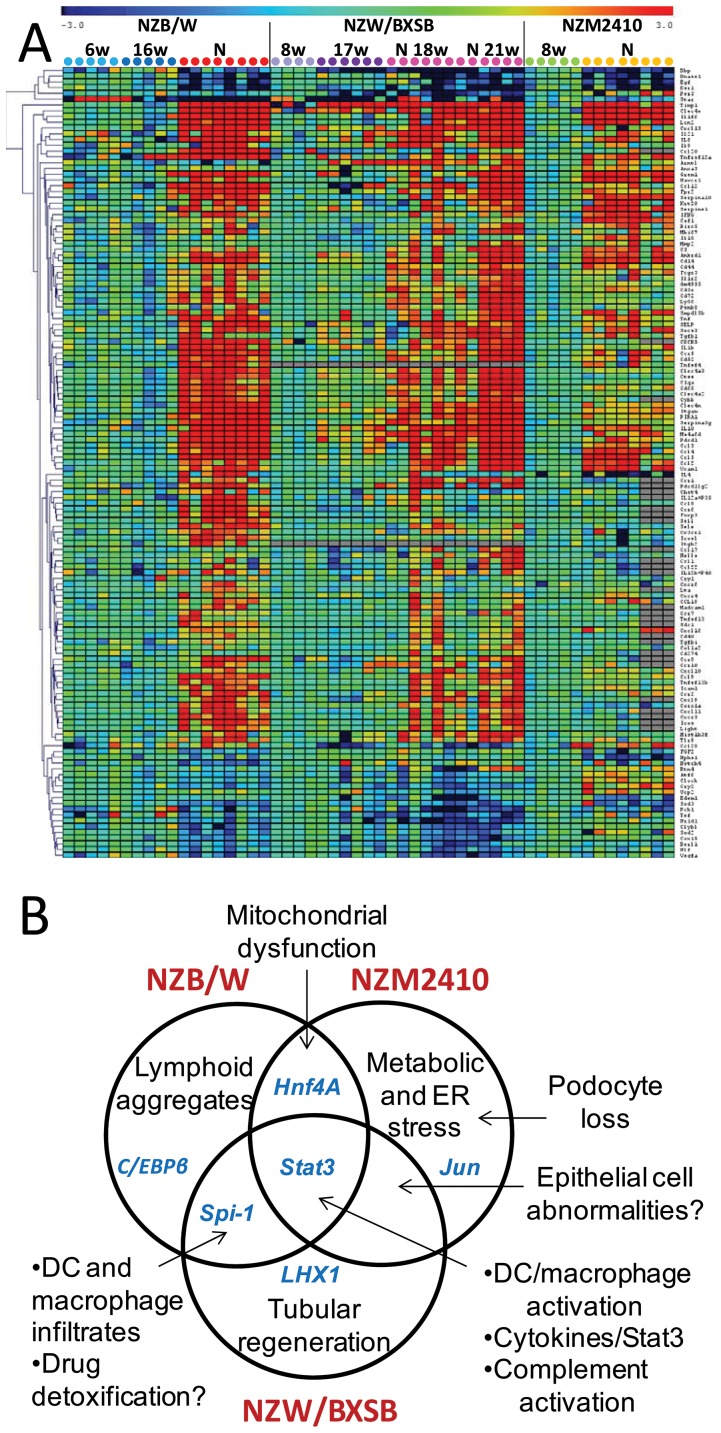
A. One way cluster analysis of genes with significantly altered expression in the PCR validation set (See Table S3). Gene expression was scaled to the mean of pre-nephritic controls for each strain. Significantly up or downregulated (>2 fold) genes by SAM with q value <0.05 are shown (corresponding to 137 genes). B. Summary of the unique and shared pathogenic pathways identified in the kidneys of the three mouse strains.

After excluding genes that were not expressed on the arrays, the concordance between the microarray data and qPCR data was 85.0%, 87.8% and 93.9% in the NZW/BXSB, NZM2410 and NZB/W strains respectively (**Figure 4A** **, Table S3**). An increased sensitivity of the PCR assays accounted for almost all the observed discordance in NZB/W and NZW/BXSB mice whereas more than half of the discordance in NZM2410 mice was in genes that were <2 fold regulated on the microarrays. The higher discordance rates between microarray and real time PCR when low stringency cut-offs (<2 fold differences) are applied has previously been reported [27] but a higher stringency results in decreased specificity.

All three strains manifested significant regulation of 42 genes including LCN2, C3, IL1F6, C type lectins 4a3, 4e and 4N, ANKRD1, CCL2, CCL5, CXCL13, FCGR3, SERPINA10, ITGAM, TWEAK receptor, DNASE1, TIMP1 and VCAM-1 as demonstrated by microarray analysis. Because several cytokines either were not on the chip or did not pass the cut-off value, these were tested by PCR. IL6 and IL-10 signal through STAT3; these cytokines were tested and were upregulated in all three strains. Similarly, upregulation of IL-1β, TNFα and IFNγ was found in all three strains. Increased expression of IL17 was found in 50-85% of the mice in all three strains but reached significance only in the NZW/BXSB strain. The NZM2410 strain did not express IL-12p40, IL-21 or BAFF but was the only strain with increased expression of IL-18.

We next confirmed that the two strains with proliferative nephritis shared a profile consistent with inflammatory cell infiltration and endothelial activation. Genes in this set included chemokines, chemokine receptors, the actin cytoskeleton remodeling protein Coronin 1A, the vascular NADPH oxidase CYBB, LY86, CD40, BAFF, TLR9 and T cell molecules CD52, LIGHT, IL-21 and ICOS. Genes significantly downregulated in the kidneys of these two strains included the superoxide dismutases SOD2 and SOD3, the mitochondrial enzymes CLYBL and COX15, and estrogen related receptor β. Both strains manifested downregulation of the PAR domain basic leucine zipper transcription factors DBP, and HLF that control genes involved in lipid metabolism and detoxification [28]; downregulation of TEF, a gene with similar function was seen in NZW/BXSB. The NZM2410 mouse had less upregulation of CXC chemokines and their receptors than the other two strains and despite upregulation of CC chemokines, had only minimally increased expression of several of the corresponding chemokine receptors, consistent with the significantly lesser degree of inflammatory cell infiltration in this strain.

Unique genes regulated in NZB/W mice included the chemokine/chemokine receptor pair CCL20 and CCR6. Since we have previously identified Tregs in renal lymphoid aggregates of NZB/W mice [16] and Tregs express CCR6 we tested for FOXP3 expression. FOXP3 was upregulated only in NZB/W mice and correlated significantly with CCR6 expression (r value 0.87, p<0.001). In contrast, there was no correlation of CCR6 expression with IL-17A expression (not shown). Similarly, L selectin, a marker expressed on T cells in lymphoid aggregates [16] and CD138, a plasma cells marker, were found only in the NZB/W strain. Downregulation of nephrin was found only in NZM2410 mice reflecting podocyte loss associated with glomerulosclerosis. This strain also expressed ER stress markers ATF6 and RTN4. We confirmed the cell proliferation profile in the NZW/BXSB strain, with upregulation of the chromatin remodeling protein HELLS (helicase) and KI67. This strain also expressed the Th2 cell attracting chemokines CCL1, CCL17 and CCL22, perhaps reflecting the high degree of dendritic cell infiltration in this strain. A summary of the unique and shared pathogenic pathways identified in the three strains is shown in **Figure 4B**.

### Identification of proliferating cells In NZW/BXSB mice

**Figure 5:**
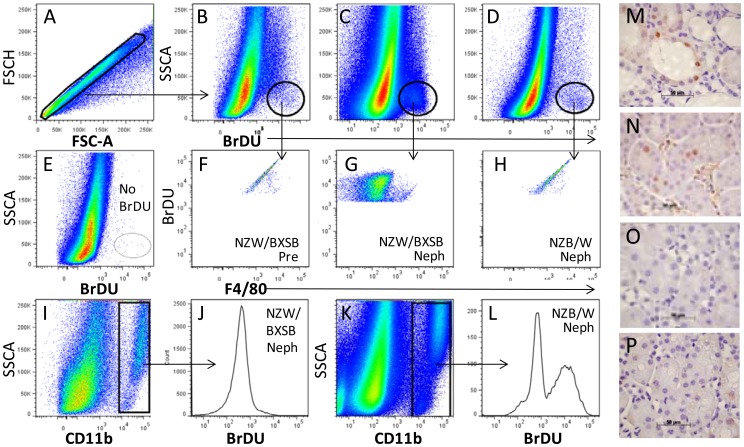
A–L. Flow cytometry analysis of proliferating cells. After gating for singlets (A), whole kidney cells from prenephritic (Pre - B, F) and nephritic (Neph - C, G, I, J) NZW/BXSB and nephritic NZB/W (D, H, K, L) mice were analyzed for BrDU incorporation. A BrDU^+^/F4/80^−^ population is seen only in nephritic NZW/BXSB mice (F–H). Proliferating CD11b^+^/F4/80^+^ macrophages are observed only in nephritic NZB/W mice (I–L). A non-BrDU treated control is shown in E. M–P. Immunohistochemistry of kidneys from nephritic NZW/BXSB (M, N), 8 week NZW/BXSB (O), and nephritic NZB/W (P) mice stained with antibodies to Ki67 (M) and PCNA (N–P). 40× magnification. Data are representative of 3 mice per stain.

To determine which cells were proliferating in nephritic NZW/BXSB mice we analyzed renal cells by flow cytometry after BrDU feeding. We found a significant increase in CD11b-/BrDU+ cells in nephritic compared with either young NZW/BXSB (4.1+/− 2.0 vs. 0.3+/− 0% total renal cells; p<0.05) or nephritic NZB/W mice (0.7+/− 0.3%; p<0.05 – **Figure 5A–E**) or NZM2410 mice (not shown). The BrDU+ cells were negative for lymphoid and macrophage/DC markers by flow cytometry (**Figure 5F–H** and not shown). In contrast, BrDU uptake in NZB/W kidneys was restricted to CD11b+ cells (**Figure 5I–L**). To localize the proliferating cells in nephritic NZW/BXSB kidneys we stained them with anti-Ki67 and anti-PCNA and showed a pattern consistent with tubular proliferation/regeneration (**Figure 5M–P**).

## Discussion

Lupus nephritis is a serious complication of SLE in which glomerular immune complex deposition triggers a cascade of inflammatory events that can lead to irreversible renal damage. Heterogeneity in the degree and type of peri-glomerular and tubulointerstitial inflammation, macrophage infiltration, and non-immune responses to inflammation and hypoxia may all contribute to the current difficulty in achieving or predicting treatment responses.

Because human renal tissue is rarely obtained either before treatment or on more than one occasion, mouse models of lupus are useful for addressing mechanisms of tissue damage in lupus nephritis. We have previously reported an analysis of the shared molecular profiles between kidneys from three murine models and human lupus nephritis biopsies. Using a low stringency for differential gene expression we found that 25–32% of the regulated genes in mice were also regulated in SLE renal biopsies and that both shared and unique features of the murine models overlapped with the human disease [10]. A limitation of the prior study is that the human samples were obtained after the initiation of immunosuppressive therapy, which likely modulated their inflammatory profile. In this study we compared the full molecular profiles and the derived transcriptional networks of the three mouse models of SLE nephritis with each other so as to identify mechanisms for disease pathogenesis, potential biomarkers for disease phenotype and potential therapeutic targets that can be tested in the appropriate model.

Despite differences in histologic appearance among the three models, 23–36% of the total gene set was shared among the three strains, reflecting known pathogenic processes downstream of immune complex deposition such as complement activation, macrophage and endothelial cell activation, and processes involved in tissue damage and remodeling. Among the 263 genes regulated in all three models, STAT3 was the top transcription factor, having a binding site in the promoter regions of 60 of those 263. STAT3 acts as a master regulator of cell metabolism and is activated by many cytokines and growth factors including type I interferons, IL10, and the IL-6 family of cytokines; by real-time PCR we confirmed increased renal expression of both IL-6 and IL-10 in all three lupus strains as well as a panel of IFN-inducible genes. Activation of STAT3 is associated with many forms of renal injury and the JAK2/STAT3 pathway has been implicated in the progression of renal fibrosis in several models of renal disease [29]. A recent study showed that a JAK2 inhibitor reduces renal STAT3 phosphorylation and prolongs survival in 7 month old NZB/W mice [30]. Importantly, 30 and 25 of the 60 Stat3 regulated genes identified in the mice were regulated in glomeruli and tubulointerstitium of human SLE renal biopsies respectively (**Table S2**), suggesting that Jak inhibitors may be a potential therapeutic approach for human SLE nephritis.

The most highly upregulated cytokine was IL1F6 (IL-36A), a member of the IL-1 family that is expressed by epithelial cells in the distal convoluted tubules and cortical collecting ducts of mice with SLE [31]. IL1F6 induces expression of IL-6 in epithelial cells and mediates a decrease in adhesion and loss of cuboidal morphology in a renal epithelial cell line [31]. IL1F6 is pathogenic when expressed in the skin and is highly expressed in human psoriasis [32]. In all three strains the level of expression of IL1F6 as assessed by qPCR correlated significantly with LCN-2 and HAVCR1, two markers of proximal tubular damage (r values for NZM2410: 0.5562, p = 0.0021 and 0.8489, p<0.0001; r values for NZB/W: 0.3729, p = 0.0077 and 0.8186, p<0.0001; r values for NZW/BXSB: 0.9063, p<0.0001 and 0.9277, p<0.0001), showing that progressive nephritis is associated with damage to the entire tubular system. Importantly, significant upregulation of these three markers did not occur in the human biopsies [10], indicating a lesser degree of tubular damage in treated individuals; this is consistent with our previous findings that glomerular damage precedes tubulointerstitial damage in NZB/W mice, that upregulation of LCN-2 and HAVCR1 occurs only in the late stages of SLE nephritis in NZB/W and NZW/BXSB mice, and that there is marked downregulation of LCN-2, IL1F6 and HAVCR1 in the kidneys of mice in remission ([11], and manuscript in preparation).

Another shared upregulated cytokine was IL-34, an alternate ligand for the CSF1 receptor. This cytokine is expressed by neurons and keratinocytes and directs the differentiation of macrophages in brain and skin [33], [34]. In the skin, IL-34 is not required for infiltration or differentiation of inflammatory monocytes but it is necessary for the maintenance of Langerhans cells after the resolution of inflammation [33]. A small amount of IL-34 is constitutively expressed by kidney proximal tubules [34]. Whether there is a role for IL-34 either in the maintenance of local renal macrophages or in the propagation of renal inflammation is not yet known.

We also showed that the downregulation of DNAse1 previously described in NZB/W mice and in human Class IV lupus nephritis [35] occurs in all three murine models. While it is still not known how renal DNAse1 expression is regulated, its lack has been postulated to enhance the size of chromatin deposits in the kidney and therefore the amount of autoantibody deposition and the pro-inflammatory response to nucleic acids [36]. Other protective genes that were downregulated included the superoxide dismutase 2 that protects from tissue hypoxia [37], [38].

It was surprising that the two models with proliferative disease only shared a small number of additional genes. Since most of the renal tissue is derived from the interstitium, it is possible that some of the glomerular signature was diluted in the whole kidney sample. Analysis of isolated glomeruli should further address this possibility. Nevertheless, the shared expression of macrophage related genes in proliferative disease suggests that macrophage activation and interstitial accumulation contributes to the poor prognosis of this histologic subtype. Indeed, poor prognosis has been found to correlate best with macrophage infiltration and with total number of interstitial CD45+ cells in several studies of human SLE [39], [40]. We did not observe upregulation of genes that are highly expressed in human netting neutrophils [41], consistent with the low number of infiltrating neutrophils in these mouse strains.

Genes associated with coagulation/fibrinolysis were more highly expressed in NZB/W and NZM2410 mice than in NZW/BXSB. One of these, PAI, is associated with renal fibrosis; targeting of PAI protects from renal fibrosis in a variety of models [42]. Preliminary studies have indicated that inhibition of PAI is protective in the NZB/W model, an appropriate model to test this therapeutic intervention (Naiman et. al. Abstract 2681, American College of Rheumatology Annual Meeting, Nov 2012). Another downregulated gene in both NZB/W and NZM2410 mice was Serpina6, a corticosteroid binding globulin that is expressed in proximal tubules only in female kidneys [43] where it is a regulator of renal osmotic pressure.

Although the kidneys receive a large amount of blood flow, tubular blood flow is easily compromised because it depends on upstream blood flow through the glomeruli. Oxidative stress alters mitochondria and electrolyte transport efficiency and this in turn stimulates interstitial fibrosis and may induce or worsen hypertension [44].The NZB/W and NZM2410 strains shared a mitochondrial dysfunction signature; importantly, this signature was shared by human renal SLE biopsies [10]. Targeting oxidative stress may be another approach to protect the kidney in SLE patients and can be tested in the NZB/W and NZM2410 models.

We identified a set of epithelial genes shared between nephritic NZM2410 and NZW/BXSB mice. These included Claudin1, a protein associated with tight junctions of renal epithelial cells [45], Slp-2a that regulates tubular apical polarization [46] and clusterin, an inhibitor of tight junction disintegration by MMPs [47]. These strains also expressed high levels of the CXCR2 ligands CXCL1 and CXCL2. CXCL1 is produced by renal epithelial cells in response to HGF stimulation and helps to mediate tubule repair [48]. This signature, in sum suggests an alteration in differentiation state of the renal epithelium of these two strains.

Unique features of each model were also highly informative. The accumulation of lymphoid aggregates, that occurs in some human SLE patients [8], [49], was found predominantly in the NZB/W model and was manifested by expression of adhesion molecules, activated T cell markers and accumulation of regulatory T cells and plasma cells. A B cell signature similarly occurs in only a subset of human SLE renal biopsies ([49] and our unpublished data) although neither T nor B cell numbers were found to correlate with prognosis [40]. While all three models manifested features of metabolic stress, this was most marked in the NZM2410 model in which there was little accumulation of lymphoid or myeloid cells. Tubular regeneration was noted in the NZW/BXSB model. Finally, we observed a decrease in the circadian transcription factors, DBP, HLF and TEF in NZW/BXSB and, to a lesser extent in NZB/W mice. These transcription factors regulate proteins that contribute to detoxification of a variety of substrates including cyclophosphamide [28]. Downregulation of circadian transcription factors can be mediated by cytokines such as TNF [50] and TGFβ [51] and dysregulation of the metabolic clock may exacerbate inflammation [52]. Importantly, dysregulation of these factors may have clinical significance with respect to toxicity of drugs used to treat active nephritis. Our data suggests that more work is required to determine the effects of nephritis on renal circadian functions.

Activation of intrinsic protective mechanisms also occurred in the nephritic kidneys. Examples include upregulation of SOCS3, an inactivator of cytokines, SERPINA3G (Spi-2A) that protects from caspase mediated cell death [53], NOX2 that may protect against ischemia and oxidative stress [54] and clusterin that protects against renal fibrosis [55]. A challenge in SLE nephritis therapy is maintaining naturally protective pathways while antagonizing pro-inflammatory pathways, so as to promote healing rather than scarring.

Our approach shows that in addition to identifying broadly applicable pathogenic features of SLE nephritis, we can also predict the appropriate mouse model to study specific pathologic features of and therapeutic approaches for lupus nephritis. Our study identifies both immune and non-immune pathways of renal damage that could be independently targeted in a personalized fashion.

## Supporting Information

Table S1 List of regulated unique and shared genes (human orthologs) in the nephritic vs. prenephritic mice of the NZB/W, NZM2410 and NZW/BXSB strains.
(DOCX)

Table S2 Stat3 regulated genes in NZB/W, NZM2410, NZW/BXSB and in human LN renal biopsies.
(DOCX)

Table S3 Comparison of microarray data and qPCR data.
(DOC)
