# Supplementary material for: Comparative Transcriptional Profiling of 3 Murine Models of SLE Nephritis Reveals Both Unique and Shared Regulatory Networks
Source: PLoS One. 2013 Oct 22;8(10):e77489. doi: 10.1371/journal.pone.0077489 (PMC3805607; doi:10.1371/journal.pone.0077489)
Supplement: Table S1 — List of regulated unique and shared genes (human orthologs) in the nephritic vs. prenephritic mice of the NZB/W, NZM2410 and NZW/BXSB strains. (DOCX) [file pone.0077489.s001.docx]

**Table S1:** List of regulated unique and shared genes (human orthologs) in the nephritic vs. prenephritic mice of the NZB/W, NZM2410 and NZW/BXSB strains (defined filter criteria: q-value <0.001, fold-change ≥1.4 for the up-regulated genes and ≤0.7 for the down-regulated genes). The genes passing the filter criteria are highlighted in bold. *np: genes not passing the Affymetrix negative controls cut-off.*

| **A. 349 genes regulated only in NZB/W 36 weeks old vs. 16 wks old.** | | | | | | | | | | | | | |
| --- | --- | --- | --- | --- | --- | --- | --- | --- | --- | --- | --- | --- | --- |
| Human Entrez Gene ID | | Human Gene symbol | **NZB/W** | | | | | **NZM2410** | | | **NZW/BXSB** | | |
|  |  |  | Fold-change | | | q-value | | Fold-change | | q-value | Fold-change | q-value |  |
| 29974 | | A1CF | **0.55** | | | **0.0000** | | 0.65 | | 0.0179 | 0.80 | 0.0176 |  |
| 13 | | AADAC | **0.64** | | | **0.0004** | | 0.64 | | 0.0012 | 0.96 | 0.1927 |  |
| 9619 | | ABCG1 | **1.50** | | | **0.0004** | | 1.11 | | 0.1017 | 1.20 | 0.0003 |  |
| 9429 | | ABCG2 | **0.69** | | | **0.0000** | | 0.78 | | 0.0036 | 0.98 | 0.2499 |  |
| 10449 | | ACAA2 | **0.66** | | | **0.0007** | | 0.70 | | 0.0015 | 0.58 | 0.0013 |  |
| 84129 | | ACAD11 | **0.68** | | | **0.0006** | | 0.71 | | 0.0050 | 0.75 | 0.0046 |  |
| 122970 | | ACOT4 | **0.65** | | | **0.0000** | | 0.65 | | 0.0040 | 0.88 | 0.0984 |  |
| 84532 | | ACSS1 | **0.55** | | | **0.0000** | | 0.61 | | 0.0012 | 0.73 | 0.0000 |  |
| 100 | | ADA | **1.72** | | | **0.0000** | | 1.25 | | 0.0818 | 1.17 | 0.0015 |  |
| 120 | | ADD3 | **0.64** | | | **0.0000** | | 0.68 | | 0.0012 | 0.94 | 0.1292 |  |
| 185 | | AGTR1 | **0.71** | | | **0.0000** | | 0.79 | | 0.0322 | 1.11 | 0.0310 |  |
| 23382 | | AHCYL2 | **0.62** | | | **0.0000** | | 0.70 | | 0.0017 | 0.83 | 0.0198 |  |
| 80709 | | AKNA | **1.57** | | | **0.0000** | | 1.46 | | 0.0019 | 1.22 | 0.0003 |  |
| 64577 | | ALDH8A1 | **0.59** | | | **0.0000** | | 0.57 | | 0.0010 | 0.83 | 0.0003 |  |
| 288 | | ANK3 | **0.71** | | | **0.0000** | | 0.97 | | 0.3540 | 0.98 | 0.2832 |  |
| 81573 | | ANKRD13C | **0.64** | | | **0.0000** | | 0.61 | | 0.0014 | 0.87 | 0.0059 |  |
| 9582 | | APOBEC3B | **1.51** | | | **0.0000** | | 1.20 | | 0.0407 | 1.19 | 0.0011 |  |
| 55937 | | APOM | **0.61** | | | **0.0004** | | 0.66 | | 0.0121 | 0.86 | 0.0474 |  |
| 83478 | | ARHGAP24 | **0.67** | | | **0.0000** | | 0.90 | | 0.1691 | 1.08 | 0.1292 |  |
| 9938 | | ARHGAP25 | **1.54** | | | **0.0000** | | 1.26 | | 0.0654 | 1.34 | 0.0000 |  |
| 257106 | | ARHGAP30 | **1.43** | | | **0.0004** | | 1.28 | | 0.0084 | 1.17 | 0.0004 |  |
| 57412 | | AS3MT | **0.64** | | | **0.0000** | | 0.90 | | 0.1341 | 0.92 | 0.0017 |  |
| 526 | | ATP6V1B2 | **0.69** | | | **0.0000** | | 0.73 | | 0.0004 | 0.93 | 0.0432 |  |
| 55024 | | BANK1 | **1.80** | | | **0.0004** | | 1.19 | | 0.1341 | np | | |
| 10538 | | BATF | **1.46** | | | **0.0004** | | 1.14 | | 0.1590 | 1.10 | 0.0388 |  |
| 11177 | | BAZ1A | **1.49** | | | **0.0000** | | 1.51 | | 0.0036 | 1.04 | 0.3403 |  |
| 54796 | | BNC2 | **0.51** | | | **0.0000** | | 0.66 | | 0.0019 | 0.92 | 0.1870 |  |
| 10380 | | BPNT1 | **0.58** | | | **0.0000** | | 0.63 | | 0.0017 | 0.76 | 0.0000 |  |
| 695 | | BTK | **1.57** | | | **0.0006** | | 1.25 | | 0.0179 | 1.25 | 0.0035 |  |
| 151888 | | BTLA | **2.37** | | | **0.0007** | | 1.41 | | 0.2327 | 1.30 | 0.0346 |  |
| 28970 | | C11orf54 | **0.70** | | | **0.0007** | | 0.85 | | 0.0891 | 0.84 | 0.0003 |  |
| 51501 | | C11orf73 | **0.71** | | | **0.0000** | | 0.83 | | 0.0087 | 0.85 | 0.0668 |  |
| 79415 | | C17orf62 | **1.54** | | | **0.0000** | | 1.26 | | 0.0079 | 1.00 | 0.9999 |  |
| 199675 | | C19orf59 | **1.57** | | | **0.0000** | | 1.20 | | 0.0966 | 1.12 | 0.0533 |  |
| 79630 | | C1orf54 | **1.43** | | | **0.0007** | | 1.16 | | 0.3826 | 1.22 | 0.0024 |  |
| 715 | | C1R | **1.77** | | | **0.0000** | | 1.75 | | 0.0070 | 1.30 | 0.0017 |  |
| 716 | | C1S | **1.72** | | | **0.0000** | | 1.76 | | 0.0031 | 1.32 | 0.0000 |  |
| 389073 | | C2orf80 | **0.58** | | | **0.0000** | | 0.67 | | 0.0133 | 1.10 | 0.2922 |  |
| 57150 | | C6orf162 | **0.70** | | | **0.0000** | | 0.73 | | 0.0009 | 0.87 | 0.0456 |  |
| 760 | | CA2 | **0.66** | | | **0.0000** | | 0.84 | | 0.0029 | 0.91 | 0.0236 |  |
| 822 | | CAPG | **1.50** | | | **0.0006** | | 1.55 | | 0.0092 | 1.49 | 0.0019 |  |
| 834 | | CASP1 | **1.97** | | | **0.0000** | | 1.51 | | 0.0029 | 1.34 | 0.0011 |  |
| 847 | | CAT | **0.65** | | | **0.0000** | | 0.75 | | 0.0012 | 0.82 | 0.0000 |  |
| 90060 | | CCDC120 | **1.43** | | | **0.0000** | | 1.97 | | 0.0028 | 1.37 | 0.0005 |  |
| 55704 | | CCDC88A | **1.42** | | | **0.0006** | | 1.15 | | 0.2267 | 1.32 | 0.0014 |  |
| 55297 | | CCDC91 | **0.69** | | | **0.0000** | | 0.80 | | 0.0129 | 0.88 | 0.0801 |  |
| 6349 | | CCL3L1 | **1.67** | | | **0.0000** | | np | | | 1.19 | 0.0357 |  |
| 6349 | | CCL3L1 | **1.67** | | | **0.0000** | | np | | | 1.19 | 0.0357 |  |
| 894 | | CCND2 | **1.53** | | | **0.0006** | | 1.07 | | 0.3754 | 1.08 | 0.2382 |  |
| 1235 | | CCR6 | **1.48** | | | **0.0006** | | 1.04 | | 0.3922 | 1.00 | 0.9999 |  |
| 914 | | CD2 | **1.65** | | | **0.0004** | | np | | | 1.07 | 0.3191 |  |
| 933 | | CD22 | **1.46** | | | **0.0000** | | 1.00 | | 0.9999 | 1.05 | 0.3320 |  |
| 940 | | CD28 | **1.48** | | | **0.0004** | | np | | | np | | |
| 10871 | | CD300C | **1.72** | | | **0.0000** | | 1.48 | | 0.0061 | 1.31 | 0.0000 |  |
| 951 | | CD37 | **1.82** | | | **0.0000** | | 1.05 | | 0.4233 | 1.05 | 0.3467 |  |
| 915 | | CD3D | **2.28** | | | **0.0000** | | 1.61 | | 0.0057 | 1.31 | 0.0023 |  |
| 921 | | CD5 | **1.56** | | | **0.0004** | | 1.42 | | 0.0082 | 1.20 | 0.0638 |  |
| 974 | | CD79B | **4.10** | | | **0.0000** | | 1.27 | | 0.0043 | 1.26 | 0.0273 |  |
| 9308 | | CD83 | **2.30** | | | **0.0000** | | 2.22 | | 0.0015 | 1.32 | 0.0168 |  |
| 942 | | CD86 | **1.54** | | | **0.0000** | | 1.72 | | 0.0010 | 1.35 | 0.0003 |  |
| 22918 | | CD93 | **1.40** | | | **0.0000** | | 1.06 | | 0.3540 | 1.21 | 0.0000 |  |
| 976 | | CD97 | **1.43** | | | **0.0000** | | 1.32 | | 0.0066 | 1.22 | 0.0003 |  |
| 51654 | | CDK5RAP1 | **0.71** | | | **0.0000** | | 0.77 | | 0.0057 | 0.88 | 0.0428 |  |
| 8814 | | CDKL1 | **0.62** | | | **0.0000** | | 0.75 | | 0.0337 | 0.74 | 0.0009 |  |
| 634 | | CEACAM1 | **0.66** | | | **0.0000** | | 0.65 | | 0.0040 | 0.87 | 0.0289 |  |
| 1051 | | CEBPB | **1.73** | | | **0.0007** | | 1.46 | | 0.0407 | 1.17 | 0.2259 |  |
| 1990 | | CELA1 | **0.53** | | | **0.0000** | | 0.61 | | 0.0073 | 1.16 | 0.0496 |  |
| 3075 | | CFH | **1.53** | | | **0.0000** | | 1.26 | | 0.3091 | 1.17 | 0.1747 |  |
| 400916 | | CHCHD10 | **0.70** | | | **0.0000** | | 0.73 | | 0.0005 | 0.92 | 0.0032 |  |
| 1116 | | CHI3L1 | **1.54** | | | **0.0007** | | 1.08 | | 0.3166 | 1.01 | 0.9999 |  |
| 56994 | | CHPT1 | **0.70** | | | **0.0007** | | 0.68 | | 0.0014 | 0.77 | 0.0000 |  |
| 54921 | | CHTF8 | **0.69** | | | **0.0004** | | 1.17 | | 0.2742 | 1.42 | 0.0057 |  |
| 9071 | | CLDN10 | **0.67** | | | **0.0000** | | 0.71 | | 0.0073 | 0.93 | 0.1381 |  |
| 112616 | | CMTM7 | **1.58** | | | **0.0000** | | 1.23 | | 0.0004 | 1.37 | 0.0000 |  |
| 83716 | | CRISPLD2 | **1.65** | | | **0.0004** | | 1.43 | | 0.0232 | 1.27 | 0.1626 |  |
| 1428 | | CRYM | **0.62** | | | **0.0000** | | 0.96 | | 0.3455 | 1.04 | 0.3373 |  |
| 1439 | | CSF2RB | **1.56** | | | **0.0000** | | np | | | 1.38 | 0.0003 |  |
| 1503 | | CTPS | **1.77** | | | **0.0000** | | 1.62 | | 0.0028 | 1.21 | 0.0268 |  |
| 1510 | | CTSE | **1.59** | | | **0.0006** | | 1.21 | | 0.0156 | 0.97 | 0.2259 |  |
| 9547 | | CXCL14 | **2.07** | | | **0.0000** | | 1.27 | | 0.0583 | 1.29 | 0.0047 |  |
| 4283 | | CXCL9 | **2.43** | | | **0.0008** | | 1.63 | | 0.0474 | 1.71 | 0.0129 |  |
| 7852 | | CXCR4 | **2.25** | | | **0.0000** | | 1.36 | | 0.2100 | 1.01 | 0.3749 |  |
| 80777 | | CYB5B | **0.66** | | | **0.0000** | | 0.81 | | 0.0407 | 0.88 | 0.0176 |  |
| 1537 | | CYC1 | **0.69** | | | **0.0000** | | 0.79 | | 0.0013 | 0.94 | 0.0520 |  |
| 66002 | | CYP4F12 | **0.67** | | | **0.0000** | | 0.73 | | 0.0242 | 0.77 | 0.0005 |  |
| 85443 | | DCLK3 | **0.69** | | | **0.0000** | | 0.72 | | 0.0000 | 0.78 | 0.0000 |  |
| 1634 | | DCN | **1.55** | | | **0.0000** | | 0.89 | | 0.3377 | 1.05 | 0.3577 |  |
| 1652 | | DDT | **0.71** | | | **0.0007** | | 0.75 | | 0.0161 | 0.95 | 0.1358 |  |
| 91319 | | DERL3 | **1.49** | | | **0.0008** | | 0.96 | | 0.4009 | 0.99 | 0.9999 |  |
| 115817 | | DHRS1 | **0.71** | | | **0.0000** | | 0.80 | | 0.0421 | 0.92 | 0.1000 |  |
| 1738 | | DLD | **0.63** | | | **0.0007** | | 0.66 | | 0.0013 | 0.77 | 0.0000 |  |
| 9093 | | DNAJA3 | **0.71** | | | **0.0000** | | 0.74 | | 0.0012 | 0.86 | 0.0000 |  |
| 51626 | | DYNC2LI1 | **0.70** | | | **0.0000** | | 0.86 | | 0.0112 | 0.99 | 0.9999 |  |
| 1999 | | ELF3 | **1.41** | | | **0.0000** | | 1.70 | | 0.0036 | 1.34 | 0.0000 |  |
| 2028 | | ENPEP | **0.69** | | | **0.0004** | | 0.84 | | 0.0109 | 0.98 | 0.2890 |  |
| 133121 | | ENPP6 | **0.68** | | | **0.0000** | | 0.78 | | 0.0315 | 0.82 | 0.0003 |  |
| 953 | | ENTPD1 | **1.44** | | | **0.0000** | | 1.33 | | 0.0031 | 1.21 | 0.0594 |  |
| 2108 | | ETFA | **0.71** | | | **0.0000** | | 0.75 | | 0.0015 | 0.81 | 0.0003 |  |
| 55500 | | ETNK1 | **0.65** | | | **0.0000** | | 0.89 | | 0.0474 | 0.96 | 0.2771 |  |
| 2124 | | EVI2B | **1.62** | | | **0.0000** | | 1.51 | | 0.0378 | 1.40 | 0.0003 |  |
| 51466 | | EVL | **1.72** | | | **0.0000** | | 1.13 | | 0.2665 | 1.37 | 0.0000 |  |
| 54491 | | FAM105A | **1.66** | | | **0.0000** | | 1.22 | | 0.0556 | 1.26 | 0.0000 |  |
| 25854 | | FAM149A | **0.61** | | | **0.0000** | | 0.88 | | 0.0366 | 0.85 | 0.0681 |  |
| 338094 | | FAM151A | **0.52** | | | **0.0000** | | 0.47 | | 0.0012 | 1.02 | 0.9999 |  |
| 125228 | | FAM210A | **0.65** | | | **0.0000** | | 0.67 | | 0.0029 | 0.77 | 0.0011 |  |
| 55603 | | FAM46A | **1.66** | | | **0.0000** | | 1.56 | | 0.0028 | 1.14 | 0.0881 |  |
| 54855 | | FAM46C | **1.57** | | | **0.0006** | | 1.03 | | 0.4081 | 1.12 | 0.0281 |  |
| 51571 | | FAM49B | **1.45** | | | **0.0000** | | 1.40 | | 0.0000 | 1.33 | 0.0000 |  |
| 145773 | | FAM81A | **0.66** | | | **0.0000** | | 0.90 | | 0.3540 | 0.76 | 0.0011 |  |
| 2194 | | FASN | **0.61** | | | **0.0004** | | 0.46 | | 0.0045 | 0.94 | 0.2832 |  |
| 115350 | | FCRL1 | **1.59** | | | **0.0000** | | np | | | np | | |
| 2242 | | FES | **1.44** | | | **0.0000** | | 1.41 | | 0.0092 | 1.29 | 0.0000 |  |
| 221472 | | FGD2 | **1.49** | | | **0.0006** | | 1.44 | | 0.0212 | 1.24 | 0.0007 |  |
| 2268 | | FGR | **1.45** | | | **0.0000** | | 1.18 | | 0.0474 | 1.32 | 0.0000 |  |
| 2288 | | FKBP4 | **0.69** | | | **0.0000** | | 0.80 | | 0.0017 | 0.92 | 0.0881 |  |
| 2313 | | FLI1 | **1.42** | | | **0.0008** | | 1.12 | | 0.2065 | 1.06 | 0.2124 |  |
| 2348 | | FOLR1 | **0.69** | | | **0.0004** | | 0.67 | | 0.0032 | 0.91 | 0.0056 |  |
| 2355 | | FOSL2 | **1.47** | | | **0.0009** | | 2.00 | | 0.0015 | 1.04 | 0.3255 |  |
| 2357 | | FPR1 | **1.90** | | | **0.0000** | | 1.47 | | 0.0179 | 1.30 | 0.0000 |  |
| 2487 | | FRZB | **1.87** | | | **0.0000** | | 0.99 | | 0.9999 | 0.87 | 0.1512 |  |
| 10690 | | FUT9 | **0.62** | | | **0.0006** | | 0.59 | | 0.0012 | 0.83 | 0.0456 |  |
| 2534 | | FYN | **1.48** | | | **0.0000** | | 1.09 | | 0.3296 | 1.09 | 0.1870 |  |
| 2538 | | G6PC | **0.50** | | | **0.0000** | | 0.67 | | 0.0221 | 1.02 | 0.3714 |  |
| 2562 | | GABRB3 | **0.45** | | | **0.0000** | | 0.63 | | 0.0012 | 0.93 | 0.2952 |  |
| 2585 | | GALK2 | **0.66** | | | **0.0000** | | 0.85 | | 0.0087 | 0.94 | 0.0626 |  |
| 8811 | | GALR2 | **0.66** | | | **0.0000** | | 0.57 | | 0.0019 | np | | |
| 2595 | | GANC | **0.68** | | | **0.0007** | | 0.81 | | 0.0654 | 0.78 | 0.0000 |  |
| 2620 | | GAS2 | **0.56** | | | **0.0000** | | 0.66 | | 0.0077 | 0.89 | 0.1988 |  |
| 2628 | | GATM | **0.36** | | | **0.0000** | | 0.34 | | 0.0012 | 0.98 | 0.9999 |  |
| 2632 | | GBE1 | **0.70** | | | **0.0000** | | 0.79 | | 0.0161 | 0.82 | 0.0003 |  |
| 2650 | | GCNT1 | **0.61** | | | **0.0000** | | 0.66 | | 0.0012 | 0.83 | 0.0406 |  |
| 85476 | | GFM1 | **0.64** | | | **0.0000** | | 0.64 | | 0.0012 | 0.85 | 0.0039 |  |
| 55340 | | GIMAP5 | **1.74** | | | **0.0000** | | 1.39 | | 0.0242 | 1.12 | 0.1626 |  |
| 168537 | | GIMAP7 | **2.17** | | | **0.0004** | | 1.24 | | 0.1341 | 1.10 | 0.1270 |  |
| 2705 | | GJB1 | **0.62** | | | **0.0000** | | 0.68 | | 0.0028 | 0.62 | 0.0032 |  |
| 11010 | | GLIPR1 | **2.81** | | | **0.0000** | | 1.74 | | 0.0012 | 1.66 | 0.0032 |  |
| 2752 | | GLUL | **0.68** | | | **0.0000** | | 0.76 | | 0.0062 | 0.84 | 0.0016 |  |
| 10249 | | GLYAT | **0.68** | | | **0.0003** | | 0.85 | | 0.0184 | 0.96 | 0.1311 |  |
| 132158 | | GLYCTK | **0.66** | | | **0.0000** | | 0.63 | | 0.0028 | 0.87 | 0.0608 |  |
| 2764 | | GMFB | **0.70** | | | **0.0003** | | 0.91 | | 0.3455 | 0.90 | 0.0207 |  |
| 9535 | | GMFG | **1.65** | | | **0.0000** | | 1.21 | | 0.0966 | 1.20 | 0.0018 |  |
| 2841 | | GPR18 | **1.76** | | | **0.0000** | | 1.26 | | 0.0040 | 1.22 | 0.0571 |  |
| 10149 | | GPR64 | **1.48** | | | **0.0004** | | 3.37 | | 0.0048 | 1.34 | 0.0003 |  |
| 63940 | | GPSM3 | **1.66** | | | **0.0000** | | 1.10 | | 0.3296 | 1.16 | 0.0022 |  |
| 2954 | | GSTZ1 | **0.69** | | | **0.0007** | | 0.62 | | 0.0013 | 0.83 | 0.0019 |  |
| 9563 | | H6PD | **1.43** | | | **0.0007** | | 1.33 | | 0.0066 | 1.14 | 0.0219 |  |
| 3026 | | HABP2 | **0.53** | | | **0.0000** | | 0.83 | | 0.1163 | 1.15 | 0.0338 |  |
| 51179 | | HAO2 | **0.67** | | | **0.0004** | | 0.68 | | 0.0010 | 0.83 | 0.0003 |  |
| 3067 | | HDC | **0.43** | | | **0.0006** | | 0.74 | | 0.1017 | 0.99 | 0.3039 |  |
| 3087 | | HHEX | **1.41** | | | **0.0000** | | 1.16 | | 0.1491 | 0.99 | 0.9999 |  |
| 8347 | | HIST1H2BC | **0.63** | | | **0.0004** | | 0.83 | | 0.0684 | 0.87 | 0.0143 |  |
| 3112 | | HLA-DOB | **1.78** | | | **0.0000** | | np | | | np | | |
| 3134 | | HLA-F | **1.46** | | | **0.0000** | | 1.51 | | 0.0025 | 1.23 | 0.0029 |  |
| 23526 | | HMHA1 | **1.71** | | | **0.0000** | | 1.30 | | 0.0144 | 1.20 | 0.0000 |  |
| 3248 | | HPGD | **0.61** | | | **0.0007** | | 0.61 | | 0.0139 | 1.04 | 0.3564 |  |
| 10855 | | HPSE | **1.42** | | | **0.0000** | | 1.23 | | 0.0754 | 1.05 | 0.2223 |  |
| 3284 | | HSD3B2 | **0.57** | | | **0.0000** | | 0.50 | | 0.0028 | 0.96 | 0.2922 |  |
| 84329 | | HVCN1 | **1.52** | | | **0.0000** | | 1.13 | | 0.1394 | 1.23 | 0.0060 |  |
| 3400 | | ID4 | **0.63** | | | **0.0000** | | 0.64 | | 0.0012 | 0.67 | 0.0155 |  |
| 3419 | | IDH3A | **0.65** | | | **0.0000** | | 0.72 | | 0.0012 | 0.90 | 0.0456 |  |
| 8519 | | IFITM1 | **1.65** | | | **0.0006** | | 0.97 | | 0.3922 | 1.21 | 0.0668 |  |
| 152404 | | IGSF11 | **0.68** | | | **0.0004** | | 0.64 | | 0.0098 | 1.03 | 0.3191 |  |
| 10320 | | IKZF1 | **1.66** | | | **0.0000** | | 1.30 | | 0.0242 | 1.25 | 0.0003 |  |
| 22806 | | IKZF3 | **1.42** | | | **0.0006** | | np | | | 1.01 | 0.9999 |  |
| 3603 | | IL16 | **1.69** | | | **0.0000** | | 1.21 | | 0.0378 | 1.28 | 0.0009 |  |
| 8809 | | IL18R1 | **1.66** | | | **0.0000** | | 1.96 | | 0.0062 | 1.36 | 0.0003 |  |
| 3553 | | IL1B | **2.28** | | | **0.0000** | | 1.53 | | 0.0170 | 1.36 | 0.0325 |  |
| 3561 | | IL2RG | **3.54** | | | **0.0000** | | 1.52 | | 0.0437 | 2.03 | 0.0022 |  |
| 3575 | | IL7R | **1.59** | | | **0.0004** | | np | | | 1.23 | 0.0011 |  |
| 11213 | | IRAK3 | **1.42** | | | **0.0000** | | np | | | 1.28 | 0.0000 |  |
| 3662 | | IRF4 | **1.56** | | | **0.0000** | | 1.24 | | 0.0156 | 1.33 | 0.0000 |  |
| 3394 | | IRF8 | **1.72** | | | **0.0000** | | 1.71 | | 0.0104 | 1.16 | 0.0114 |  |
| 3676 | | ITGA4 | **1.58** | | | **0.0000** | | 1.33 | | 0.0101 | 1.21 | 0.0003 |  |
| 9424 | | KCNK6 | **1.48** | | | **0.0007** | | 1.17 | | 0.0133 | 1.25 | 0.0004 |  |
| 3783 | | KCNN4 | **1.56** | | | **0.0000** | | 1.07 | | 0.3922 | 1.16 | 0.0094 |  |
| 23514 | | KIAA0146 | **0.62** | | | **0.0000** | | 0.57 | | 0.0084 | 1.24 | 0.0095 |  |
| 57179 | | KIAA1191 | **0.66** | | | **0.0000** | | 0.82 | | 0.0101 | 0.91 | 0.0023 |  |
| 54627 | | KIAA1383 | **0.59** | | | **0.0006** | | 0.69 | | 0.0077 | 0.91 | 0.2413 |  |
| 85457 | | KIAA1737 | **0.69** | | | **0.0004** | | 0.84 | | 0.0511 | 0.87 | 0.0207 |  |
| 55605 | | KIF21A | **0.66** | | | **0.0000** | | 0.82 | | 0.0045 | 0.88 | 0.0046 |  |
| 89857 | | KLHL6 | **1.57** | | | **0.0000** | | 1.28 | | 0.0654 | 1.27 | 0.0000 |  |
| 3858 | | KRT10 | **0.68** | | | **0.0000** | | 0.80 | | 0.0036 | 0.92 | 0.1110 |  |
| 3856 | | KRT8 | **1.64** | | | **0.0000** | | 2.38 | | 0.0035 | 1.48 | 0.0015 |  |
| 8942 | | KYNU | **0.49** | | | **0.0000** | | 0.55 | | 0.0019 | 1.96 | 0.0316 |  |
| 3909 | | LAMA3 | **0.70** | | | **0.0000** | | 0.80 | | 0.0021 | 0.95 | 0.2153 |  |
| 27040 | | LAT | **1.53** | | | **0.0004** | | 1.23 | | 0.1394 | 1.10 | 0.2413 |  |
| 3932 | | LCK | **1.73** | | | **0.0000** | | 1.43 | | 0.0042 | 1.30 | 0.0027 |  |
| 3937 | | LCP2 | **1.42** | | | **0.0000** | | 1.26 | | 0.0062 | 1.24 | 0.0058 |  |
| 80774 | | LIMD2 | **1.53** | | | **0.0000** | | 1.10 | | 0.2598 | 1.05 | 0.2413 |  |
| 3988 | | LIPA | **0.63** | | | **0.0000** | | 0.72 | | 0.0000 | 1.03 | 0.3500 |  |
| 645177 | | LOC645177 | **1.66** | | | **0.0000** | | 1.26 | | 0.0583 | 1.33 | 0.0000 |  |
| 4015 | | LOX | **2.13** | | | **0.0004** | | 1.40 | | 0.0490 | 1.35 | 0.0418 |  |
| 9926 | | LPGAT1 | **0.71** | | | **0.0000** | | 0.94 | | 0.4009 | 0.86 | 0.0070 |  |
| 55144 | | LRRC8D | **0.71** | | | **0.0000** | | 0.82 | | 0.0027 | 0.94 | 0.1110 |  |
| 4050 | | LTB | **4.18** | | | **0.0000** | | 1.69 | | 0.0139 | 1.35 | 0.0065 |  |
| 4063 | | LY9 | **1.69** | | | **0.0000** | | 1.14 | | 0.1394 | 1.13 | 0.0377 |  |
| 57134 | | MAN1C1 | **1.42** | | | **0.0004** | | 1.27 | | 0.0536 | 1.27 | 0.0000 |  |
| 11184 | | MAP4K1 | **1.45** | | | **0.0006** | | 1.04 | | 0.4553 | 1.07 | 0.2648 |  |
| 9053 | | MAP7 | **0.64** | | | **0.0000** | | 0.73 | | 0.0000 | 0.87 | 0.0289 |  |
| 55700 | | MAP7D1 | **1.43** | | | **0.0007** | | 1.28 | | 0.0156 | 1.29 | 0.0000 |  |
| 9261 | | MAPKAPK2 | **1.41** | | | **0.0000** | | 1.24 | | 0.0151 | 1.07 | 0.2588 |  |
| 25840 | | METTL7A | **0.69** | | | **0.0006** | | 0.77 | | 0.0013 | 0.80 | 0.0049 |  |
| 10227 | | MFSD10 | **1.42** | | | **0.0000** | | 1.38 | | 0.0242 | 1.21 | 0.0005 |  |
| 4248 | | MGAT3 | **0.65** | | | **0.0000** | | 0.54 | | 0.0011 | 0.84 | 0.0213 |  |
| 117153 | | MIA2 | **0.62** | | | **0.0008** | | 1.12 | | 0.3754 | 0.85 | 0.0571 |  |
| 55586 | | MIOX | **0.57** | | | **0.0000** | | 0.65 | | 0.0221 | 0.92 | 0.1110 |  |
| 4311 | | MME | **0.55** | | | **0.0000** | | 0.87 | | 0.2465 | 0.72 | 0.0000 |  |
| 93380 | | MMGT1 | **0.69** | | | **0.0000** | | 0.81 | | 0.0092 | 0.91 | 0.0069 |  |
| 29074 | | MRPL18 | **0.67** | | | **0.0004** | | 0.67 | | 0.0010 | 0.84 | 0.0027 |  |
| 931 | | MS4A1 | **3.35** | | | **0.0004** | | np | | | 1.14 | 0.3450 |  |
| 139221 | | MUM1L1 | **0.70** | | | **0.0000** | | 0.78 | | 0.0718 | 1.02 | 0.9999 |  |
| 4615 | | MYD88 | **1.44** | | | **0.0000** | | 1.40 | | 0.0000 | 1.27 | 0.0000 |  |
| 51237 | | MZB1 | **4.17** | | | **0.0000** | | 1.04 | | 0.4151 | 1.37 | 0.0377 |  |
| 4702 | | NDUFA8 | **0.70** | | | **0.0000** | | 0.74 | | 0.0004 | 0.93 | 0.0207 |  |
| 4704 | | NDUFA9 | **0.68** | | | **0.0000** | | 0.72 | | 0.0009 | 0.84 | 0.0000 |  |
| 51103 | | NDUFAF1 | **0.71** | | | **0.0004** | | 0.72 | | 0.0012 | 0.92 | 0.1083 |  |
| 4817 | | NIT1 | **0.70** | | | **0.0000** | | 0.73 | | 0.0006 | 0.76 | 0.0000 |  |
| 9111 | | NMI | **1.51** | | | **0.0006** | | 1.69 | | 0.0017 | 1.38 | 0.0000 |  |
| 64802 | | NMNAT1 | **0.64** | | | **0.0000** | | 0.72 | | 0.0006 | 0.87 | 0.0038 |  |
| 26471 | | NUPR1 | **2.08** | | | **0.0000** | | 1.79 | | 0.0032 | 1.50 | 0.0035 |  |
| 116150 | | NUS1 | **0.58** | | | **0.0000** | | 0.67 | | 0.0349 | 0.83 | 0.0126 |  |
| 4942 | | OAT | **0.65** | | | **0.0000** | | 0.74 | | 0.0007 | 1.05 | 0.2382 |  |
| 64859 | | OBFC2A | **0.69** | | | **0.0000** | | 0.89 | | 0.2212 | 0.96 | 0.2437 |  |
| 4953 | | ODC1 | **0.62** | | | **0.0000** | | 0.69 | | 0.0050 | 0.77 | 0.1402 |  |
| 4976 | | OPA1 | **0.68** | | | **0.0000** | | 0.71 | | 0.0077 | 0.85 | 0.0000 |  |
| 200931 | | OSTalpha | **0.62** | | | **0.0004** | | 0.63 | | 0.0121 | 0.78 | 0.0710 |  |
| 5053 | | PAH | **0.55** | | | **0.0000** | | 0.59 | | 0.0012 | 0.94 | 0.1626 |  |
| 9060 | | PAPSS2 | **0.50** | | | **0.0000** | | 0.57 | | 0.0024 | 0.78 | 0.0046 |  |
| 54956 | | PARP16 | **0.71** | | | **0.0000** | | 0.80 | | 0.0005 | 0.92 | 0.1460 |  |
| 64081 | | PBLD | **0.71** | | | **0.0000** | | 0.74 | | 0.0109 | 0.73 | 0.0000 |  |
| 5096 | | PCCB | **0.70** | | | **0.0004** | | 0.72 | | 0.0008 | 0.86 | 0.0000 |  |
| 5136 | | PDE1A | **0.66** | | | **0.0000** | | 0.73 | | 0.0007 | 0.86 | 0.0126 |  |
| 5160 | | PDHA1 | **0.71** | | | **0.0000** | | 0.77 | | 0.0036 | 0.83 | 0.0000 |  |
| 79849 | | PDZD3 | **0.66** | | | **0.0000** | | 0.62 | | 0.0010 | 0.96 | 0.2525 |  |
| 5198 | | PFAS | **0.70** | | | **0.0000** | | 0.72 | | 0.0009 | 1.00 | 0.9999 |  |
| 5288 | | PIK3C2G | **0.48** | | | **0.0000** | | 0.53 | | 0.0024 | 0.80 | 0.0035 |  |
| 23533 | | PIK3R5 | **1.76** | | | **0.0000** | | 1.16 | | 0.2517 | 1.31 | 0.0003 |  |
| 5305 | | PIP4K2A | **1.60** | | | **0.0000** | | 1.41 | | 0.0016 | 1.21 | 0.0039 |  |
| 9600 | | PITPNM1 | **1.52** | | | **0.0000** | | 1.45 | | 0.0040 | 1.18 | 0.0003 |  |
| 26279 | | PLA2G2D | **1.51** | | | **0.0000** | | np | | | np | | |
| 7941 | | PLA2G7 | **1.66** | | | **0.0000** | | 1.05 | | 0.4703 | 1.31 | 0.0083 |  |
| 5329 | | PLAUR | **1.49** | | | **0.0006** | | 2.03 | | 0.0089 | 1.30 | 0.0249 |  |
| 5450 | | POU2AF1 | **8.20** | | | **0.0000** | | np | | | 2.10 | 0.0017 |  |
| 10891 | | PPARGC1A | **0.53** | | | **0.0000** | | 0.81 | | 0.1795 | 0.77 | 0.0046 |  |
| 151742 | | PPM1L | **0.68** | | | **0.0000** | | 1.00 | | 0.9999 | 0.81 | 0.0017 |  |
| 170954 | | PPP1R18 | **1.69** | | | **0.0000** | | 1.37 | | 0.0124 | 1.28 | 0.0002 |  |
| 57580 | | PREX1 | **1.57** | | | **0.0000** | | 1.20 | | 0.0684 | 1.32 | 0.0000 |  |
| 5563 | | PRKAA2 | **0.68** | | | **0.0000** | | 0.86 | | 0.0133 | 0.96 | 0.2041 |  |
| 9051 | | PSTPIP1 | **1.50** | | | **0.0006** | | 1.16 | | 0.0556 | 1.29 | 0.0000 |  |
| 9317 | | PTER | **0.71** | | | **0.0006** | | 0.77 | | 0.0046 | 0.95 | 0.2525 |  |
| 5733 | | PTGER3 | **0.60** | | | **0.0000** | | 0.78 | | 0.0437 | 0.86 | 0.0189 |  |
| 5745 | | PTH1R | **0.70** | | | **0.0006** | | 0.67 | | 0.0046 | 0.89 | 0.0406 |  |
| 5770 | | PTPN1 | **1.43** | | | **0.0000** | | 1.24 | | 0.0006 | 1.13 | 0.0084 |  |
| 26469 | | PTPN18 | **1.53** | | | **0.0000** | | 1.30 | | 0.0151 | 1.27 | 0.0000 |  |
| 26191 | | PTPN22 | **2.60** | | | **0.0000** | | 2.03 | | 0.0139 | 0.86 | 0.2124 |  |
| 5777 | | PTPN6 | **1.70** | | | **0.0000** | | 1.15 | | 0.1206 | 1.18 | 0.0173 |  |
| 25945 | | PVRL3 | **0.71** | | | **0.0000** | | 0.91 | | 0.1440 | 0.99 | 0.9999 |  |
| 22821 | | RASA3 | **1.62** | | | **0.0000** | | 0.93 | | 0.1206 | 1.25 | 0.0000 |  |
| 83937 | | RASSF4 | **1.90** | | | **0.0000** | | 1.79 | | 0.0010 | 1.18 | 0.0533 |  |
| 5930 | | RBBP6 | **0.71** | | | **0.0000** | | 0.74 | | 0.0036 | 0.84 | 0.0064 |  |
| 23180 | | RFTN1 | **1.41** | | | **0.0000** | | 1.06 | | 0.4703 | 1.27 | 0.0000 |  |
| 5996 | | RGS1 | **1.97** | | | **0.0000** | | 1.29 | | 0.0184 | 1.71 | 0.0027 |  |
| 391 | | RHOG | **1.41** | | | **0.0000** | | 1.27 | | 0.0013 | 1.30 | 0.0000 |  |
| 9616 | | RNF7 | **0.70** | | | **0.0006** | | 0.93 | | 0.2389 | 0.96 | 0.1770 |  |
| 80135 | | RPF1 | **0.69** | | | **0.0006** | | 0.93 | | 0.3166 | 0.96 | 0.1545 |  |
| 25939 | | SAMHD1 | **1.64** | | | **0.0000** | | 1.34 | | 0.0029 | 1.33 | 0.0000 |  |
| 64092 | | SAMSN1 | **1.53** | | | **0.0000** | | 1.30 | | 0.1017 | 1.22 | 0.0023 |  |
| 54440 | | SASH3 | **2.01** | | | **0.0000** | | 1.57 | | 0.0019 | 1.31 | 0.0047 |  |
| 950 | | SCARB2 | **1.52** | | | **0.0000** | | 1.22 | | 0.1097 | 1.16 | 0.0388 |  |
| 6402 | | SELL | **1.61** | | | **0.0004** | | 0.97 | | 0.4081 | 1.05 | 0.3255 |  |
| 6452 | | SH3BP2 | **1.78** | | | **0.0000** | | 1.50 | | 0.0040 | 0.94 | 0.9999 |  |
| 51246 | | SHISA5 | **1.43** | | | **0.0000** | | 1.30 | | 0.0315 | 1.06 | 0.0474 |  |
| 114836 | | SLAMF6 | **1.70** | | | **0.0000** | | 1.15 | | 0.0511 | 1.12 | 0.1000 |  |
| 56833 | | SLAMF8 | **1.56** | | | **0.0004** | | 1.42 | | 0.0139 | 1.35 | 0.0179 |  |
| 64849 | | SLC13A3 | **0.60** | | | **0.0000** | | 0.56 | | 0.0017 | 0.78 | 0.0023 |  |
| 151473 | | SLC16A14 | **0.55** | | | **0.0000** | | 0.61 | | 0.0221 | 0.67 | 0.0230 |  |
| 6582 | | SLC22A2 | **0.70** | | | **0.0008** | | 0.65 | | 0.0013 | 0.75 | 0.0011 |  |
| 9356 | | SLC22A6 | **0.68** | | | **0.0000** | | 0.61 | | 0.0049 | 0.80 | 0.0019 |  |
| 9376 | | SLC22A8 | **0.56** | | | **0.0000** | | 0.59 | | 0.0049 | 0.85 | 0.0751 |  |
| 10165 | | SLC25A13 | **0.62** | | | **0.0000** | | 0.74 | | 0.0066 | 0.88 | 0.0030 |  |
| 10166 | | SLC25A15 | **0.64** | | | **0.0000** | | 0.69 | | 0.0254 | 0.86 | 0.0089 |  |
| 10861 | | SLC26A1 | **0.61** | | | **0.0004** | | 0.66 | | 0.0170 | 0.98 | 0.9999 |  |
| 6514 | | SLC2A2 | **0.61** | | | **0.0000** | | 0.79 | | 0.0061 | 0.95 | 0.1230 |  |
| 54407 | | SLC38A2 | **1.63** | | | **0.0000** | | 1.70 | | 0.0049 | 1.05 | 0.3296 |  |
| 84102 | | SLC41A2 | **1.50** | | | **0.0000** | | 1.25 | | 0.0926 | 1.08 | 0.1460 |  |
| 29015 | | SLC43A3 | **1.51** | | | **0.0000** | | 1.22 | | 0.2100 | 1.06 | 0.3564 |  |
| 8671 | | SLC4A4 | **0.64** | | | **0.0000** | | 0.69 | | 0.0012 | 0.83 | 0.0024 |  |
| 159963 | | SLC5A12 | **0.54** | | | **0.0000** | | 0.52 | | 0.0012 | 0.89 | 0.0681 |  |
| 9056 | | SLC7A7 | **0.55** | | | **0.0000** | | 0.62 | | 0.0048 | 1.15 | 0.0119 |  |
| 10479 | | SLC9A6 | **0.70** | | | **0.0000** | | 0.82 | | 0.0608 | 0.84 | 0.0003 |  |
| 1.01E+08 | | SLFN12L | **1.51** | | | **0.0000** | | 1.35 | | 0.0048 | 1.26 | 0.0000 |  |
| 146857 | | SLFN13 | **1.58** | | | **0.0000** | | 1.20 | | 0.0179 | 1.12 | 0.0725 |  |
| 9353 | | SLIT2 | **0.67** | | | **0.0006** | | 0.83 | | 0.0556 | 0.82 | 0.0035 |  |
| 6590 | | SLPI | **2.75** | | | **0.0000** | | 1.63 | | 0.0170 | 1.21 | 0.0143 |  |
| 23676 | | SMPX | **0.67** | | | **0.0000** | | 0.60 | | 0.0012 | np | | |
| 8773 | | SNAP23 | **0.67** | | | **0.0004** | | 0.81 | | 0.0718 | 0.98 | 0.9999 |  |
| 6693 | | SPN | **1.42** | | | **0.0008** | | 1.15 | | 0.0851 | 1.09 | 0.1482 |  |
| 10418 | | SPON1 | **1.72** | | | **0.0000** | | 1.13 | | 0.3922 | 1.36 | 0.0094 |  |
| 92369 | | SPSB4 | **0.69** | | | **0.0000** | | 0.80 | | 0.0005 | 1.27 | 0.0005 |  |
| 6716 | | SRD5A2 | **0.68** | | | **0.0000** | | 0.83 | | 0.0556 | 0.72 | 0.0346 |  |
| 5552 | | SRGN | **1.77** | | | **0.0000** | | 1.01 | | 0.4553 | 1.20 | 0.0281 |  |
| 6480 | | ST6GAL1 | **1.51** | | | **0.0000** | | 1.42 | | 0.0024 | 1.33 | 0.0000 |  |
| 7903 | | ST8SIA4 | **1.69** | | | **0.0000** | | 1.53 | | 0.0050 | 1.37 | 0.0000 |  |
| 23166 | | STAB1 | **1.40** | | | **0.0000** | | 1.41 | | 0.0040 | 1.07 | 0.2353 |  |
| 8576 | | STK16 | **0.66** | | | **0.0000** | | 0.70 | | 0.0040 | 0.88 | 0.0984 |  |
| 252983 | | STXBP4 | **0.59** | | | **0.0000** | | 0.68 | | 0.0012 | 0.86 | 0.1203 |  |
| 6819 | | SULT1C2 | **0.62** | | | **0.0000** | | 0.76 | | 0.0031 | 1.10 | 0.2064 |  |
| 84144 | | SYDE2 | **0.66** | | | **0.0004** | | 0.76 | | 0.0121 | 0.87 | 0.0236 |  |
| 6850 | | SYK | **1.70** | | | **0.0000** | | 1.15 | | 0.1163 | 1.29 | 0.0004 |  |
| 374403 | | TBC1D10C | **1.80** | | | **0.0006** | | 1.17 | | 0.1745 | 1.11 | 0.1358 |  |
| 23102 | | TBC1D2B | **1.48** | | | **0.0000** | | 1.10 | | 0.3229 | 1.25 | 0.0003 |  |
| 6948 | | TCN2 | **0.68** | | | **0.0000** | | 0.73 | | 0.0046 | 0.94 | 0.0124 |  |
| 7980 | | TFPI2 | **0.67** | | | **0.0004** | | 0.69 | | 0.0144 | 1.05 | 0.9999 |  |
| 26520 | | TIMM9 | **0.68** | | | **0.0007** | | 0.75 | | 0.0036 | 0.97 | 0.2618 |  |
| 27283 | | TINAG | **0.63** | | | **0.0000** | | 0.63 | | 0.0016 | 0.96 | 0.2223 |  |
| 7099 | | TLR4 | **1.49** | | | **0.0000** | | 1.37 | | 0.0021 | 1.11 | 0.0919 |  |
| 53346 | | TM6SF1 | **1.81** | | | **0.0000** | | 1.12 | | 0.3826 | 1.24 | 0.0095 |  |
| 7108 | | TM7SF2 | **0.64** | | | **0.0000** | | 0.71 | | 0.0012 | 0.82 | 0.0024 |  |
| 135932 | | TMEM139 | **0.64** | | | **0.0000** | | 0.65 | | 0.0013 | 0.77 | 0.0000 |  |
| 134288 | | TMEM174 | **0.66** | | | **0.0000** | | 0.72 | | 0.0027 | 0.90 | 0.0418 |  |
| 729515 | | TMEM242 | **0.67** | | | **0.0000** | | 0.81 | | 0.0144 | 1.02 | 0.3763 |  |
| 169200 | | TMEM64 | **0.51** | | | **0.0000** | | 0.57 | | 0.0013 | 0.90 | 0.1545 |  |
| 137835 | | TMEM71 | **1.41** | | | **0.0000** | | 1.23 | | 0.1341 | 1.22 | 0.0003 |  |
| 25816 | | TNFAIP8 | **0.70** | | | **0.0000** | | 0.69 | | 0.0012 | 0.91 | 0.2064 |  |
| 79626 | | TNFAIP8L2 | **1.56** | | | **0.0000** | | 1.51 | | 0.0036 | 1.29 | 0.0000 |  |
| 9537 | | TP53I11 | **1.52** | | | **0.0000** | | 0.87 | | 0.1745 | 1.01 | 0.9999 |  |
| 94241 | | TP53INP1 | **1.57** | | | **0.0000** | | 1.38 | | 0.0006 | 1.20 | 0.0102 |  |
| 7185 | | TRAF1 | **1.54** | | | **0.0000** | | 1.77 | | 0.0012 | 1.21 | 0.0100 |  |
| 10906 | | TRAFD1 | **1.41** | | | **0.0000** | | 1.48 | | 0.0079 | 1.16 | 0.0011 |  |
| 84676 | | TRIM63 | **0.40** | | | **0.0000** | | 0.48 | | 0.0017 | 0.83 | 0.2735 |  |
| 81786 | | TRIM7 | **0.56** | | | **0.0000** | | 1.03 | | 0.9999 | 0.78 | 0.0084 |  |
| 7220 | | TRPC1 | **0.62** | | | **0.0000** | | 0.59 | | 0.0012 | 0.78 | 0.0019 |  |
| 150737 | | TTC30B | **0.66** | | | **0.0000** | | 0.72 | | 0.0028 | 1.00 | 0.9999 |  |
| 7277 | | TUBA4A | **0.71** | | | **0.0000** | | 0.80 | | 0.0073 | 1.01 | 0.9999 |  |
| 7318 | | UBA7 | **1.77** | | | **0.0000** | | 1.80 | | 0.0057 | 1.51 | 0.0011 |  |
| 7351 | | UCP2 | **1.85** | | | **0.0000** | | 2.01 | | 0.0051 | 0.73 | 0.0418 |  |
| 7365 | | UGT2B10 | **0.60** | | | **0.0000** | | 0.80 | | 0.0421 | 0.85 | 0.0668 |  |
| 7366 | | UGT2B15 | **0.68** | | | **0.0004** | | 0.79 | | 0.0349 | 0.83 | 0.0055 |  |
| 167127 | | UGT3A2 | **0.64** | | | **0.0000** | | 0.58 | | 0.0028 | 0.80 | 0.0004 |  |
| 81622 | | UNC93B1 | **1.80** | | | **0.0000** | | 1.55 | | 0.0010 | 1.26 | 0.0003 |  |
| 23032 | | USP33 | **0.70** | | | **0.0004** | | 0.91 | | 0.2267 | 0.89 | 0.0366 |  |
| 10493 | | VAT1 | **1.65** | | | **0.0000** | | 2.07 | | 0.0077 | 1.38 | 0.0000 |  |
| 7409 | | VAV1 | **1.49** | | | **0.0004** | | 1.48 | | 0.0036 | 1.32 | 0.0003 |  |
| 340706 | | VWA2 | **0.70** | | | **0.0004** | | 0.79 | | 0.0121 | 0.90 | 0.0881 |  |
| 54739 | | XAF1 | **1.79** | | | **0.0000** | | 2.30 | | 0.0015 | 1.48 | 0.0013 |  |
| 7498 | | XDH | **1.59** | | | **0.0000** | | 1.73 | | 0.0045 | 1.19 | 0.1311 |  |
| 85463 | | ZC3H12C | **0.67** | | | **0.0006** | | 0.98 | | 0.4334 | 0.89 | 0.1311 |  |
| 340152 | | ZC3H12D | **1.50** | | | **0.0000** | | np | | | 1.16 | 0.1016 |  |
| 64397 | | ZFP106 | **0.71** | | | **0.0000** | | 0.86 | | 0.1341 | 0.94 | 0.1127 |  |
| **B. 536 genes regulated only in NZM2410 30 weeks old vs. 7 wks old.** | | | | | | | | | | | | | |
| Human Entrez Gene ID | | Human Gene symbol | **NZB/W** | | | | | **NZM2410** | | | **NZW/BXSB** | | |
|  |  |  | Fold-change | | | q-value | | Fold-change | | q-value | Fold-change | q-value | |
| 36 | | ACADSB | 0.81 | | | 0.0048 | | **0.69** | | **0.0000** | 0.75 | 0.0000 | |
| 48 | | ACO1 | 0.75 | | | 0.0000 | | **0.69** | | **0.0000** | 0.83 | 0.0000 | |
| 50 | | ACO2 | 0.74 | | | 0.0006 | | **0.67** | | **0.0000** | 0.85 | 0.0000 | |
| 132 | | ADK | 0.73 | | | 0.0031 | | **0.71** | | **0.0007** | 0.83 | 0.0087 | |
| 211 | | ALAS1 | 0.81 | | | 0.0450 | | **0.59** | | **0.0000** | 0.80 | 0.0559 | |
| 216 | | ALDH1A1 | 0.88 | | | 0.1641 | | **1.99** | | **0.0000** | 0.70 | 0.0289 | |
| 219 | | ALDH1B1 | 0.87 | | | 0.0918 | | **0.45** | | **0.0000** | 1.00 | 0.9999 | |
| 223 | | ALDH9A1 | 0.74 | | | 0.0015 | | **0.68** | | **0.0009** | 0.78 | 0.0008 | |
| 307 | | ANXA4 | 1.10 | | | 0.3591 | | **1.61** | | **0.0004** | 1.15 | 0.0065 | |
| 312 | | ANXA13 | 0.93 | | | 0.2051 | | **3.33** | | **0.0004** | 0.86 | 0.0905 | |
| 328 | | APEX1 | 1.25 | | | 0.0011 | | **1.46** | | **0.0000** | 1.30 | 0.0000 | |
| 368 | | ABCC6 | 0.90 | | | 0.0275 | | **0.64** | | **0.0006** | 0.82 | 0.0059 | |
| 488 | | ATP2A2 | 0.87 | | | 0.0048 | | **0.71** | | **0.0000** | 0.81 | 0.0022 | |
| 570 | | BAAT | 0.73 | | | 0.0000 | | **0.49** | | **0.0000** | np | | |
| 593 | | BCKDHA | 0.76 | | | 0.0034 | | **0.71** | | **0.0004** | 0.74 | 0.0000 | |
| 595 | | CCND1 | 0.95 | | | 0.2828 | | **0.62** | | **0.0000** | 0.97 | 0.9999 | |
| 629 | | CFB | 0.98 | | | 0.3334 | | **1.55** | | **0.0003** | 1.26 | 0.0000 | |
| 655 | | BMP7 | 0.75 | | | 0.0006 | | **0.56** | | **0.0000** | 0.82 | 0.0005 | |
| 677 | | ZFP36L1 | 1.29 | | | 0.1172 | | **1.93** | | **0.0000** | 1.04 | 0.3296 | |
| 706 | | TSPO | 1.03 | | | 0.4522 | | **1.53** | | **0.0007** | 1.08 | 0.0078 | |
| 763 | | CA5A | 0.93 | | | 0.2014 | | **0.52** | | **0.0004** | 0.61 | 0.0037 | |
| 810 | | CALML3 | 1.35 | | | 0.0006 | | **2.63** | | **0.0000** | 1.33 | 0.0000 | |
| 826 | | CAPNS1 | 1.19 | | | 0.0131 | | **1.50** | | **0.0006** | 1.22 | 0.0000 | |
| 858 | | CAV2 | 1.21 | | | 0.2163 | | **1.70** | | **0.0009** | 1.34 | 0.0000 | |
| 875 | | CBS | 0.79 | | | 0.0160 | | **0.57** | | **0.0007** | 0.80 | 0.0023 | |
| 928 | | CD9 | 1.33 | | | 0.1227 | | **1.72** | | **0.0007** | 1.33 | 0.0000 | |
| 957 | | ENTPD5 | 0.72 | | | 0.0004 | | **0.70** | | **0.0000** | 0.75 | 0.0000 | |
| 958 | | CD40 | 1.30 | | | 0.0013 | | **1.53** | | **0.0004** | 1.26 | 0.0000 | |
| 961 | | CD47 | 1.38 | | | 0.0000 | | **1.82** | | **0.0006** | 1.30 | 0.0000 | |
| 977 | | CD151 | 1.10 | | | 0.2797 | | **1.42** | | **0.0006** | 1.26 | 0.0000 | |
| 1004 | | CDH6 | 1.00 | | | 0.9999 | | **1.52** | | **0.0004** | 1.17 | 0.0007 | |
| 1026 | | CDKN1A | 1.66 | | | 0.0013 | | **2.05** | | **0.0000** | 1.53 | 0.0031 | |
| 1107 | | CHD3 | 0.80 | | | 0.0023 | | **0.71** | | **0.0004** | 0.89 | 0.1083 | |
| 1185 | | CLCN6 | 0.81 | | | 0.0264 | | **0.62** | | **0.0007** | 0.95 | 0.1870 | |
| 1188 | | CLCNKB | 0.80 | | | 0.0128 | | **0.69** | | **0.0000** | 0.82 | 0.0070 | |
| 1285 | | COL4A3 | 0.78 | | | 0.0055 | | **0.54** | | **0.0000** | 0.80 | 0.0049 | |
| 1286 | | COL4A4 | 0.79 | | | 0.0087 | | **0.58** | | **0.0000** | 0.87 | 0.0254 | |
| 1287 | | COL4A5 | 0.84 | | | 0.0186 | | **0.70** | | **0.0000** | 1.04 | 0.3373 | |
| 1317 | | SLC31A1 | 0.72 | | | 0.0007 | | **0.69** | | **0.0005** | 0.92 | 0.0443 | |
| 1346 | | COX7A1 | 0.65 | | | 0.0041 | | **0.44** | | **0.0005** | 0.71 | 0.0097 | |
| 1491 | | CTH | 0.76 | | | 0.0128 | | **0.69** | | **0.0008** | 0.78 | 0.0000 | |
| 1525 | | CXADR | 1.11 | | | 0.3713 | | **1.72** | | **0.0000** | 1.17 | 0.0302 | |
| 1580 | | CYP4B1 | 0.95 | | | 0.2665 | | **3.57** | | **0.0000** | 0.88 | 0.0079 | |
| 1591 | | CYP24A1 | 1.67 | | | 0.1464 | | **5.92** | | **0.0000** | 2.16 | 0.1898 | |
| 1611 | | DAP | 1.18 | | | 0.1843 | | **1.51** | | **0.0004** | 1.33 | 0.0000 | |
| 1628 | | DBP | 0.58 | | | 0.0029 | | **0.31** | | **0.0007** | 0.35 | 0.0151 | |
| 1636 | | ACE | 0.86 | | | 0.1132 | | **0.60** | | **0.0004** | 0.77 | 0.0141 | |
| 1657 | | DMXL1 | 0.73 | | | 0.0150 | | **0.71** | | **0.0007** | 0.79 | 0.0022 | |
| 1717 | | DHCR7 | 0.73 | | | 0.0000 | | **0.60** | | **0.0004** | 1.03 | 0.3636 | |
| 1719 | | DHFR | 0.78 | | | 0.0077 | | **0.63** | | **0.0000** | 0.80 | 0.0005 | |
| 1727 | | CYB5R3 | 1.09 | | | 0.4035 | | **1.40** | | **0.0009** | 1.23 | 0.0003 | |
| 1743 | | DLST | 0.77 | | | 0.0007 | | **0.69** | | **0.0000** | 0.85 | 0.0000 | |
| 1807 | | DPYS | 0.91 | | | 0.0918 | | **0.54** | | **0.0000** | 0.73 | 0.0047 | |
| 1837 | | DTNA | 1.02 | | | 0.4217 | | **1.44** | | **0.0009** | 0.98 | 0.2561 | |
| 1839 | | HBEGF | 1.22 | | | 0.1342 | | **1.75** | | **0.0008** | 0.95 | 0.9999 | |
| 1910 | | EDNRB | 1.06 | | | 0.4309 | | **0.56** | | **0.0000** | 0.63 | 0.0079 | |
| 1945 | | EFNA4 | 1.09 | | | 0.2828 | | **1.69** | | **0.0000** | 1.24 | 0.0004 | |
| 1952 | | CELSR2 | 0.84 | | | 0.0118 | | **0.61** | | **0.0000** | 0.89 | 0.0112 | |
| 1958 | | EGR1 | 0.85 | | | 0.9999 | | **3.16** | | **0.0006** | 0.63 | 0.2291 | |
| 1969 | | EPHA2 | 1.20 | | | 0.0150 | | **1.54** | | **0.0007** | 1.13 | 0.1016 | |
| 2013 | | EMP2 | 1.30 | | | 0.0029 | | **1.90** | | **0.0000** | 1.39 | 0.0000 | |
| 2021 | | ENDOG | 0.83 | | | 0.0335 | | **0.71** | | **0.0006** | 0.85 | 0.0087 | |
| 2026 | | ENO2 | 1.20 | | | 0.0216 | | **1.57** | | **0.0006** | 1.33 | 0.0029 | |
| 2101 | | ESRRA | 0.79 | | | 0.0000 | | **0.65** | | **0.0000** | 0.83 | 0.0026 | |
| 2103 | | ESRRB | 0.88 | | | 0.0440 | | **0.63** | | **0.0000** | 0.75 | 0.0028 | |
| 2114 | | ETS2 | 1.33 | | | 0.0007 | | **1.49** | | **0.0004** | 1.09 | 0.2291 | |
| 2150 | | F2RL1 | 1.48 | | | 0.0023 | | **2.42** | | **0.0005** | 1.27 | 0.0087 | |
| 2168 | | FABP1 | 1.48 | | | 0.1496 | | **7.55** | | **0.0000** | 0.64 | 0.0857 | |
| 2184 | | FAH | 0.74 | | | 0.0048 | | **0.56** | | **0.0004** | 0.81 | 0.0019 | |
| 2264 | | FGFR4 | 0.78 | | | 0.0023 | | **0.60** | | **0.0000** | 0.87 | 0.0226 | |
| 2273 | | FHL1 | 0.75 | | | 0.0000 | | **0.65** | | **0.0000** | 0.81 | 0.0049 | |
| 2281 | | FKBP1B | 1.19 | | | 0.0055 | | **1.82** | | **0.0004** | 1.13 | 0.2188 | |
| 2303 | | FOXC2 | 0.81 | | | 0.0013 | | **0.69** | | **0.0006** | 0.89 | 0.0698 | |
| 2395 | | FXN | 0.85 | | | 0.0129 | | **0.71** | | **0.0006** | 0.84 | 0.0087 | |
| 2619 | | GAS1 | 0.74 | | | 0.0000 | | **0.64** | | **0.0006** | 0.79 | 0.0106 | |
| 2644 | | GCHFR | 0.78 | | | 0.0173 | | **0.59** | | **0.0004** | 0.85 | 0.0377 | |
| 2706 | | GJB2 | 0.74 | | | 0.0000 | | **0.43** | | **0.0000** | 0.67 | 0.0021 | |
| 2762 | | GMDS | 1.09 | | | 0.2187 | | **1.57** | | **0.0004** | 1.18 | 0.0038 | |
| 2768 | | GNA12 | 0.70 | | | 0.0122 | | **0.61** | | **0.0000** | 0.92 | 0.0944 | |
| 2824 | | GPM6B | 1.40 | | | 0.0000 | | **1.56** | | **0.0004** | 1.35 | 0.0000 | |
| 2892 | | GRIA3 | 0.86 | | | 0.0055 | | **0.63** | | **0.0000** | 1.02 | 0.3749 | |
| 2926 | | GRSF1 | 0.71 | | | 0.0013 | | **0.71** | | **0.0000** | 0.76 | 0.0000 | |
| 2937 | | GSS | 0.81 | | | 0.0186 | | **0.69** | | **0.0000** | 0.83 | 0.0094 | |
| 2946 | | GSTM2 | 0.81 | | | 0.0122 | | **0.56** | | **0.0000** | 0.84 | 0.0357 | |
| 2952 | | GSTT1 | 0.79 | | | 0.0013 | | **1.55** | | **0.0000** | 1.24 | 0.0000 | |
| 2980 | | GUCA2A | 1.23 | | | 0.0216 | | **1.93** | | **0.0006** | 1.20 | 0.0155 | |
| 2990 | | GUSB | 1.38 | | | 0.0000 | | **1.48** | | **0.0007** | 0.92 | 0.9999 | |
| 2995 | | GYPC | 0.89 | | | 0.0505 | | **0.60** | | **0.0000** | 1.01 | 0.9999 | |
| 3081 | | HGD | 0.73 | | | 0.0041 | | **0.58** | | **0.0005** | 0.79 | 0.0003 | |
| 3131 | | HLF | 0.68 | | | 0.0013 | | **0.57** | | **0.0005** | 0.74 | 0.0032 | |
| 3156 | | HMGCR | 0.83 | | | 0.0750 | | **0.40** | | **0.0000** | 0.66 | 0.0135 | |
| 3216 | | HOXB6 | 1.15 | | | 0.2401 | | **1.61** | | **0.0005** | 0.93 | 0.2561 | |
| 3251 | | HPRT1 | 1.14 | | | 0.1008 | | **1.47** | | **0.0004** | 1.16 | 0.0019 | |
| 3275 | | PRMT2 | 1.10 | | | 0.1715 | | **1.54** | | **0.0000** | 1.17 | 0.0097 | |
| 3291 | | HSD11B2 | 0.97 | | | 0.2828 | | **1.47** | | **0.0008** | 1.00 | 0.9999 | |
| 3359 | | HTR3A | 1.15 | | | 0.0223 | | **1.65** | | **0.0004** | 1.14 | 0.0186 | |
| 3418 | | IDH2 | 0.76 | | | 0.0024 | | **0.64** | | **0.0000** | 0.84 | 0.0013 | |
| 3421 | | IDH3G | 0.72 | | | 0.0000 | | **0.70** | | **0.0004** | 0.81 | 0.0000 | |
| 3460 | | IFNGR2 | 1.42 | | | 0.0022 | | **1.80** | | **0.0000** | 1.30 | 0.0003 | |
| 3475 | | IFRD1 | 1.29 | | | 0.0750 | | **1.78** | | **0.0004** | 0.93 | 0.9999 | |
| 3479 | | IGF1 | 0.96 | | | 0.2861 | | **0.64** | | **0.0000** | 1.12 | 0.3052 | |
| 3488 | | IGFBP5 | 0.70 | | | 0.0019 | | **0.45** | | **0.0006** | 0.85 | 0.0571 | |
| 3554 | | IL1R1 | 1.39 | | | 0.0009 | | **1.41** | | **0.0009** | 1.24 | 0.0003 | |
| 3557 | | IL1RN | 1.26 | | | 0.0065 | | **2.00** | | **0.0008** | 1.37 | 0.0003 | |
| 3672 | | ITGA1 | 1.09 | | | 0.2986 | | **1.55** | | **0.0004** | 1.39 | 0.0000 | |
| 3692 | | EIF6 | 1.34 | | | 0.0035 | | **2.10** | | **0.0004** | 1.23 | 0.0008 | |
| 3699 | | ITIH3 | 1.35 | | | 0.0006 | | **2.46** | | **0.0000** | 1.33 | 0.0055 | |
| 3708 | | ITPR1 | 0.74 | | | 0.0000 | | **0.71** | | **0.0000** | 0.85 | 0.0000 | |
| 3709 | | ITPR2 | 0.72 | | | 0.0000 | | **0.68** | | **0.0007** | 0.84 | 0.0000 | |
| 3725 | | JUN | 1.10 | | | 0.4309 | | **2.17** | | **0.0000** | 0.85 | 0.2382 | |
| 3726 | | JUNB | 1.35 | | | 0.1342 | | **2.87** | | **0.0000** | 0.94 | 0.9999 | |
| 3727 | | JUND | 1.12 | | | 0.1192 | | **1.69** | | **0.0000** | 1.16 | 0.0005 | |
| 3764 | | KCNJ8 | 0.91 | | | 0.1531 | | **0.53** | | **0.0009** | 0.83 | 0.0135 | |
| 3784 | | KCNQ1 | 1.21 | | | 0.0150 | | **1.55** | | **0.0000** | 1.11 | 0.1270 | |
| 3815 | | KIT | 1.19 | | | 0.1083 | | **1.52** | | **0.0000** | 0.99 | 0.9999 | |
| 3866 | | KRT15 | 1.13 | | | 0.2751 | | **1.52** | | **0.0006** | 1.17 | 0.0044 | |
| 3976 | | LIF | 1.09 | | | 0.2861 | | **1.46** | | **0.0004** | 1.35 | 0.0003 | |
| 4043 | | LRPAP1 | 0.76 | | | 0.0014 | | **0.55** | | **0.0000** | 0.85 | 0.0053 | |
| 4118 | | MAL | 1.03 | | | 0.9999 | | **1.48** | | **0.0005** | 1.18 | 0.0060 | |
| 4199 | | ME1 | 0.76 | | | 0.0024 | | **0.57** | | **0.0004** | 0.73 | 0.0023 | |
| 4493 | | MT1E | 1.75 | | | 0.0106 | | **2.04** | | **0.0005** | 1.64 | 0.0038 | |
| 4594 | | MUT | 0.71 | | | 0.0017 | | **0.64** | | **0.0000** | 0.78 | 0.0000 | |
| 4597 | | MVD | 0.75 | | | 0.0007 | | **0.56** | | **0.0000** | 0.88 | 0.0817 | |
| 4609 | | MYC | 1.72 | | | 0.0015 | | **2.50** | | **0.0006** | 1.50 | 0.0013 | |
| 4616 | | GADD45B | 1.28 | | | 0.0023 | | **1.49** | | **0.0005** | 0.99 | 0.9999 | |
| 4644 | | MYO5A | 0.61 | | | 0.0013 | | **0.33** | | **0.0000** | 0.64 | 0.0325 | |
| 4708 | | NDUFB2 | 0.77 | | | 0.0004 | | **0.71** | | **0.0000** | 0.95 | 0.1721 | |
| 4751 | | NEK2 | 1.32 | | | 0.0024 | | **1.42** | | **0.0004** | 1.44 | 0.0022 | |
| 4780 | | NFE2L2 | 1.14 | | | 0.1740 | | **1.69** | | **0.0000** | 1.15 | 0.0817 | |
| 4790 | | NFKB1 | 1.27 | | | 0.0017 | | **1.43** | | **0.0000** | 1.34 | 0.0000 | |
| 4791 | | NFKB2 | 1.37 | | | 0.0000 | | **1.52** | | **0.0000** | 1.32 | 0.0000 | |
| 4803 | | NGF | 1.40 | | | 0.0097 | | **2.16** | | **0.0000** | 1.14 | 0.0013 | |
| 4855 | | NOTCH4 | 1.06 | | | 0.4019 | | **0.70** | | **0.0004** | 0.82 | 0.0021 | |
| 4860 | | PNP | 1.19 | | | 0.1772 | | **1.44** | | **0.0004** | 1.10 | 0.0751 | |
| 4868 | | NPHS1 | 0.92 | | | 0.1843 | | **0.64** | | **0.0003** | 0.86 | 0.0302 | |
| 4881 | | NPR1 | 1.16 | | | 0.1377 | | **0.67** | | **0.0005** | 0.80 | 0.0000 | |
| 4883 | | NPR3 | 0.87 | | | 0.0013 | | **0.64** | | **0.0009** | 0.79 | 0.0005 | |
| 4893 | | NRAS | 1.26 | | | 0.0015 | | **1.44** | | **0.0004** | 1.24 | 0.0000 | |
| 4908 | | NTF3 | 0.83 | | | 0.0102 | | **0.64** | | **0.0004** | 0.91 | 0.0298 | |
| 5019 | | OXCT1 | 0.67 | | | 0.0018 | | **0.57** | | **0.0000** | 0.79 | 0.0016 | |
| 5033 | | P4HA1 | 0.74 | | | 0.0000 | | **0.66** | | **0.0004** | 0.69 | 0.0033 | |
| 5046 | | PCSK6 | 0.83 | | | 0.0008 | | **0.63** | | **0.0008** | 0.78 | 0.0000 | |
| 5100 | | PCDH8 | np | | | | | **7.15** | | **0.0000** | 1.37 | 0.0041 | |
| 5129 | | CDK18 | 0.86 | | | 0.0593 | | **0.69** | | **0.0000** | 0.95 | 0.2188 | |
| 5140 | | PDE3B | 1.28 | | | 0.0075 | | **1.98** | | **0.0000** | 1.30 | 0.0009 | |
| 5165 | | PDK3 | 0.85 | | | 0.0290 | | **0.66** | | **0.0000** | 0.78 | 0.0095 | |
| 5168 | | ENPP2 | 0.75 | | | 0.0055 | | **0.60** | | **0.0008** | 0.75 | 0.0007 | |
| 5192 | | PEX10 | 0.83 | | | 0.0004 | | **0.68** | | **0.0004** | 0.82 | 0.0043 | |
| 5213 | | PFKM | 0.75 | | | 0.0000 | | **0.69** | | **0.0006** | 0.80 | 0.0000 | |
| 5245 | | PHB | 0.78 | | | 0.0015 | | **0.71** | | **0.0000** | 0.86 | 0.0000 | |
| 5351 | | PLOD1 | 1.03 | | | 0.4111 | | **0.71** | | **0.0000** | 0.83 | 0.0008 | |
| 5498 | | PPOX | 0.93 | | | 0.1947 | | **0.70** | | **0.0006** | 0.96 | 0.2090 | |
| 5533 | | PPP3CC | 0.95 | | | 0.2861 | | **0.67** | | **0.0005** | 0.77 | 0.0047 | |
| 5564 | | PRKAB1 | 1.20 | | | 0.1675 | | **1.41** | | **0.0005** | 1.22 | 0.0033 | |
| 5652 | | PRSS8 | 0.73 | | | 0.0015 | | **0.62** | | **0.0000** | 0.97 | 0.2525 | |
| 5819 | | PVRL2 | 1.34 | | | 0.0004 | | **1.83** | | **0.0004** | 1.17 | 0.0058 | |
| 5824 | | PEX19 | 0.78 | | | 0.0000 | | **0.68** | | **0.0000** | 0.83 | 0.0000 | |
| 5873 | | RAB27A | 0.87 | | | 0.0531 | | **0.69** | | **0.0009** | 0.84 | 0.0005 | |
| 5912 | | RAP2B | 1.24 | | | 0.0007 | | **1.45** | | **0.0000** | 1.12 | 0.0004 | |
| 5931 | | RBBP7 | 1.07 | | | 0.4176 | | **1.43** | | **0.0007** | 1.18 | 0.0011 | |
| 5986 | | RFNG | 0.86 | | | 0.0023 | | **0.66** | | **0.0000** | 0.93 | 0.1127 | |
| 6050 | | RNH1 | 1.13 | | | 0.2293 | | **1.44** | | **0.0004** | 1.28 | 0.0000 | |
| 6354 | | CCL7 | 1.54 | | | 0.0023 | | **1.81** | | **0.0005** | 1.74 | 0.0022 | |
| 6364 | | CCL20 | 1.26 | | | 0.0041 | | **1.67** | | **0.0007** | 1.37 | 0.0186 | |
| 6391 | | SDHC | 0.73 | | | 0.0004 | | **0.71** | | **0.0005** | 0.90 | 0.0007 | |
| 6415 | | SEPW1 | 1.29 | | | 0.0004 | | **1.57** | | **0.0000** | 1.32 | 0.0000 | |
| 6450 | | SH3BGR | 0.78 | | | 0.0000 | | **0.46** | | **0.0000** | 1.01 | 0.9999 | |
| 6532 | | SLC6A4 | 0.73 | | | 0.0000 | | **0.71** | | **0.0007** | 0.77 | 0.0226 | |
| 6539 | | SLC6A12 | 0.89 | | | 0.2460 | | **0.47** | | **0.0005** | 0.82 | 0.0310 | |
| 6540 | | SLC6A13 | 0.93 | | | 0.1947 | | **0.58** | | **0.0000** | 0.85 | 0.0124 | |
| 6561 | | SLC13A1 | 1.16 | | | 0.2187 | | **1.86** | | **0.0000** | 1.33 | 0.0007 | |
| 6563 | | SLC14A1 | 0.77 | | | 0.0000 | | **0.49** | | **0.0000** | 0.72 | 0.0003 | |
| 6567 | | SLC16A2 | 0.78 | | | 0.0029 | | **0.64** | | **0.0004** | 0.87 | 0.0428 | |
| 6574 | | SLC20A1 | 1.39 | | | 0.0000 | | **2.10** | | **0.0005** | 1.11 | 0.2153 | |
| 6595 | | SMARCA2 | 0.76 | | | 0.0000 | | **0.71** | | **0.0000** | 0.75 | 0.0484 | |
| 6649 | | SOD3 | 0.80 | | | 0.0250 | | **0.59** | | **0.0005** | 0.94 | 0.1747 | |
| 6832 | | SUPV3L1 | 0.79 | | | 0.0000 | | **0.68** | | **0.0000** | 0.90 | 0.0474 | |
| 6875 | | TAF4B | 1.12 | | | 0.0258 | | **1.48** | | **0.0005** | 1.03 | 0.3467 | |
| 7048 | | TGFBR2 | 1.29 | | | 0.0106 | | **1.75** | | **0.0000** | 1.21 | 0.0003 | |
| 7050 | | TGIF1 | 1.49 | | | 0.0521 | | **2.13** | | **0.0000** | 1.26 | 0.0065 | |
| 7071 | | KLF10 | 1.07 | | | 0.4176 | | **2.06** | | **0.0008** | 1.13 | 0.1311 | |
| 7122 | | CLDN5 | 0.93 | | | 0.0781 | | **0.70** | | **0.0006** | 0.87 | 0.0022 | |
| 7263 | | TST | 0.71 | | | 0.0013 | | **0.41** | | **0.0000** | 0.83 | 0.0406 | |
| 7274 | | TTPA | 1.15 | | | 0.4176 | | **1.80** | | **0.0004** | 1.26 | 0.1016 | |
| 7280 | | TUBB2A | 1.28 | | | 0.2051 | | **2.14** | | **0.0000** | 1.58 | 0.0039 | |
| 7286 | | TUFT1 | 1.16 | | | 0.2861 | | **1.57** | | **0.0004** | 0.78 | 0.0009 | |
| 7366 | | UGT2B15 | 1.88 | | | 0.0807 | | **6.62** | | **0.0007** | 1.09 | 0.9999 | |
| 7378 | | UPP1 | 1.23 | | | 0.0131 | | **1.45** | | **0.0000** | 1.05 | 0.2798 | |
| 7414 | | VCL | 1.21 | | | 0.1008 | | **1.53** | | **0.0004** | 1.34 | 0.0000 | |
| 7436 | | VLDLR | 0.70 | | | 0.0039 | | **0.68** | | **0.0000** | 1.08 | 0.2188 | |
| 7490 | | WT1 | 0.89 | | | 0.0727 | | **0.68** | | **0.0000** | 0.93 | 0.1683 | |
| 7494 | | XBP1 | 1.33 | | | 0.0013 | | **1.70** | | **0.0000** | 1.11 | 0.1721 | |
| 7512 | | XPNPEP2 | 0.95 | | | 0.2094 | | **0.65** | | **0.0006** | 0.94 | 0.0681 | |
| 7534 | | YWHAZ | 1.17 | | | 0.0450 | | **1.47** | | **0.0006** | 1.02 | 0.3749 | |
| 7867 | | MAPKAPK3 | 1.15 | | | 0.0389 | | **1.56** | | **0.0009** | 1.27 | 0.0000 | |
| 7869 | | SEMA3B | 0.73 | | | 0.0000 | | **0.65** | | **0.0000** | 0.89 | 0.0064 | |
| 8321 | | FZD1 | 0.80 | | | 0.0048 | | **0.68** | | **0.0000** | 0.96 | 0.2353 | |
| 8324 | | FZD7 | 1.15 | | | 0.0837 | | **1.42** | | **0.0008** | 1.06 | 0.2588 | |
| 8434 | | RECK | 0.74 | | | 0.0000 | | **0.63** | | **0.0006** | 0.97 | 0.2124 | |
| 8437 | | RASAL1 | 1.08 | | | 0.1843 | | **1.49** | | **0.0009** | 0.95 | 0.1358 | |
| 8470 | | SORBS2 | 1.31 | | | 0.0341 | | **1.82** | | **0.0006** | 1.38 | 0.0000 | |
| 8508 | | NIPSNAP1 | 0.72 | | | 0.0004 | | **0.70** | | **0.0009** | 0.76 | 0.0000 | |
| 8521 | | GCM1 | 0.97 | | | 0.3298 | | **0.70** | | **0.0006** | 0.90 | 0.0399 | |
| 8540 | | AGPS | 1.04 | | | 0.9999 | | **0.69** | | **0.0000** | 0.62 | 0.0053 | |
| 8574 | | AKR7A2 | 0.76 | | | 0.0013 | | **0.65** | | **0.0000** | 0.84 | 0.0004 | |
| 8612 | | PPAP2C | 1.17 | | | 0.0976 | | **1.61** | | **0.0000** | 1.26 | 0.0000 | |
| 8633 | | UNC5C | 0.90 | | | 0.0258 | | **0.64** | | **0.0007** | 0.96 | 0.1626 | |
| 8789 | | FBP2 | 1.12 | | | 0.3334 | | **2.00** | | **0.0005** | 1.49 | 0.0406 | |
| 8819 | | SAP30 | 0.72 | | | 0.0087 | | **0.51** | | **0.0000** | 0.76 | 0.0213 | |
| 8825 | | LIN7A | 0.76 | | | 0.0004 | | **0.54** | | **0.0008** | 0.88 | 0.0053 | |
| 8829 | | NRP1 | 1.13 | | | 0.1906 | | **1.46** | | **0.0004** | 1.05 | 0.2413 | |
| 8863 | | PER3 | 0.78 | | | 0.0000 | | **0.52** | | **0.0000** | 0.76 | 0.0083 | |
| 8915 | | BCL10 | 1.18 | | | 0.0660 | | **1.44** | | **0.0000** | 1.11 | 0.0037 | |
| 8935 | | SKAP2 | 1.23 | | | 0.0000 | | **1.51** | | **0.0000** | 1.25 | 0.0000 | |
| 8991 | | SELENBP1 | 0.82 | | | 0.0505 | | **0.54** | | **0.0000** | 0.74 | 0.0000 | |
| 9022 | | CLIC3 | 0.81 | | | 0.0335 | | **0.48** | | **0.0000** | 0.77 | 0.0023 | |
| 9027 | | NAT8 | 0.68 | | | 0.0075 | | **0.57** | | **0.0009** | 0.86 | 0.0226 | |
| 9052 | | GPRC5A | 1.19 | | | 0.0405 | | **1.73** | | **0.0005** | 1.15 | 0.0710 | |
| 9172 | | MYOM2 | 0.70 | | | 0.0055 | | **0.53** | | **0.0008** | 0.78 | 0.0000 | |
| 9249 | | DHRS3 | 0.86 | | | 0.0492 | | **0.61** | | **0.0000** | 0.94 | 0.2291 | |
| 9254 | | CACNA2D2 | 0.77 | | | 0.0000 | | **0.59** | | **0.0000** | 0.83 | 0.0011 | |
| 9380 | | GRHPR | 0.77 | | | 0.0007 | | **0.68** | | **0.0004** | 0.81 | 0.0003 | |
| 9414 | | TJP2 | 1.11 | | | 0.2579 | | **1.59** | | **0.0004** | 1.25 | 0.0000 | |
| 9452 | | ITM2A | 0.85 | | | 0.0100 | | **0.61** | | **0.0004** | 0.87 | 0.0064 | |
| 9467 | | SH3BP5 | 0.84 | | | 0.0055 | | **0.67** | | **0.0005** | 0.72 | 0.0000 | |
| 9532 | | BAG2 | 1.43 | | | 0.1437 | | **2.24** | | **0.0006** | 1.18 | 0.1110 | |
| 9545 | | RAB3D | 1.25 | | | 0.0004 | | **1.73** | | **0.0000** | 1.24 | 0.0003 | |
| 9630 | | GNA14 | 0.86 | | | 0.0232 | | **0.59** | | **0.0004** | 0.79 | 0.0377 | |
| 9696 | | CROCC | 0.76 | | | 0.0024 | | **0.64** | | **0.0000** | 0.95 | 0.1988 | |
| 9704 | | DHX34 | 0.86 | | | 0.0065 | | **0.61** | | **0.0000** | 0.87 | 0.0112 | |
| 9762 | | ProSAPiP1 | 0.81 | | | 0.0068 | | **0.70** | | **0.0000** | 1.01 | 0.9999 | |
| 9812 | | KIAA0141 | 0.85 | | | 0.0060 | | **0.70** | | **0.0005** | 0.79 | 0.0003 | |
| 9829 | | DNAJC6 | 0.76 | | | 0.0000 | | **0.54** | | **0.0005** | 0.87 | 0.1270 | |
| 9900 | | SV2A | 1.08 | | | 0.2401 | | **0.70** | | **0.0005** | 0.91 | 0.0710 | |
| 9942 | | XYLB | 0.76 | | | 0.0097 | | **0.56** | | **0.0000** | 0.98 | 0.9999 | |
| 9971 | | NR1H4 | 0.74 | | | 0.0044 | | **0.70** | | **0.0006** | 0.80 | 0.0496 | |
| 9991 | | PTBP3 | 1.26 | | | 0.1641 | | **1.89** | | **0.0006** | 1.14 | 0.3090 | |
| 10058 | | ABCB6 | 0.88 | | | 0.0900 | | **0.67** | | **0.0000** | 0.92 | 0.0857 | |
| 10097 | | ACTR2 | 1.12 | | | 0.1976 | | **1.41** | | **0.0000** | 1.15 | 0.0000 | |
| 10105 | | PPIF | 0.72 | | | 0.0000 | | **0.68** | | **0.0005** | 0.95 | 0.1065 | |
| 10109 | | ARPC2 | 1.24 | | | 0.0122 | | **1.62** | | **0.0004** | 1.23 | 0.0000 | |
| 10209 | | EIF1 | 1.09 | | | 0.4050 | | **1.52** | | **0.0004** | 1.12 | 0.1311 | |
| 10240 | | MRPS31 | 0.75 | | | 0.0003 | | **0.69** | | **0.0004** | 0.81 | 0.0000 | |
| 10318 | | TNIP1 | 1.38 | | | 0.0000 | | **1.62** | | **0.0007** | 1.27 | 0.0078 | |
| 10488 | | CREB3 | 1.06 | | | 0.2579 | | **1.43** | | **0.0008** | 1.21 | 0.0000 | |
| 10585 | | POMT1 | 0.79 | | | 0.0006 | | **0.71** | | **0.0004** | 0.81 | 0.0009 | |
| 10615 | | SPAG5 | 0.91 | | | 0.0837 | | **0.61** | | **0.0000** | 1.07 | 0.2499 | |
| 10626 | | TRIM16 | 1.02 | | | 0.9999 | | **1.44** | | **0.0004** | 1.12 | 0.0571 | |
| 10627 | | MYL12A | 1.10 | | | 0.1906 | | **1.48** | | **0.0000** | 1.23 | 0.0000 | |
| 10628 | | TXNIP | 0.92 | | | 0.2460 | | **1.61** | | **0.0008** | 1.04 | 0.3698 | |
| 10653 | | SPINT2 | 1.06 | | | 0.4138 | | **1.47** | | **0.0008** | 1.17 | 0.0000 | |
| 10661 | | KLF1 | 1.02 | | | 0.9999 | | **0.70** | | **0.0004** | 0.84 | 0.0857 | |
| 10699 | | CORIN | np | | | | | **0.57** | | **0.0004** | 0.78 | 0.0005 | |
| 10783 | | NEK6 | 1.22 | | | 0.0024 | | **1.68** | | **0.0000** | 1.26 | 0.0004 | |
| 10865 | | ARID5A | 1.30 | | | 0.0071 | | **1.50** | | **0.0000** | 1.26 | 0.0016 | |
| 10873 | | ME3 | 0.80 | | | 0.0035 | | **0.68** | | **0.0000** | 0.75 | 0.0000 | |
| 10935 | | PRDX3 | 0.81 | | | 0.0011 | | **0.71** | | **0.0000** | 0.86 | 0.0000 | |
| 10955 | | SERINC3 | 1.24 | | | 0.0240 | | **1.46** | | **0.0004** | 1.05 | 0.3296 | |
| 10957 | | PNRC1 | 1.15 | | | 0.1265 | | **1.65** | | **0.0000** | 1.27 | 0.0226 | |
| 10981 | | RAB32 | 1.18 | | | 0.0855 | | **1.48** | | **0.0005** | 1.27 | 0.0003 | |
| 11107 | | PRDM5 | 0.75 | | | 0.0000 | | **0.61** | | **0.0000** | 0.80 | 0.0003 | |
| 11112 | | HIBADH | 0.73 | | | 0.0013 | | **0.64** | | **0.0003** | 0.77 | 0.0000 | |
| 11237 | | RNF24 | 0.72 | | | 0.0000 | | **0.35** | | **0.0000** | 0.61 | 0.0046 | |
| 11332 | | ACOT7 | 0.70 | | | 0.0041 | | **0.56** | | **0.0004** | 1.03 | 0.3281 | |
| 22996 | | TTC39A | 1.15 | | | 0.0216 | | **1.75** | | **0.0004** | 1.31 | 0.0000 | |
| 23037 | | PDZD2 | 0.84 | | | 0.0000 | | **0.58** | | **0.0000** | 0.83 | 0.0003 | |
| 23082 | | PPRC1 | 1.31 | | | 0.0024 | | **1.40** | | **0.0007** | 1.03 | 0.3549 | |
| 23109 | | DDN | 0.76 | | | 0.0004 | | **0.52** | | **0.0000** | 0.80 | 0.0289 | |
| 23179 | | RGL1 | 0.73 | | | 0.0006 | | **0.64** | | **0.0008** | 0.88 | 0.0097 | |
| 23220 | | DTX4 | 1.48 | | | 0.0065 | | **1.49** | | **0.0000** | 1.37 | 0.0011 | |
| 23302 | | WSCD1 | 0.83 | | | 0.0060 | | **0.50** | | **0.0000** | 0.80 | 0.0000 | |
| 23348 | | DOCK9 | 0.73 | | | 0.0013 | | **0.68** | | **0.0004** | 0.92 | 0.0638 | |
| 23355 | | VPS8 | 0.85 | | | 0.0048 | | **0.65** | | **0.0000** | 0.84 | 0.1898 | |
| 23371 | | TENC1 | 1.08 | | | 0.4252 | | **0.71** | | **0.0000** | 0.80 | 0.0165 | |
| 23452 | | ANGPTL2 | 0.75 | | | 0.0027 | | **0.48** | | **0.0004** | 0.94 | 0.2064 | |
| 23483 | | TGDS | 0.78 | | | 0.0017 | | **0.71** | | **0.0005** | 0.79 | 0.0005 | |
| 23517 | | SKIV2L2 | 0.78 | | | 0.0057 | | **0.71** | | **0.0004** | 0.89 | 0.1127 | |
| 23529 | | CLCF1 | 1.26 | | | 0.0560 | | **2.14** | | **0.0000** | 1.25 | 0.0029 | |
| 23590 | | PDSS1 | 0.75 | | | 0.0048 | | **0.50** | | **0.0000** | 0.76 | 0.0041 | |
| 23607 | | CD2AP | 1.20 | | | 0.2187 | | **2.08** | | **0.0006** | 1.39 | 0.0000 | |
| 23624 | | CBLC | 1.15 | | | 0.1531 | | **1.47** | | **0.0000** | 1.05 | 0.1000 | |
| 23630 | | KCNE1L | 0.74 | | | 0.0000 | | **0.35** | | **0.0000** | 1.03 | 0.3403 | |
| 23677 | | SH3BP4 | 1.14 | | | 0.0949 | | **1.46** | | **0.0005** | 1.10 | 0.0109 | |
| 23767 | | FLRT3 | np | | | | | **0.66** | | **0.0004** | 1.00 | 0.9999 | |
| 24147 | | FJX1 | 1.10 | | | 0.0692 | | **1.71** | | **0.0000** | 1.21 | 0.0019 | |
| 25758 | | C11orf41 | 1.04 | | | 0.4194 | | **1.83** | | **0.0009** | 1.12 | 0.0176 | |
| 25805 | | BAMBI | 0.78 | | | 0.0013 | | **0.55** | | **0.0000** | 0.86 | 0.0070 | |
| 25821 | | MTO1 | 0.79 | | | 0.0007 | | **0.67** | | **0.0000** | 0.83 | 0.0064 | |
| 25897 | | RNF19A | 1.21 | | | 0.0459 | | **1.52** | | **0.0006** | 1.20 | 0.0030 | |
| 25953 | | PNKD | 0.87 | | | 0.0880 | | **0.62** | | **0.0000** | 0.73 | 0.0006 | |
| 26001 | | RNF167 | 0.78 | | | 0.0007 | | **0.70** | | **0.0000** | 0.79 | 0.0000 | |
| 26030 | | PLEKHG3 | 0.83 | | | 0.0060 | | **0.70** | | **0.0000** | 0.92 | 0.0165 | |
| 26061 | | HACL1 | 0.77 | | | 0.0145 | | **0.65** | | **0.0009** | 0.76 | 0.1402 | |
| 26073 | | POLDIP2 | 0.80 | | | 0.0006 | | **0.67** | | **0.0000** | 0.92 | 0.0089 | |
| 26115 | | TANC2 | 0.72 | | | 0.0007 | | **0.71** | | **0.0005** | 1.04 | 0.3202 | |
| 26118 | | WSB1 | 1.39 | | | 0.0024 | | **2.39** | | **0.0000** | 0.93 | 0.9999 | |
| 26270 | | FBXO6 | 1.31 | | | 0.0080 | | **1.52** | | **0.0009** | 1.28 | 0.0000 | |
| 26502 | | NARF | 0.84 | | | 0.0006 | | **0.58** | | **0.0000** | 0.84 | 0.0055 | |
| 26578 | | OSTF1 | 1.13 | | | 0.3928 | | **1.47** | | **0.0004** | 1.20 | 0.0032 | |
| 27034 | | ACAD8 | 0.72 | | | 0.0000 | | **0.68** | | **0.0006** | 0.75 | 0.0000 | |
| 27071 | | DAPP1 | 1.28 | | | 0.0000 | | **1.98** | | **0.0005** | 1.35 | 0.0000 | |
| 27123 | | DKK2 | 1.02 | | | 0.9999 | | **1.55** | | **0.0005** | 1.11 | 0.2771 | |
| 27131 | | SNX5 | 1.08 | | | 0.3756 | | **1.42** | | **0.0004** | 0.95 | 0.2017 | |
| 27141 | | CIDEB | 0.73 | | | 0.0000 | | **0.65** | | **0.0005** | 0.87 | 0.0273 | |
| 27289 | | RND1 | 1.25 | | | 0.0179 | | **2.40** | | **0.0000** | 1.09 | 0.1142 | |
| 27306 | | HPGDS | 1.28 | | | 0.0029 | | **1.68** | | **0.0009** | 1.21 | 0.0009 | |
| 28232 | | SLCO3A1 | 1.26 | | | 0.0024 | | **1.65** | | **0.0006** | 0.84 | 0.0129 | |
| 28959 | | TMEM176B | 1.11 | | | 0.2187 | | **1.54** | | **0.0000** | 1.19 | 0.0129 | |
| 28971 | | C11orf67 | 0.73 | | | 0.0100 | | **0.64** | | **0.0004** | 0.73 | 0.0000 | |
| 28982 | | FLVCR1 | 1.67 | | | 0.0013 | | **2.48** | | **0.0000** | 1.20 | 0.1808 | |
| 28999 | | KLF15 | 0.78 | | | 0.0389 | | **0.50** | | **0.0000** | 0.89 | 0.0801 | |
| 29088 | | MRPL15 | 0.73 | | | 0.0000 | | **0.68** | | **0.0006** | 0.88 | 0.0070 | |
| 29914 | | UBIAD1 | 0.80 | | | 0.0006 | | **0.62** | | **0.0000** | 0.78 | 0.0060 | |
| 29984 | | RHOD | 1.25 | | | 0.0368 | | **1.49** | | **0.0000** | 1.21 | 0.0089 | |
| 30061 | | SLC40A1 | 1.14 | | | 0.3083 | | **1.48** | | **0.0000** | 1.32 | 0.0005 | |
| 50853 | | VILL | 0.88 | | | 0.1035 | | **0.61** | | **0.0000** | 0.72 | 0.0024 | |
| 51002 | | TPRKB | 0.81 | | | 0.0080 | | **0.61** | | **0.0000** | 0.81 | 0.0049 | |
| 51011 | | FAHD2A | 0.77 | | | 0.0004 | | **0.68** | | **0.0000** | 0.84 | 0.0011 | |
| 51067 | | YARS2 | 0.74 | | | 0.0006 | | **0.67** | | **0.0000** | 0.89 | 0.0496 | |
| 51071 | | DERA | 0.77 | | | 0.0004 | | **0.71** | | **0.0000** | 0.99 | 0.9999 | |
| 51115 | | FAM82B | 0.87 | | | 0.0014 | | **0.69** | | **0.0004** | 0.87 | 0.0014 | |
| 51149 | | C5orf45 | 0.81 | | | 0.0006 | | **0.71** | | **0.0004** | 0.74 | 0.0022 | |
| 51188 | | SS18L2 | 0.77 | | | 0.0118 | | **0.69** | | **0.0004** | 0.87 | 0.0009 | |
| 51205 | | ACP6 | 0.75 | | | 0.0006 | | **0.66** | | **0.0000** | 0.79 | 0.0000 | |
| 51295 | | ECSIT | 0.80 | | | 0.0007 | | **0.71** | | **0.0004** | 0.86 | 0.0011 | |
| 51363 | | CHST15 | 0.98 | | | 0.3928 | | **0.57** | | **0.0000** | 0.85 | 0.0608 | |
| 51364 | | ZMYND10 | 0.81 | | | 0.0290 | | **0.51** | | **0.0000** | 0.84 | 0.0124 | |
| 51393 | | TRPV2 | 1.33 | | | 0.0007 | | **1.52** | | **0.0004** | 1.01 | 0.9999 | |
| 51409 | | HEMK1 | 0.81 | | | 0.0007 | | **0.66** | | **0.0000** | 0.87 | 0.0032 | |
| 51458 | | RHCG | 0.78 | | | 0.0029 | | **0.69** | | **0.0000** | 0.73 | 0.0000 | |
| 51475 | | CABP2 | 0.95 | | | 0.1871 | | **0.64** | | **0.0006** | 0.88 | 0.0060 | |
| 51560 | | RAB6B | 1.11 | | | 0.0949 | | **1.80** | | **0.0000** | 1.13 | 0.2648 | |
| 51599 | | LSR | 1.22 | | | 0.1227 | | **1.66** | | **0.0007** | 1.19 | 0.0011 | |
| 51703 | | ACSL5 | 1.33 | | | 0.0008 | | **1.52** | | **0.0006** | 1.37 | 0.0000 | |
| 53340 | | SPA17 | 0.79 | | | 0.0275 | | **0.62** | | **0.0000** | 0.84 | 0.0484 | |
| 54148 | | MRPL39 | 0.73 | | | 0.0006 | | **0.69** | | **0.0005** | 0.81 | 0.0069 | |
| 54205 | | CYCS | 0.86 | | | 0.0138 | | **0.64** | | **0.0004** | 0.75 | 0.0009 | |
| 54467 | | ANKIB1 | 0.78 | | | 0.0004 | | **0.68** | | **0.0005** | 0.92 | 0.0474 | |
| 54494 | | C11orf71 | 0.82 | | | 0.0044 | | **0.66** | | **0.0000** | 0.73 | 0.0000 | |
| 54498 | | SMOX | 1.33 | | | 0.0080 | | **2.27** | | **0.0004** | 1.35 | 0.0000 | |
| 54507 | | ADAMTSL4 | 1.19 | | | 0.0807 | | **1.41** | | **0.0007** | 1.30 | 0.0019 | |
| 54741 | | LEPROT | 1.25 | | | 0.0004 | | **1.42** | | **0.0004** | 1.27 | 0.0000 | |
| 54829 | | ASPN | 0.88 | | | 0.0727 | | **0.51** | | **0.0004** | 0.99 | 0.9999 | |
| 54845 | | ESRP1 | 1.22 | | | 0.2619 | | **1.68** | | **0.0006** | 1.08 | 0.2735 | |
| 54858 | | PGPEP1 | 0.75 | | | 0.0000 | | **0.68** | | **0.0000** | 0.79 | 0.0406 | |
| 54898 | | ELOVL2 | 0.71 | | | 0.0083 | | **0.68** | | **0.0004** | 1.18 | 0.0502 | |
| 54964 | | C1orf56 | 0.91 | | | 0.0405 | | **0.65** | | **0.0000** | 0.97 | 0.2703 | |
| 55002 | | TMCO3 | 0.73 | | | 0.0000 | | **0.61** | | **0.0000** | 0.76 | 0.0213 | |
| 55068 | | ENOX1 | np | | | | | **2.00** | | **0.0000** | 1.37 | 0.0013 | |
| 55101 | | ATP5SL | 0.75 | | | 0.0000 | | **0.65** | | **0.0006** | 0.80 | 0.0000 | |
| 55191 | | NADSYN1 | 0.76 | | | 0.0000 | | **0.69** | | **0.0004** | 0.78 | 0.0000 | |
| 55260 | | TMEM143 | 0.81 | | | 0.0008 | | **0.65** | | **0.0000** | 0.73 | 0.0000 | |
| 55268 | | ECHDC2 | 0.82 | | | 0.0199 | | **0.65** | | **0.0000** | 0.77 | 0.0000 | |
| 55311 | | ZNF444 | 0.88 | | | 0.0560 | | **0.71** | | **0.0000** | 0.79 | 0.0013 | |
| 55335 | | NIPSNAP3B | 1.17 | | | 0.0855 | | **1.46** | | **0.0004** | 1.23 | 0.0066 | |
| 55365 | | TMEM176A | 1.19 | | | 0.0207 | | **1.92** | | **0.0000** | 1.22 | 0.0022 | |
| 55572 | | FOXRED1 | 0.84 | | | 0.0008 | | **0.68** | | **0.0000** | 0.84 | 0.0000 | |
| 55757 | | UGGT2 | np | | | | | **0.68** | | **0.0000** | 0.88 | 0.0213 | |
| 55764 | | IFT122 | 0.78 | | | 0.0023 | | **0.67** | | **0.0000** | 0.77 | 0.0129 | |
| 55840 | | EAF2 | 0.89 | | | 0.1172 | | **0.62** | | **0.0000** | 0.96 | 0.2325 | |
| 55847 | | CISD1 | 0.74 | | | 0.0015 | | **0.70** | | **0.0008** | 0.82 | 0.0000 | |
| 55852 | | TEX2 | 0.74 | | | 0.0004 | | **0.69** | | **0.0005** | 0.82 | 0.0000 | |
| 55856 | | ACOT13 | 0.71 | | | 0.0015 | | **0.63** | | **0.0005** | 0.99 | 0.9999 | |
| 55890 | | GPRC5C | 0.71 | | | 0.0013 | | **0.65** | | **0.0004** | 0.76 | 0.0003 | |
| 55959 | | SULF2 | 1.33 | | | 0.0006 | | **1.52** | | **0.0009** | 1.31 | 0.0003 | |
| 56204 | | FAM214A | 0.78 | | | 0.0017 | | **0.70** | | **0.0000** | 0.86 | 0.0710 | |
| 56267 | | CCBL2 | 0.81 | | | 0.0352 | | **0.63** | | **0.0005** | 0.81 | 0.0078 | |
| 56475 | | RPRM | 1.35 | | | 0.0750 | | **2.23** | | **0.0009** | 1.27 | 0.0003 | |
| 56670 | | SUCNR1 | 0.57 | | | 0.0013 | | **0.45** | | **0.0000** | 0.87 | 0.1292 | |
| 56829 | | ZC3HAV1 | 1.23 | | | 0.0015 | | **1.77** | | **0.0004** | 1.26 | 0.0000 | |
| 56905 | | C15orf39 | 1.25 | | | 0.0048 | | **1.49** | | **0.0003** | 1.16 | 0.0388 | |
| 56942 | | CMC2 | 0.91 | | | 0.0593 | | **0.70** | | **0.0000** | 0.95 | 0.1142 | |
| 56990 | | CDC42SE2 | 1.39 | | | 0.0000 | | **1.57** | | **0.0007** | 1.23 | 0.0044 | |
| 57128 | | LYRM4 | 0.90 | | | 0.0900 | | **0.70** | | **0.0000** | 0.86 | 0.0399 | |
| 57157 | | PHTF2 | 0.83 | | | 0.0118 | | **0.69** | | **0.0007** | 0.88 | 0.0298 | |
| 57211 | | GPR126 | 1.28 | | | 0.0000 | | **1.81** | | **0.0005** | 1.27 | 0.0000 | |
| 57508 | | INTS2 | 0.74 | | | 0.0023 | | **0.71** | | **0.0008** | 0.91 | 0.1016 | |
| 57509 | | MTUS1 | 0.72 | | | 0.0000 | | **0.62** | | **0.0000** | 0.87 | 0.0388 | |
| 57530 | | CGN | 1.17 | | | 0.1568 | | **1.44** | | **0.0004** | 1.16 | 0.0005 | |
| 57546 | | PDP2 | 0.82 | | | 0.0057 | | **0.68** | | **0.0004** | 0.98 | 0.9999 | |
| 57678 | | GPAM | 0.75 | | | 0.0118 | | **0.60** | | **0.0005** | 0.72 | 0.0041 | |
| 58510 | | PRODH2 | 0.72 | | | 0.0004 | | **0.60** | | **0.0000** | 0.94 | 0.1402 | |
| 58528 | | RRAGD | 1.03 | | | 0.4416 | | **1.63** | | **0.0000** | 1.08 | 0.1545 | |
| 58986 | | TMEM8A | 0.78 | | | 0.0068 | | **0.64** | | **0.0006** | 0.97 | 0.2771 | |
| 59277 | | NTN4 | 1.14 | | | 0.4354 | | **1.70** | | **0.0008** | 1.39 | 0.0011 | |
| 60386 | | SLC25A19 | 0.76 | | | 0.0004 | | **0.70** | | **0.0007** | 0.88 | 0.1381 | |
| 60676 | | PAPPA2 | 1.25 | | | 0.0290 | | **2.46** | | **0.0000** | 1.46 | 0.0029 | |
| 63826 | | SRR | 0.74 | | | 0.0000 | | **0.65** | | **0.0008** | 0.77 | 0.0003 | |
| 63923 | | TNN | 0.92 | | | 0.1342 | | **0.56** | | **0.0000** | 0.89 | 0.0502 | |
| 64122 | | FN3K | 0.73 | | | 0.0000 | | **0.53** | | **0.0000** | 0.82 | 0.0011 | |
| 64207 | | IRF2BPL | 1.16 | | | 0.2051 | | **1.47** | | **0.0006** | 1.12 | 0.1837 | |
| 64420 | | SUSD1 | 1.19 | | | 0.0593 | | **1.42** | | **0.0007** | 1.20 | 0.0681 | |
| 64919 | | BCL11B | 0.95 | | | 0.2014 | | **0.70** | | **0.0004** | 0.94 | 0.1110 | |
| 64981 | | MRPL34 | 0.79 | | | 0.0007 | | **0.68** | | **0.0007** | 0.92 | 0.1292 | |
| 65009 | | NDRG4 | 0.74 | | | 0.0000 | | **0.47** | | **0.0000** | 0.74 | 0.0007 | |
| 65985 | | AACS | 0.75 | | | 0.0068 | | **0.42** | | **0.0000** | 0.70 | 0.0079 | |
| 79038 | | ZFYVE21 | 0.77 | | | 0.0106 | | **0.70** | | **0.0004** | 0.96 | 0.2561 | |
| 79056 | | PRRG4 | 1.22 | | | 0.0593 | | **1.85** | | **0.0000** | 1.13 | 0.0219 | |
| 79065 | | ATG9A | 0.90 | | | 0.0880 | | **0.69** | | **0.0000** | 0.90 | 0.0281 | |
| 79085 | | SLC25A23 | 0.87 | | | 0.0976 | | **0.65** | | **0.0004** | 0.83 | 0.0013 | |
| 79152 | | FA2H | 0.88 | | | 0.0750 | | **0.67** | | **0.0004** | 0.79 | 0.0000 | |
| 79155 | | TNIP2 | 1.24 | | | 0.0024 | | **1.42** | | **0.0000** | 1.09 | 0.0281 | |
| 79570 | | NKAIN1 | 0.90 | | | 0.0207 | | **1.66** | | **0.0009** | 0.92 | 0.1203 | |
| 79605 | | PGBD5 | 1.11 | | | 0.1531 | | **1.58** | | **0.0007** | 1.26 | 0.0003 | |
| 79623 | | GALNT14 | 0.78 | | | 0.0000 | | **0.61** | | **0.0004** | 0.84 | 0.0213 | |
| 79658 | | ARHGAP10 | 0.88 | | | 0.0692 | | **0.59** | | **0.0000** | 0.91 | 0.1184 | |
| 79668 | | PARP8 | 1.29 | | | 0.0006 | | **1.90** | | **0.0005** | 1.18 | 0.0073 | |
| 79736 | | TEFM | 0.72 | | | 0.0004 | | **0.69** | | **0.0009** | 0.88 | 0.1000 | |
| 79746 | | ECHDC3 | 0.75 | | | 0.0006 | | **0.64** | | **0.0000** | 0.78 | 0.0000 | |
| 79774 | | GRTP1 | 0.73 | | | 0.0000 | | **0.71** | | **0.0000** | 0.85 | 0.0681 | |
| 79792 | | GSDMD | 1.32 | | | 0.0000 | | **1.58** | | **0.0009** | 1.22 | 0.0000 | |
| 79815 | | NIPAL2 | 1.31 | | | 0.0065 | | **1.95** | | **0.0000** | 1.32 | 0.0000 | |
| 79828 | | METTL8 | 0.75 | | | 0.0014 | | **0.69** | | **0.0005** | 0.72 | 0.0000 | |
| 79882 | | ZC3H14 | 0.86 | | | 0.0000 | | **0.69** | | **0.0000** | 0.87 | 0.0046 | |
| 79917 | | MAGIX | 0.85 | | | 0.0131 | | **0.60** | | **0.0000** | 0.78 | 0.0024 | |
| 79919 | | C2orf54 | 0.76 | | | 0.0011 | | **0.55** | | **0.0000** | 1.17 | 0.0198 | |
| 80017 | | C14orf159 | 0.76 | | | 0.0018 | | **0.61** | | **0.0000** | 0.84 | 0.0019 | |
| 80167 | | C4orf29 | 0.79 | | | 0.0000 | | **0.58** | | **0.0000** | 0.81 | 0.0019 | |
| 80237 | | ELL3 | 0.69 | | | 0.0013 | | **0.49** | | **0.0000** | 0.80 | 0.0066 | |
| 80298 | | MTERFD3 | 0.79 | | | 0.0258 | | **0.66** | | **0.0009** | 0.91 | 0.1381 | |
| 80347 | | COASY | 0.82 | | | 0.0472 | | **0.69** | | **0.0000** | 0.77 | 0.0000 | |
| 80790 | | CMIP | 0.77 | | | 0.0017 | | **0.64** | | **0.0004** | 0.88 | 0.0143 | |
| 81029 | | WNT5B | 0.88 | | | 0.0240 | | **0.67** | | **0.0005** | 0.89 | 0.0241 | |
| 81537 | | SGPP1 | 0.76 | | | 0.0015 | | **0.71** | | **0.0000** | 0.74 | 0.0000 | |
| 81579 | | PLA2G12A | 0.78 | | | 0.0004 | | **0.69** | | **0.0004** | 0.93 | 0.0443 | |
| 81671 | | VMP1 | 1.06 | | | 0.4309 | | **1.47** | | **0.0000** | 1.16 | 0.0023 | |
| 81932 | | HDHD3 | 0.72 | | | 0.0000 | | **0.65** | | **0.0004** | 0.95 | 0.9999 | |
| 83715 | | ESPN | 0.86 | | | 0.0492 | | **0.63** | | **0.0004** | 0.95 | 0.2291 | |
| 83877 | | TM2D2 | 0.77 | | | 0.0053 | | **0.60** | | **0.0000** | 0.82 | 0.0019 | |
| 83878 | | USHBP1 | 0.91 | | | 0.0880 | | **0.70** | | **0.0009** | 0.84 | 0.0143 | |
| 83892 | | KCTD10 | 1.37 | | | 0.0000 | | **1.43** | | **0.0008** | 1.15 | 0.0070 | |
| 84140 | | FAM161A | 0.94 | | | 0.1265 | | **0.69** | | **0.0000** | 0.85 | 0.0032 | |
| 84266 | | ALKBH7 | 0.76 | | | 0.0000 | | **0.65** | | **0.0000** | 0.91 | 0.0120 | |
| 84275 | | SLC25A33 | 0.69 | | | 0.0031 | | **0.61** | | **0.0000** | 0.74 | 0.0000 | |
| 84281 | | C2orf88 | np | | | | | **0.60** | | **0.0005** | np | | |
| 84461 | | NEURL4 | 0.83 | | | 0.0113 | | **0.68** | | **0.0005** | 0.91 | 0.1657 | |
| 84525 | | HOPX | 0.93 | | | 0.2051 | | **0.70** | | **0.0006** | 0.86 | 0.0055 | |
| 84812 | | PLCD4 | 0.97 | | | 0.3136 | | **0.69** | | **0.0006** | 0.85 | 0.0302 | |
| 84816 | | RTN4IP1 | 0.73 | | | 0.0000 | | **0.64** | | **0.0006** | 0.72 | 0.0007 | |
| 84818 | | IL17RC | 1.08 | | | 0.3500 | | **1.50** | | **0.0006** | 1.12 | 0.0053 | |
| 84836 | | ABHD14B | 0.84 | | | 0.0164 | | **0.68** | | **0.0005** | 0.87 | 0.0230 | |
| 84879 | | MFSD2A | 0.61 | | | 0.0023 | | **0.29** | | **0.0000** | 0.36 | 0.0095 | |
| 84895 | | FAM73B | 0.81 | | | 0.0007 | | **0.58** | | **0.0000** | 0.76 | 0.0003 | |
| 84904 | | C9orf100 | 0.84 | | | 0.0038 | | **0.61** | | **0.0000** | 1.08 | 0.9999 | |
| 84918 | | LRP11 | 0.93 | | | 0.2127 | | **1.46** | | **0.0000** | 0.99 | 0.9999 | |
| 85458 | | DIXDC1 | 0.86 | | | 0.0068 | | **0.67** | | **0.0000** | 0.83 | 0.0104 | |
| 85477 | | SCIN | 1.29 | | | 0.0258 | | **2.52** | | **0.0005** | 1.30 | 0.0005 | |
| 85865 | | GTPBP10 | 0.87 | | | 0.0306 | | **0.65** | | **0.0000** | 0.84 | 0.0039 | |
| 90161 | | HS6ST2 | 0.90 | | | 0.0880 | | **0.70** | | **0.0008** | 0.89 | 0.1402 | |
| 90288 | | C3orf25 | np | | | | | **0.70** | | **0.0009** | np | | |
| 90637 | | ZFAND2A | 1.20 | | | 0.0021 | | **1.51** | | **0.0000** | 0.95 | 0.9999 | |
| 90861 | | HN1L | 1.24 | | | 0.2460 | | **1.80** | | **0.0000** | 1.32 | 0.0057 | |
| 91107 | | TRIM47 | 1.33 | | | 0.0000 | | **2.05** | | **0.0005** | 1.27 | 0.0456 | |
| 91419 | | XRCC6BP1 | 0.72 | | | 0.0004 | | **0.67** | | **0.0000** | 0.86 | 0.0273 | |
| 94032 | | CAMK2N2 | 1.19 | | | 0.0297 | | **2.10** | | **0.0000** | 1.23 | 0.0298 | |
| 112483 | | SAT2 | 0.83 | | | 0.0186 | | **0.65** | | **0.0004** | 0.78 | 0.0060 | |
| 112817 | | HOGA1 | 0.78 | | | 0.0000 | | **0.59** | | **0.0000** | 0.81 | 0.0005 | |
| 113235 | | SLC46A1 | 0.76 | | | 0.0027 | | **0.55** | | **0.0000** | 0.81 | 0.0005 | |
| 114569 | | MAL2 | 1.28 | | | 0.2163 | | **1.97** | | **0.0006** | 1.48 | 0.0024 | |
| 114876 | | OSBPL1A | 0.75 | | | 0.0006 | | **0.64** | | **0.0000** | 0.73 | 0.0000 | |
| 114879 | | OSBPL5 | 0.77 | | | 0.0000 | | **0.70** | | **0.0000** | 1.01 | 0.3749 | |
| 115795 | | C3orf24 | 0.77 | | | 0.0000 | | **0.62** | | **0.0000** | 0.77 | 0.0112 | |
| 116039 | | OSR2 | 0.71 | | | 0.0017 | | **0.44** | | **0.0000** | 0.98 | 0.9999 | |
| 116085 | | SLC22A12 | 0.72 | | | 0.0065 | | **0.53** | | **0.0004** | 0.81 | 0.0000 | |
| 118672 | | PSTK | 0.80 | | | 0.0011 | | **0.71** | | **0.0005** | 0.95 | 0.2041 | |
| 118980 | | SFXN2 | 0.83 | | | 0.0077 | | **0.69** | | **0.0005** | 0.90 | 0.0905 | |
| 119391 | | GSTO2 | 0.82 | | | 0.0000 | | **0.58** | | **0.0000** | 0.77 | 0.0000 | |
| 120224 | | TMEM45B | 0.88 | | | 0.0660 | | **1.63** | | **0.0009** | 0.92 | 0.1657 | |
| 122481 | | AK7 | 1.26 | | | 0.0240 | | **1.45** | | **0.0000** | 1.16 | 0.0173 | |
| 122616 | | C14orf79 | 0.72 | | | 0.0004 | | **0.69** | | **0.0000** | 1.00 | 0.9999 | |
| 123264 | | OSTBETA | 0.82 | | | 0.0133 | | **0.64** | | **0.0007** | 0.93 | 0.0399 | |
| 123283 | | TARSL2 | 0.81 | | | 0.0003 | | **0.71** | | **0.0008** | 0.84 | 0.0007 | |
| 127253 | | TYW3 | 0.64 | | | 0.0013 | | **0.45** | | **0.0000** | 0.81 | 0.1460 | |
| 128272 | | ARHGEF19 | 0.89 | | | 0.0199 | | **0.70** | | **0.0009** | 0.93 | 0.1110 | |
| 131669 | | UROC1 | 1.13 | | | 0.0692 | | **0.67** | | **0.0006** | 0.87 | 0.0959 | |
| 132160 | | PPM1M | 1.21 | | | 0.0019 | | **1.57** | | **0.0006** | 1.03 | 0.3620 | |
| 133418 | | EMB | 1.15 | | | 0.2460 | | **1.67** | | **0.0006** | 1.23 | 0.0009 | |
| 134285 | | TMEM171 | 1.22 | | | 0.1132 | | **1.64** | | **0.0000** | 1.05 | 0.1770 | |
| 136288 | | C7orf57 | 0.69 | | | 0.0129 | | **0.54** | | **0.0000** | 0.67 | 0.0011 | |
| 137682 | | C8orf38 | 0.73 | | | 0.0044 | | **0.70** | | **0.0005** | 0.83 | 0.0003 | |
| 137886 | | UBXN2B | 0.62 | | | 0.0039 | | **0.66** | | **0.0009** | 0.90 | 0.1626 | |
| 138639 | | PTPDC1 | 0.83 | | | 0.0075 | | **0.71** | | **0.0005** | 0.81 | 0.0005 | |
| 140462 | | ASB9 | 0.80 | | | 0.0031 | | **0.59** | | **0.0004** | 0.83 | 0.0044 | |
| 140809 | | SRXN1 | 1.14 | | | 0.3856 | | **1.67** | | **0.0000** | 1.34 | 0.0262 | |
| 143458 | | LDLRAD3 | 0.79 | | | 0.0015 | | **0.67** | | **0.0000** | 0.84 | 0.0000 | |
| 144363 | | LYRM5 | 0.74 | | | 0.0023 | | **0.66** | | **0.0007** | 0.72 | 0.0000 | |
| 146894 | | CD300LG | 1.08 | | | 0.4000 | | **0.55** | | **0.0000** | 0.79 | 0.0005 | |
| 147700 | | KLC3 | 1.30 | | | 0.0015 | | **1.68** | | **0.0000** | 1.24 | 0.0000 | |
| 147798 | | TMC4 | 1.06 | | | 0.4089 | | **1.47** | | **0.0005** | 1.05 | 0.2382 | |
| 148753 | | FAM163A | 0.75 | | | 0.0000 | | **0.58** | | **0.0004** | 1.06 | 0.2832 | |
| 148867 | | SLC30A7 | 1.21 | | | 0.0207 | | **1.41** | | **0.0007** | 1.14 | 0.0114 | |
| 150084 | | IGSF5 | 1.08 | | | 0.4267 | | **1.94** | | **0.0000** | 1.34 | 0.0017 | |
| 150209 | | AIFM3 | 0.83 | | | 0.0041 | | **0.63** | | **0.0005** | 0.77 | 0.0015 | |
| 153768 | | PRELID2 | 1.21 | | | 0.0918 | | **1.77** | | **0.0004** | 1.27 | 0.0031 | |
| 154141 | | MBOAT1 | 1.37 | | | 0.0199 | | **2.11** | | **0.0006** | 1.36 | 0.0000 | |
| 154791 | | C7orf55 | 0.78 | | | 0.0029 | | **0.59** | | **0.0000** | 0.88 | 0.0009 | |
| 157506 | | RDH10 | 1.10 | | | 0.4050 | | **1.90** | | **0.0000** | 1.14 | 0.1311 | |
| 157724 | | SLC7A13 | 0.88 | | | 0.0807 | | **0.37** | | **0.0000** | 0.59 | 0.0038 | |
| 160140 | | C11orf65 | np | | | | | **1.53** | | **0.0007** | 1.15 | 0.0057 | |
| 161247 | | FITM1 | 0.75 | | | 0.0053 | | **0.56** | | **0.0004** | 0.63 | 0.0032 | |
| 166785 | | MMAA | 0.78 | | | 0.0008 | | **0.66** | | **0.0004** | 0.75 | 0.0029 | |
| 169693 | | C9orf71 | 1.35 | | | 0.1531 | | **3.07** | | **0.0000** | 1.20 | 0.3268 | |
| 170690 | | ADAMTS16 | 1.35 | | | 0.0352 | | **2.01** | | **0.0000** | 1.36 | 0.0000 | |
| 197322 | | ACSF3 | 0.90 | | | 0.0660 | | **0.71** | | **0.0007** | 0.75 | 0.0000 | |
| 201191 | | SAMD14 | 1.22 | | | 0.0013 | | **1.79** | | **0.0009** | 1.24 | 0.0004 | |
| 202151 | | RANBP3L | 0.75 | | | 0.0164 | | **0.49** | | **0.0000** | 0.75 | 0.0055 | |
| 203260 | | CCDC107 | 0.75 | | | 0.0041 | | **0.62** | | **0.0000** | 0.81 | 0.0003 | |
| 245930 | | DEFB116 | 0.76 | | | 0.0258 | | **0.39** | | **0.0000** | 0.90 | 0.2618 | |
| 253512 | | SLC25A30 | 1.39 | | | 0.3382 | | **2.34** | | **0.0000** | 1.09 | 0.3474 | |
| 254013 | | METTL20 | 0.87 | | | 0.1110 | | **0.69** | | **0.0004** | 0.89 | 0.1230 | |
| 256126 | | SYCE2 | 1.24 | | | 0.0179 | | **1.78** | | **0.0000** | 1.39 | 0.0003 | |
| 259217 | | HSPA12A | 0.87 | | | 0.0106 | | **0.65** | | **0.0005** | 0.92 | 0.0905 | |
| 283537 | | SLC46A3 | 0.99 | | | 0.3713 | | **0.59** | | **0.0006** | 0.81 | 0.0751 | |
| 284106 | | CISD3 | 0.79 | | | 0.0031 | | **0.65** | | **0.0005** | 0.83 | 0.0005 | |
| 284207 | | METRNL | 0.82 | | | 0.0080 | | **0.66** | | **0.0005** | 0.82 | 0.0173 | |
| 284611 | | FAM102B | 0.94 | | | 0.1715 | | **1.46** | | **0.0009** | 1.14 | 0.0112 | |
| 285343 | | C3orf23 | 0.72 | | | 0.0013 | | **0.69** | | **0.0004** | 0.77 | 0.0062 | |
| 326625 | | MMAB | 0.82 | | | 0.0000 | | **0.63** | | **0.0000** | 0.85 | 0.0005 | |
| 333926 | | PPM1J | 1.12 | | | 0.1083 | | **1.74** | | **0.0005** | 1.21 | 0.0039 | |
| 339229 | | C17orf90 | 0.85 | | | 0.0258 | | **0.62** | | **0.0000** | 0.74 | 0.0000 | |
| 341640 | | FREM2 | 0.91 | | | 0.1064 | | **0.68** | | **0.0000** | 0.96 | 0.1956 | |
| 348093 | | RBPMS2 | 0.84 | | | 0.0149 | | **0.68** | | **0.0006** | 0.86 | 0.0019 | |
| 374291 | | NDUFS7 | 0.74 | | | 0.0007 | | **0.66** | | **0.0000** | 0.86 | 0.0003 | |
| 375775 | | PNPLA7 | 0.89 | | | 0.0781 | | **0.66** | | **0.0007** | 0.73 | 0.0007 | |
| 377841 | | ENTPD8 | 0.79 | | | 0.0016 | | **0.61** | | **0.0006** | 0.92 | 0.0784 | |
| 387521 | | TMEM189 | 1.04 | | | 0.4330 | | **0.62** | | **0.0000** | 0.99 | 0.9999 | |
| 387893 | | SETD8 | 0.92 | | | 0.1843 | | **0.69** | | **0.0007** | 0.99 | 0.9999 | |
| 388228 | | SBK1 | 0.75 | | | 0.0004 | | **0.71** | | **0.0004** | 0.88 | 0.0502 | |
| 388753 | | C1orf31 | 0.66 | | | 0.0019 | | **0.57** | | **0.0000** | 0.85 | 0.0273 | |
| 392617 | | ELFN1 | 0.77 | | | 0.0006 | | **0.67** | | **0.0007** | 0.88 | 0.0189 | |
| 399512 | | SLC25A35 | 0.74 | | | 0.0017 | | **0.61** | | **0.0000** | 0.76 | 0.0000 | |
| 401097 | | C3orf80 | 0.72 | | | 0.0068 | | **0.46** | | **0.0000** | 1.12 | 0.3655 | |
| 414919 | | C8orf82 | 0.77 | | | 0.0000 | | **0.62** | | **0.0000** | 0.73 | 0.0000 | |
| 1E+08 | | LOC100129480 | 1.06 | | | 0.4454 | | **1.96** | | **0.0005** | 1.11 | 0.0289 | |
| 1E+08 | | TSTD1 | 0.86 | | | 0.0900 | | **1.85** | | **0.0000** | 1.09 | 0.2798 | |
| 23779 | | ARHGAP8 | 1.27 | | | 0.0029 | | **1.57** | | **0.0000** | 1.12 | 0.0044 | |
| **C. 262 genes regulated only in NZW/BXSB 18-21 weeks old vs. 8+17 wks old.** | | | | | | | | | | | | | |
| Human Entrez Gene ID | | Human Gene symbol | **NZB/W** | | | | | **NZM2410** | | | **NZW/BXSB** | | |
|  |  |  | Fold-change | | | q-value | | Fold-change | | q-value | Fold-change | q-value | |
| 59 | | ACTA2 | 1.24 | | | 0.2501 | | 2.31 | | 0.0032 | **2.23** | **0.0000** | |
| 101 | | ADAM8 | 1.34 | | | 0.0207 | | 3.48 | | 0.0024 | **1.68** | **0.0000** | |
| 309 | | ANXA6 | 1.27 | | | 0.0060 | | 1.25 | | 0.0139 | **1.40** | **0.0000** | |
| 350 | | APOH | 1.29 | | | 0.0186 | | 1.35 | | 0.1590 | **0.52** | **0.0003** | |
| 627 | | BDNF | 0.84 | | | 0.0035 | | 0.69 | | 0.0012 | **0.59** | **0.0000** | |
| 672 | | BRCA1 | np | | | | | np | | | **1.51** | **0.0005** | |
| 701 | | BUB1B | 1.10 | | | 0.2422 | | 1.24 | | 0.0101 | **1.52** | **0.0004** | |
| 717 | | C2 | 1.17 | | | 0.1531 | | 3.05 | | 0.0019 | **1.59** | **0.0000** | |
| 728 | | C5AR1 | 1.31 | | | 0.0004 | | 1.14 | | 0.1394 | **1.44** | **0.0000** | |
| 731 | | C8A | 0.64 | | | 0.0044 | | 0.74 | | 0.0474 | **0.46** | **0.0000** | |
| 733 | | C8G | 0.83 | | | 0.0250 | | 0.68 | | 0.0028 | **0.58** | **0.0000** | |
| 768 | | CA9 | 1.00 | | | 0.9999 | | 0.90 | | 0.1440 | **0.46** | **0.0000** | |
| 948 | | CD36 | 1.06 | | | 0.4309 | | 0.88 | | 0.2100 | **0.62** | **0.0000** | |
| 1009 | | CDH11 | 1.85 | | | 0.0122 | | 1.20 | | 0.3022 | **1.96** | **0.0003** | |
| 1033 | | CDKN3 | 1.29 | | | 0.0055 | | 1.33 | | 0.0104 | **1.84** | **0.0005** | |
| 1036 | | CDO1 | 1.24 | | | 0.2665 | | 1.16 | | 0.1851 | **0.70** | **0.0003** | |
| 1058 | | CENPA | 1.49 | | | 0.0014 | | 1.32 | | 0.0349 | **2.05** | **0.0000** | |
| 1111 | | CHEK1 | np | | | | | np | | | **1.49** | **0.0004** | |
| 1277 | | COL1A1 | 2.68 | | | 0.0015 | | 1.41 | | 0.2517 | **3.62** | **0.0000** | |
| 1281 | | COL3A1 | 2.68 | | | 0.0013 | | 1.17 | | 0.4415 | **3.36** | **0.0000** | |
| 1289 | | COL5A1 | 1.43 | | | 0.0250 | | 1.03 | | 0.4772 | **2.04** | **0.0000** | |
| 1290 | | COL5A2 | 1.02 | | | 0.9999 | | 0.83 | | 0.0397 | **1.68** | **0.0000** | |
| 1292 | | COL6A2 | 1.41 | | | 0.0097 | | 1.12 | | 0.4009 | **1.85** | **0.0000** | |
| 1293 | | COL6A3 | 1.15 | | | 0.4089 | | 1.05 | | 0.9999 | **2.03** | **0.0003** | |
| 1295 | | COL8A1 | 1.45 | | | 0.0164 | | 1.03 | | 0.9999 | **1.79** | **0.0000** | |
| 1303 | | COL12A1 | 1.38 | | | 0.0068 | | 1.11 | | 0.3826 | **1.60** | **0.0000** | |
| 1306 | | COL15A1 | 1.48 | | | 0.0472 | | 1.07 | | 0.4703 | **2.30** | **0.0005** | |
| 1369 | | CPN1 | 0.84 | | | 0.0240 | | 0.97 | | 0.3296 | **1.88** | **0.0000** | |
| 1376 | | CPT2 | 0.91 | | | 0.1641 | | 0.83 | | 0.0133 | **0.69** | **0.0009** | |
| 1468 | | SLC25A10 | 0.79 | | | 0.0100 | | 0.79 | | 0.0254 | **0.69** | **0.0000** | |
| 1565 | | CYP2D6 | 0.78 | | | 0.0505 | | 0.87 | | 0.1491 | **0.22** | **0.0009** | |
| 1572 | | CYP2F1 | 0.76 | | | 0.0138 | | 0.24 | | 0.3166 | **0.51** | **0.0000** | |
| 1666 | | DECR1 | 0.86 | | | 0.0707 | | 0.97 | | 0.3826 | **0.65** | **0.0000** | |
| 1763 | | DNA2 | 1.01 | | | 0.9999 | | 0.85 | | 0.0166 | **1.47** | **0.0000** | |
| 1786 | | DNMT1 | 1.28 | | | 0.0004 | | 1.25 | | 0.0017 | **1.43** | **0.0000** | |
| 1978 | | EIF4EBP1 | 1.34 | | | 0.0021 | | 1.58 | | 0.0051 | **1.44** | **0.0003** | |
| 1992 | | SERPINB1 | 1.24 | | | 0.1437 | | 1.47 | | 0.0203 | **1.45** | **0.0000** | |
| 2012 | | EMP1 | 1.25 | | | 0.0173 | | 1.34 | | 0.0013 | **1.60** | **0.0000** | |
| 2053 | | EPHX2 | 0.75 | | | 0.0024 | | 0.79 | | 0.0112 | **0.63** | **0.0000** | |
| 2146 | | EZH2 | 1.30 | | | 0.0593 | | 1.04 | | 0.4334 | **1.55** | **0.0007** | |
| 2162 | | F13A1 | 1.82 | | | 0.0429 | | 1.95 | | 0.1097 | **1.98** | **0.0005** | |
| 2173 | | FABP7 | 0.78 | | | 0.0781 | | 1.17 | | 0.3296 | **1.51** | **0.0008** | |
| 2202 | | EFEMP1 | 1.26 | | | 0.1150 | | 2.06 | | 0.0015 | **1.62** | **0.0002** | |
| 2254 | | FGF9 | 0.75 | | | 0.0013 | | 0.79 | | 0.0407 | **0.65** | **0.0000** | |
| 2491 | | CENPI | np | | | | | np | | | **1.91** | **0.0000** | |
| 2634 | | GBP2 | 1.15 | | | 0.1132 | | np | | | **2.30** | **0.0000** | |
| 2638 | | GC | 0.99 | | | 0.2903 | | 1.57 | | 0.0082 | **3.03** | **0.0000** | |
| 2642 | | GCGR | 0.82 | | | 0.0048 | | 0.86 | | 0.0654 | **0.71** | **0.0000** | |
| 2820 | | GPD2 | 0.95 | | | 0.2579 | | 1.06 | | 0.3922 | **0.65** | **0.0000** | |
| 2882 | | GPX7 | 1.22 | | | 0.0264 | | 1.04 | | 0.3091 | **1.51** | **0.0000** | |
| 2982 | | GUCY1A3 | 1.31 | | | 0.0297 | | 0.96 | | 0.4009 | **1.50** | **0.0000** | |
| 3014 | | H2AFX | 1.34 | | | 0.0029 | | 1.17 | | 0.0490 | **1.46** | **0.0003** | |
| 3070 | | HELLS | 1.67 | | | 0.0011 | | 1.80 | | 0.0016 | **3.38** | **0.0000** | |
| 3161 | | HMMR | 1.17 | | | 0.0282 | | 1.06 | | 0.3754 | **1.57** | **0.0003** | |
| 3294 | | HSD17B2 | 0.84 | | | 0.0368 | | 0.94 | | 0.3616 | **0.57** | **0.0000** | |
| 3301 | | DNAJA1 | 0.79 | | | 0.0091 | | 0.78 | | 0.0161 | **0.67** | **0.0006** | |
| 3486 | | IGFBP3 | 0.89 | | | 0.1377 | | 0.39 | | 0.0017 | **0.43** | **0.0000** | |
| 3569 | | IL6 | np | | | | | np | | | **1.99** | **0.0003** | |
| 3619 | | INCENP | 1.15 | | | 0.0122 | | 1.31 | | 0.0005 | **1.52** | **0.0000** | |
| 3655 | | ITGA6 | 1.14 | | | 0.3083 | | 1.30 | | 0.0583 | **1.54** | **0.0000** | |
| 3772 | | KCNJ15 | 0.72 | | | 0.0021 | | 0.74 | | 0.0221 | **0.71** | **0.0000** | |
| 3832 | | KIF11 | 1.11 | | | 0.2293 | | np | | | **1.41** | **0.0009** | |
| 3835 | | KIF22 | 1.29 | | | 0.0149 | | 1.30 | | 0.0016 | **1.50** | **0.0003** | |
| 3903 | | LAIR1 | 1.48 | | | 0.0015 | | 1.64 | | 0.0028 | **1.43** | **0.0000** | |
| 3975 | | LHX1 | 0.80 | | | 0.0091 | | 0.76 | | 0.0027 | **0.66** | **0.0000** | |
| 3978 | | LIG1 | 1.32 | | | 0.0014 | | 1.44 | | 0.0014 | **1.63** | **0.0000** | |
| 3991 | | LIPE | 0.86 | | | 0.1083 | | 0.71 | | 0.0397 | **0.66** | **0.0004** | |
| 4000 | | LMNA | 1.39 | | | 0.0000 | | 1.58 | | 0.0032 | **1.43** | **0.0000** | |
| 4017 | | LOXL2 | 1.20 | | | 0.0440 | | 1.58 | | 0.0049 | **1.66** | **0.0000** | |
| 4056 | | LTC4S | 1.27 | | | 0.0149 | | 1.66 | | 0.0013 | **1.45** | **0.0000** | |
| 4129 | | MAOB | 0.79 | | | 0.0041 | | 0.69 | | 0.0161 | **0.70** | **0.0000** | |
| 4162 | | MCAM | 1.29 | | | 0.0131 | | 1.40 | | 0.0139 | **1.62** | **0.0000** | |
| 4171 | | MCM2 | 1.33 | | | 0.0024 | | 1.24 | | 0.0212 | **1.67** | **0.0002** | |
| 4172 | | MCM3 | 1.39 | | | 0.0004 | | 1.22 | | 0.0315 | **1.78** | **0.0000** | |
| 4175 | | MCM6 | 1.30 | | | 0.1408 | | 1.39 | | 0.0084 | **2.01** | **0.0000** | |
| 4176 | | MCM7 | 1.33 | | | 0.0000 | | 1.37 | | 0.0000 | **1.47** | **0.0003** | |
| 4192 | | MDK | 1.01 | | | 0.3334 | | 0.79 | | 0.0851 | **1.95** | **0.0000** | |
| 4478 | | MSN | 1.45 | | | 0.0023 | | 1.53 | | 0.0012 | **1.70** | **0.0000** | |
| 4481 | | MSR1 | 1.37 | | | 0.0023 | | 1.37 | | 0.0004 | **1.46** | **0.0000** | |
| 4542 | | MYO1F | 1.42 | | | 0.0018 | | np | | | **1.44** | **0.0003** | |
| 4582 | | MUC1 | 1.22 | | | 0.0173 | | 1.22 | | 0.1394 | **1.44** | **0.0000** | |
| 4854 | | NOTCH3 | 1.19 | | | 0.0297 | | 1.11 | | 0.2267 | **1.41** | **0.0000** | |
| 4939 | | OAS2 | 1.36 | | | 0.0019 | | 1.58 | | 0.0012 | **1.40** | **0.0005** | |
| 5105 | | PCK1 | 0.60 | | | 0.0017 | | 0.61 | | 0.0184 | **0.41** | **0.0000** | |
| 5118 | | PCOLCE | 1.07 | | | 0.3176 | | 1.21 | | 0.0290 | **1.46** | **0.0000** | |
| 5155 | | PDGFB | 1.30 | | | 0.0623 | | 1.35 | | 0.0028 | **1.53** | **0.0003** | |
| 5159 | | PDGFRB | 1.42 | | | 0.0018 | | 1.14 | | 0.3166 | **1.49** | **0.0000** | |
| 5214 | | PFKP | 1.13 | | | 0.2945 | | 1.38 | | 0.0000 | **1.55** | **0.0000** | |
| 5216 | | PFN1 | 1.37 | | | 0.0640 | | 1.53 | | 0.0203 | **1.40** | **0.0000** | |
| 5257 | | PHKB | 0.68 | | | 0.0034 | | 0.62 | | 0.0017 | **0.63** | **0.0009** | |
| 5265 | | SERPINA1 | 1.33 | | | 0.2986 | | 1.04 | | 0.4703 | **0.40** | **0.0003** | |
| 5322 | | PLA2G5 | 0.75 | | | 0.0004 | | 0.82 | | 0.0162 | **1.43** | **0.0000** | |
| 5359 | | PLSCR1 | 1.13 | | | 0.2797 | | 1.93 | | 0.0012 | **1.49** | **0.0000** | |
| 5422 | | POLA1 | 1.30 | | | 0.0106 | | 1.41 | | 0.0017 | **1.84** | **0.0000** | |
| 5426 | | POLE | 1.07 | | | 0.3136 | | 1.22 | | 0.0156 | **1.73** | **0.0000** | |
| 5557 | | PRIM1 | 1.04 | | | 0.4354 | | 1.07 | | 0.1638 | **1.44** | **0.0002** | |
| 5641 | | LGMN | 1.02 | | | 0.9999 | | 1.21 | | 0.0556 | **1.47** | **0.0000** | |
| 5649 | | RELN | 0.94 | | | 0.2260 | | 0.75 | | 0.0066 | **0.67** | **0.0006** | |
| 5909 | | RAP1GAP | 0.87 | | | 0.0660 | | 0.79 | | 0.0012 | **0.70** | **0.0000** | |
| 5933 | | RBL1 | 1.11 | | | 0.3806 | | 1.22 | | 0.0242 | **1.62** | **0.0000** | |
| 5947 | | RBP1 | 1.26 | | | 0.0660 | | 0.89 | | 0.2327 | **1.58** | **0.0000** | |
| 5983 | | RFC3 | 1.12 | | | 0.1740 | | 1.25 | | 0.0087 | **1.42** | **0.0000** | |
| 5984 | | RFC4 | 1.07 | | | 0.4498 | | 1.06 | | 0.3754 | **1.42** | **0.0000** | |
| 6123 | | RPL3L | np | | | | | np | | | **0.67** | **0.0000** | |
| 6240 | | RRM1 | 1.04 | | | 0.4035 | | 1.01 | | 0.9999 | **1.48** | **0.0000** | |
| 6521 | | SLC4A1 | 0.73 | | | 0.0100 | | 0.66 | | 0.0012 | **0.57** | **0.0000** | |
| 6790 | | AURKA | 1.12 | | | 0.1976 | | 1.27 | | 0.0084 | **1.93** | **0.0005** | |
| 7041 | | TGFB1I1 | 1.29 | | | 0.0314 | | 1.47 | | 0.0016 | **1.51** | **0.0000** | |
| 7096 | | TLR1 | 1.48 | | | 0.0068 | | np | | | **1.52** | **0.0000** | |
| 7162 | | TPBG | np | | | | | np | | | **1.44** | **0.0000** | |
| 7169 | | TPM2 | 1.14 | | | 0.1496 | | 1.57 | | 0.0337 | **1.51** | **0.0000** | |
| 7272 | | TTK | 1.25 | | | 0.0306 | | 1.07 | | 0.3540 | **1.46** | **0.0008** | |
| 7350 | | UCP1 | 1.30 | | | 0.3713 | | 0.30 | | 0.0684 | **0.36** | **0.0005** | |
| 7791 | | ZYX | 1.33 | | | 0.0000 | | 1.40 | | 0.0129 | **1.45** | **0.0000** | |
| 8038 | | ADAM12 | 1.15 | | | 0.3044 | | 1.08 | | 0.4009 | **1.58** | **0.0000** | |
| 8208 | | CHAF1B | 1.15 | | | 0.0264 | | 1.29 | | 0.0040 | **1.46** | **0.0000** | |
| 8291 | | DYSF | 1.31 | | | 0.0006 | | 1.35 | | 0.0028 | **1.49** | **0.0000** | |
| 8564 | | KMO | 0.71 | | | 0.0068 | | 0.75 | | 0.0397 | **0.65** | **0.0000** | |
| 8840 | | WISP1 | 1.09 | | | 0.3550 | | 1.02 | | 0.9999 | **1.64** | **0.0007** | |
| 9134 | | CCNE2 | np | | | | | np | | | **1.54** | **0.0003** | |
| 9212 | | AURKB | np | | | | | np | | | **1.81** | **0.0000** | |
| 9244 | | CRLF1 | 1.26 | | | 0.0440 | | 1.46 | | 0.1534 | **1.85** | **0.0000** | |
| 9319 | | TRIP13 | np | | | | | np | | | **1.49** | **0.0000** | |
| 9420 | | CYP7B1 | 2.22 | | | 0.0173 | | 1.91 | | 0.0045 | **0.49** | **0.0005** | |
| 9493 | | KIF23 | np | | | | | 1.34 | | 0.0194 | **1.54** | **0.0000** | |
| 9509 | | ADAMTS2 | 1.35 | | | 0.0011 | | 1.10 | | 0.3229 | **1.62** | **0.0000** | |
| 9590 | | AKAP12 | 2.25 | | | 0.0018 | | 2.55 | | 0.0066 | **2.00** | **0.0000** | |
| 9700 | | ESPL1 | 1.24 | | | 0.0000 | | 1.36 | | 0.0004 | **1.54** | **0.0000** | |
| 9833 | | MELK | np | | | | | 1.60 | | 0.0016 | **1.73** | **0.0000** | |
| 9837 | | GINS1 | 1.20 | | | 0.0560 | | 1.37 | | 0.0032 | **1.62** | **0.0000** | |
| 9902 | | MRC2 | 1.22 | | | 0.0429 | | 1.03 | | 0.4804 | **1.41** | **0.0000** | |
| 10112 | | KIF20A | np | | | | | 1.24 | | 0.0166 | **2.18** | **0.0000** | |
| 10212 | | DDX39A | 1.14 | | | 0.2260 | | 1.40 | | 0.0009 | **1.59** | **0.0000** | |
| 10398 | | MYL9 | 0.94 | | | 0.2335 | | 1.08 | | 0.9999 | **1.50** | **0.0000** | |
| 10808 | | HSPH1 | 0.74 | | | 0.0021 | | 0.75 | | 0.0070 | **0.46** | **0.0003** | |
| 10841 | | FTCD | 1.02 | | | 0.9999 | | 1.10 | | 0.2465 | **0.52** | **0.0000** | |
| 10926 | | DBF4 | 1.38 | | | 0.0000 | | 1.41 | | 0.0162 | **1.69** | **0.0000** | |
| 11073 | | TOPBP1 | 1.15 | | | 0.0611 | | 1.21 | | 0.0194 | **1.51** | **0.0000** | |
| 11096 | | ADAMTS5 | 1.23 | | | 0.0004 | | 1.45 | | 0.0051 | **1.46** | **0.0000** | |
| 11270 | | NRM | 1.31 | | | 0.0041 | | 1.55 | | 0.0012 | **1.66** | **0.0000** | |
| 22974 | | TPX2 | np | | | | | np | | | **1.40** | **0.0009** | |
| 23078 | | KIAA0564 | 0.81 | | | 0.0083 | | 0.74 | | 0.0009 | **0.66** | **0.0000** | |
| 23176 | | 41160 | 1.27 | | | 0.0006 | | 1.40 | | 0.0007 | **1.43** | **0.0000** | |
| 23397 | | NCAPH | 1.25 | | | 0.0023 | | 1.27 | | 0.0007 | **1.45** | **0.0007** | |
| 23443 | | SLC35A3 | 0.82 | | | 0.0071 | | 0.96 | | 0.3616 | **0.71** | **0.0000** | |
| 23564 | | DDAH2 | 1.30 | | | 0.0004 | | 1.65 | | 0.0012 | **1.49** | **0.0000** | |
| 23643 | | LY96 | 1.17 | | | 0.2051 | | 1.22 | | 0.0124 | **1.41** | **0.0004** | |
| 25891 | | PAMR1 | 1.31 | | | 0.0000 | | 1.76 | | 0.0144 | **1.65** | **0.0000** | |
| 26059 | | ERC2 | 0.87 | | | 0.0382 | | 0.86 | | 0.1289 | **0.62** | **0.0009** | |
| 26271 | | FBXO5 | 1.25 | | | 0.0128 | | 1.42 | | 0.0109 | **1.44** | **0.0008** | |
| 26586 | | CKAP2 | 1.17 | | | 0.0118 | | 1.12 | | 0.1097 | **1.50** | **0.0004** | |
| 26872 | | STEAP1 | 1.14 | | | 0.4238 | | 1.68 | | 0.0010 | **2.05** | **0.0000** | |
| 27075 | | TSPAN13 | 0.97 | | | 0.2903 | | 0.79 | | 0.0057 | **0.61** | **0.0009** | |
| 27199 | | OXGR1 | 0.64 | | | 0.0013 | | 0.65 | | 0.0014 | **0.63** | **0.0000** | |
| 29028 | | ATAD2 | 1.39 | | | 0.0000 | | 1.37 | | 0.0082 | **1.96** | **0.0003** | |
| 29089 | | UBE2T | np | | | | | 1.28 | | 0.0684 | **1.99** | **0.0003** | |
| 29127 | | RACGAP1 | 1.19 | | | 0.0389 | | 1.37 | | 0.0013 | **1.88** | **0.0000** | |
| 29940 | | DSE | 1.35 | | | 0.0053 | | 1.31 | | 0.0437 | **1.43** | **0.0000** | |
| 51170 | | HSD17B11 | 0.90 | | | 0.1064 | | 0.93 | | 0.1590 | **0.59** | **0.0000** | |
| 51186 | | WBP5 | 1.19 | | | 0.1172 | | 1.34 | | 0.0017 | **1.40** | **0.0000** | |
| 51313 | | FAM198B | 1.21 | | | 0.1064 | | 1.14 | | 0.3754 | **1.69** | **0.0000** | |
| 51440 | | HPCAL4 | 1.26 | | | 0.0341 | | 1.65 | | 0.0232 | **1.60** | **0.0000** | |
| 51752 | | ERAP1 | 1.32 | | | 0.0006 | | 1.70 | | 0.0012 | **1.41** | **0.0000** | |
| 53354 | | PANK1 | 0.96 | | | 0.2665 | | 0.76 | | 0.0025 | **0.59** | **0.0000** | |
| 54677 | | CROT | 0.97 | | | 0.3176 | | 1.02 | | 0.4703 | **0.67** | **0.0000** | |
| 54801 | | HAUS6 | 1.08 | | | 0.2751 | | 1.19 | | 0.1590 | **1.41** | **0.0000** | |
| 54836 | | BSPRY | 1.40 | | | 0.0015 | | 1.68 | | 0.0012 | **1.52** | **0.0000** | |
| 54892 | | NCAPG2 | 1.17 | | | 0.0976 | | 1.34 | | 0.0040 | **1.97** | **0.0000** | |
| 54908 | | CCDC99 | 1.11 | | | 0.1377 | | 1.21 | | 0.0096 | **1.82** | **0.0003** | |
| 54962 | | TIPIN | 1.07 | | | 0.4089 | | 1.03 | | 0.4334 | **1.50** | **0.0000** | |
| 55001 | | TTC22 | 1.03 | | | 0.4194 | | 1.10 | | 0.1745 | **1.40** | **0.0000** | |
| 55022 | | PID1 | 1.36 | | | 0.0075 | | 1.24 | | 0.0684 | **1.51** | **0.0000** | |
| 55076 | | TMEM45A | 1.21 | | | 0.0450 | | 0.88 | | 0.1130 | **1.61** | **0.0000** | |
| 55106 | | SLFN12 | 1.16 | | | 0.1798 | | np | | | **1.49** | **0.0003** | |
| 55143 | | CDCA8 | 1.30 | | | 0.0029 | | 1.38 | | 0.0009 | **1.55** | **0.0003** | |
| 55165 | | CEP55 | 1.38 | | | 0.0145 | | np | | | **1.69** | **0.0003** | |
| 55320 | | MIS18BP1 | 1.26 | | | 0.0216 | | 1.39 | | 0.0032 | **1.74** | **0.0003** | |
| 55466 | | DNAJA4 | 0.86 | | | 0.0029 | | 0.76 | | 0.0006 | **0.61** | **0.0000** | |
| 55723 | | ASF1B | 1.34 | | | 0.0004 | | 1.28 | | 0.0009 | **1.60** | **0.0000** | |
| 55872 | | PBK | 1.39 | | | 0.0368 | | 1.27 | | 0.0421 | **2.92** | **0.0003** | |
| 55917 | | CTTNBP2NL | 1.33 | | | 0.0006 | | 1.41 | | 0.0012 | **1.42** | **0.0000** | |
| 56265 | | CPXM1 | 1.23 | | | 0.0611 | | 1.18 | | 0.2389 | **1.56** | **0.0000** | |
| 57007 | | CXCR7 | 0.78 | | | 0.0023 | | 1.34 | | 0.0058 | **1.43** | **0.0003** | |
| 57127 | | RHBG | 0.71 | | | 0.0023 | | 0.75 | | 0.0129 | **0.71** | **0.0000** | |
| 57447 | | NDRG2 | 0.81 | | | 0.0207 | | 0.75 | | 0.0092 | **0.68** | **0.0000** | |
| 57459 | | GATAD2B | 0.96 | | | 0.3176 | | 0.91 | | 0.2884 | **0.51** | **0.0000** | |
| 57650 | | KIAA1524 | np | | | | | 1.30 | | 0.0421 | **1.84** | **0.0000** | |
| 57715 | | SEMA4G | 0.91 | | | 0.1675 | | 0.76 | | 0.0315 | **0.67** | **0.0000** | |
| 58527 | | ABRACL | 1.20 | | | 0.1227 | | 1.33 | | 0.0011 | **1.44** | **0.0000** | |
| 60681 | | FKBP10 | 1.30 | | | 0.0000 | | 1.13 | | 0.2212 | **1.49** | **0.0000** | |
| 63979 | | FIGNL1 | 1.36 | | | 0.0023 | | 1.53 | | 0.0014 | **2.55** | **0.0000** | |
| 64094 | | SMOC2 | 1.23 | | | 0.0033 | | 0.85 | | 0.2742 | **1.55** | **0.0003** | |
| 64105 | | CENPK | 1.34 | | | 0.0106 | | 1.26 | | 0.0194 | **1.71** | **0.0005** | |
| 64116 | | SLC39A8 | 0.79 | | | 0.0077 | | 0.83 | | 0.0027 | **0.66** | **0.0000** | |
| 64850 | | AGXT2L1 | 0.81 | | | 0.0102 | | 0.72 | | 0.0254 | **0.50** | **0.0005** | |
| 64856 | | VWA1 | 0.76 | | | 0.0186 | | 0.71 | | 0.0242 | **0.59** | **0.0003** | |
| 79188 | | TMEM43 | 1.19 | | | 0.0781 | | 1.30 | | 0.0017 | **1.40** | **0.0000** | |
| 79646 | | PANK3 | 0.78 | | | 0.0017 | | 0.85 | | 0.0212 | **0.70** | **0.0000** | |
| 79784 | | MYH14 | 1.02 | | | 0.9999 | | 0.84 | | 0.0349 | **0.67** | **0.0000** | |
| 79801 | | SHCBP1 | 1.36 | | | 0.1772 | | 1.46 | | 0.0015 | **2.00** | **0.0003** | |
| 79915 | | ATAD5 | 1.31 | | | 0.0150 | | 1.13 | | 0.1534 | **1.58** | **0.0000** | |
| 80146 | | UXS1 | 0.78 | | | 0.0113 | | 1.04 | | 0.4703 | **1.41** | **0.0000** | |
| 80152 | | CENPT | 1.25 | | | 0.0068 | | 1.77 | | 0.0012 | **1.50** | **0.0000** | |
| 80760 | | ITIH5 | 0.91 | | | 0.1947 | | 0.97 | | 0.4553 | **0.57** | **0.0000** | |
| 80896 | | NPL | 0.67 | | | 0.0013 | | 0.89 | | 0.2327 | **2.01** | **0.0000** | |
| 81620 | | CDT1 | 1.20 | | | 0.0521 | | 1.11 | | 0.1163 | **1.75** | **0.0000** | |
| 81788 | | NUAK2 | 1.22 | | | 0.1976 | | 1.21 | | 0.1289 | **1.90** | **0.0000** | |
| 83752 | | LONP2 | 0.81 | | | 0.0006 | | 0.77 | | 0.0004 | **0.68** | **0.0000** | |
| 83953 | | FCAMR | 0.93 | | | 0.1843 | | 0.85 | | 0.0754 | **0.56** | **0.0003** | |
| 84034 | | EMILIN2 | 1.40 | | | 0.0004 | | 1.03 | | 0.3754 | **1.42** | **0.0000** | |
| 84225 | | ZMYND15 | 1.29 | | | 0.0008 | | 1.60 | | 0.0016 | **1.56** | **0.0000** | |
| 84752 | | B3GNT9 | 1.21 | | | 0.0429 | | np | | | **1.41** | **0.0003** | |
| 84804 | | MFSD9 | 0.76 | | | 0.0194 | | 0.80 | | 0.0166 | **0.67** | **0.0005** | |
| 84830 | | ADTRP | 0.74 | | | 0.0044 | | 0.74 | | 0.0203 | **0.61** | **0.0000** | |
| 89796 | | NAV1 | 1.41 | | | 0.0014 | | 1.27 | | 0.1097 | **1.59** | **0.0000** | |
| 90362 | | FAM110B | 1.09 | | | 0.2945 | | 1.38 | | 0.0184 | **1.41** | **0.0005** | |
| 90417 | | C15orf23 | np | | | | | 1.19 | | 0.0016 | **1.42** | **0.0000** | |
| 91373 | | UAP1L1 | 1.07 | | | 0.4217 | | 1.18 | | 0.1130 | **1.45** | **0.0000** | |
| 91663 | | MYADM | 1.34 | | | 0.0006 | | 1.37 | | 0.0194 | **1.54** | **0.0000** | |
| 91694 | | LONRF1 | 0.67 | | | 0.0013 | | 0.68 | | 0.0010 | **0.66** | **0.0003** | |
| 91851 | | CHRDL1 | 0.89 | | | 0.0678 | | 0.75 | | 0.0075 | **0.71** | **0.0003** | |
| 113130 | | CDCA5 | 1.26 | | | 0.0186 | | 1.61 | | 0.0017 | **2.09** | **0.0000** | |
| 114789 | | SLC25A25 | 0.72 | | | 0.0100 | | 0.85 | | 0.1289 | **0.39** | **0.0000** | |
| 116255 | | MOGAT1 | 0.86 | | | 0.1083 | | 1.31 | | 0.0782 | **0.71** | **0.0000** | |
| 123036 | | TC2N | np | | | | | np | | | **0.64** | **0.0000** | |
| 124599 | | CD300LB | 1.49 | | | 0.0060 | | np | | | **1.49** | **0.0004** | |
| 124935 | | SLC43A2 | 0.75 | | | 0.0004 | | 0.61 | | 0.0015 | **0.67** | **0.0000** | |
| 126129 | | CPT1C | 1.20 | | | 0.0258 | | 1.64 | | 0.0035 | **1.52** | **0.0000** | |
| 128853 | | DUSP15 | 0.73 | | | 0.0000 | | 0.64 | | 0.0012 | **0.70** | **0.0002** | |
| 134526 | | ACOT12 | 0.89 | | | 0.1150 | | 0.81 | | 0.1163 | **0.57** | **0.0005** | |
| 138065 | | RNF183 | 0.91 | | | 0.1531 | | 1.08 | | 0.3826 | **1.57** | **0.0000** | |
| 138255 | | C9orf135 | np | | | | | np | | | **0.53** | **0.0003** | |
| 139065 | | SLITRK4 | np | | | | | 2.65 | | 0.0070 | **1.82** | **0.0005** | |
| 140456 | | ASB11 | 0.99 | | | 0.3591 | | 0.87 | | 0.1017 | **0.60** | **0.0000** | |
| 140885 | | SIRPA | 1.19 | | | 0.2710 | | 1.06 | | 0.3296 | **1.54** | **0.0000** | |
| 143888 | | KDELC2 | 1.05 | | | 0.4138 | | 1.26 | | 0.0036 | **1.46** | **0.0000** | |
| 144811 | | LACC1 | 1.25 | | | 0.0019 | | 1.24 | | 0.0004 | **1.51** | **0.0005** | |
| 147111 | | NOTUM | 0.75 | | | 0.0008 | | 0.59 | | 0.0014 | **0.67** | **0.0000** | |
| 147841 | | SPC24 | 1.20 | | | 0.0023 | | 1.25 | | 0.0133 | **1.46** | **0.0003** | |
| 151246 | | SGOL2 | 1.15 | | | 0.0232 | | 1.32 | | 0.0006 | **1.60** | **0.0000** | |
| 151648 | | SGOL1 | 1.12 | | | 0.0727 | | 1.36 | | 0.0005 | **1.51** | **0.0003** | |
| 157570 | | ESCO2 | np | | | | | np | | | **2.39** | **0.0000** | |
| 158326 | | FREM1 | 1.17 | | | 0.1035 | | 0.90 | | 0.3229 | **1.95** | **0.0002** | |
| 160851 | | DGKH | 0.92 | | | 0.1172 | | 1.08 | | 0.2954 | **0.70** | **0.0000** | |
| 196740 | | VSTM4 | 1.30 | | | 0.0450 | | 0.95 | | 0.4009 | **1.52** | **0.0000** | |
| 201725 | | C4orf46 | 1.14 | | | 0.0579 | | 1.22 | | 0.1341 | **1.68** | **0.0000** | |
| 220042 | | C11orf82 | np | | | | | 1.36 | | 0.0077 | **1.50** | **0.0000** | |
| 255027 | | MPV17L | 0.87 | | | 0.0880 | | 0.68 | | 0.0082 | **0.66** | **0.0000** | |
| 261729 | | STEAP2 | 0.85 | | | 0.0505 | | 1.03 | | 0.4151 | **1.42** | **0.0000** | |
| 283431 | | GAS2L3 | np | | | | | 1.22 | | 0.0511 | **1.51** | **0.0000** | |
| 285172 | | FAM126B | 0.74 | | | 0.0004 | | 0.79 | | 0.0151 | **0.70** | **0.0003** | |
| 353189 | | SLCO4C1 | 1.16 | | | 0.1906 | | 0.58 | | 0.0032 | **0.59** | **0.0005** | |
| 375790 | | AGRN | 1.25 | | | 0.0091 | | 1.68 | | 0.0012 | **1.41** | **0.0000** | |
| 387509 | | GPR153 | 1.19 | | | 0.1408 | | 1.17 | | 0.1795 | **1.41** | **0.0000** | |
| 389136 | | VGLL3 | 1.27 | | | 0.0004 | | 1.48 | | 0.0139 | **1.49** | **0.0004** | |
| 401541 | | CENPP | np | | | | | 1.24 | | 0.0170 | **1.55** | **0.0000** | |
| 440498 | | HSBP1L1 | 0.78 | | | 0.0290 | | 0.53 | | 0.0012 | **0.65** | **0.0000** | |
| 727936 | | GXYLT2 | np | | | | | np | | | **1.55** | **0.0000** | |
| 277 | | AMY1B | 0.91 | | | 0.1843 | | 0.60 | | 0.0474 | **0.57** | **0.0007** | |
| 277 | | AMY1B | 0.91 | | | 0.1843 | | 0.60 | | 0.0474 | **0.57** | **0.0007** | |
| 277 | | AMY1B | 0.91 | | | 0.1843 | | 0.60 | | 0.0474 | **0.57** | **0.0007** | |
| 2953 | | GSTT2 | 0.88 | | | 0.0976 | | 0.77 | | 0.0005 | **0.71** | **0.0005** | |
| **D. 263 genes regulated in the same direction in NZB/W, NZM2410 and NZW/BXSB.** | | | | | | | | | | | | | |
| Human Entrez Gene ID | | Human Gene symbol | **NZB/W** | | | | | **NZM2410** | | | **NZW/BXSB** | | |
|  |  |  | Fold-change | | | q-value | | Fold-change | | q-value | Fold-change | q-value | |
| 18 | | ABAT | **0.50** | | | **0.0000** | | **0.42** | | **0.0004** | **0.68** | **0.0000** | |
| 5826 | | ABCD4 | **0.70** | | | **0.0004** | | **0.50** | | **0.0000** | **0.69** | **0.0005** | |
| 26 | | ABP1 | **2.28** | | | **0.0004** | | **9.87** | | **0.0000** | **2.64** | **0.0000** | |
| 28976 | | ACAD9 | **0.65** | | | **0.0000** | | **0.55** | | **0.0005** | **0.59** | **0.0000** | |
| 38 | | ACAT1 | **0.67** | | | **0.0004** | | **0.54** | | **0.0000** | **0.71** | **0.0007** | |
| 116285 | | ACSM1 | **0.60** | | | **0.0006** | | **0.34** | | **0.0000** | **0.51** | **0.0000** | |
| 54988 | | ACSM5 | **0.60** | | | **0.0004** | | **0.46** | | **0.0000** | **0.54** | **0.0000** | |
| 55902 | | ACSS2 | **0.64** | | | **0.0004** | | **0.49** | | **0.0000** | **0.63** | **0.0000** | |
| 87 | | ACTN1 | **1.54** | | | **0.0000** | | **2.00** | | **0.0006** | **1.69** | **0.0000** | |
| 137872 | | ADHFE1 | **0.71** | | | **0.0003** | | **0.57** | | **0.0000** | **0.70** | **0.0000** | |
| 122622 | | ADSSL1 | **0.58** | | | **0.0000** | | **0.39** | | **0.0000** | **0.60** | **0.0000** | |
| 173 | | AFM | **0.51** | | | **0.0000** | | **0.36** | | **0.0000** | **0.59** | **0.0000** | |
| 125061 | | AFMID | **0.58** | | | **0.0000** | | **0.45** | | **0.0000** | **0.63** | **0.0000** | |
| 199 | | AIF1 | **3.48** | | | **0.0000** | | **2.56** | | **0.0004** | **2.02** | **0.0000** | |
| 57016 | | AKR1B10 | **1.48** | | | **0.0000** | | **3.86** | | **0.0000** | **1.96** | **0.0000** | |
| 6718 | | AKR1D1 | **0.46** | | | **0.0000** | | **0.22** | | **0.0006** | **0.52** | **0.0000** | |
| 5832 | | ALDH18A1 | **1.49** | | | **0.0000** | | **1.59** | | **0.0004** | **1.54** | **0.0000** | |
| 8854 | | ALDH1A2 | **1.98** | | | **0.0000** | | **2.16** | | **0.0000** | **3.42** | **0.0000** | |
| 241 | | ALOX5AP | **2.16** | | | **0.0000** | | **2.23** | | **0.0000** | **1.91** | **0.0000** | |
| 27063 | | ANKRD1 | **3.16** | | | **0.0000** | | **7.33** | | **0.0000** | **2.09** | **0.0000** | |
| 301 | | ANXA1 | **2.20** | | | **0.0000** | | **3.59** | | **0.0000** | **2.48** | **0.0000** | |
| 302 | | ANXA2 | **1.84** | | | **0.0000** | | **3.51** | | **0.0000** | **2.08** | **0.0000** | |
| 306 | | ANXA3 | **2.31** | | | **0.0000** | | **6.10** | | **0.0000** | **2.24** | **0.0000** | |
| 339 | | APOBEC1 | **2.13** | | | **0.0000** | | **2.54** | | **0.0004** | **1.82** | **0.0000** | |
| 348 | | APOE | **2.10** | | | **0.0000** | | **2.19** | | **0.0000** | **2.03** | **0.0003** | |
| 282679 | | AQP11 | **0.51** | | | **0.0000** | | **0.48** | | **0.0004** | **0.68** | **0.0000** | |
| 361 | | AQP4 | **0.40** | | | **0.0000** | | **0.35** | | **0.0006** | **0.60** | **0.0003** | |
| 10123 | | ARL4C | **1.50** | | | **0.0000** | | **1.86** | | **0.0000** | **1.43** | **0.0000** | |
| 440 | | ASNS | **1.74** | | | **0.0000** | | **2.39** | | **0.0000** | **1.78** | **0.0000** | |
| 443 | | ASPA | **0.64** | | | **0.0006** | | **0.51** | | **0.0000** | **0.67** | **0.0000** | |
| 554235 | | ASPDH | **0.54** | | | **0.0004** | | **0.34** | | **0.0005** | **0.52** | **0.0005** | |
| 558 | | AXL | **2.14** | | | **0.0000** | | **1.70** | | **0.0000** | **1.71** | **0.0000** | |
| 567 | | B2M | **1.53** | | | **0.0000** | | **2.57** | | **0.0000** | **1.47** | **0.0000** | |
| 2583 | | B4GALNT1 | **2.65** | | | **0.0000** | | **3.67** | | **0.0004** | **1.79** | **0.0000** | |
| 587 | | BCAT2 | **0.64** | | | **0.0006** | | **0.53** | | **0.0000** | **0.70** | **0.0000** | |
| 602 | | BCL3 | **2.17** | | | **0.0000** | | **2.53** | | **0.0000** | **2.07** | **0.0000** | |
| 83875 | | BCO2 | **0.65** | | | **0.0000** | | **0.46** | | **0.0005** | **0.64** | **0.0003** | |
| 330 | | BIRC3 | **1.89** | | | **0.0000** | | **2.49** | | **0.0000** | **1.78** | **0.0000** | |
| 332 | | BIRC5 | **2.11** | | | **0.0000** | | **2.00** | | **0.0000** | **3.23** | **0.0000** | |
| 670 | | BPHL | **0.66** | | | **0.0000** | | **0.60** | | **0.0005** | **0.69** | **0.0000** | |
| 684 | | BST2 | **2.07** | | | **0.0000** | | **2.42** | | **0.0000** | **1.79** | **0.0000** | |
| 219621 | | C10orf107 | **0.57** | | | **0.0000** | | **0.66** | | **0.0000** | **0.69** | **0.0000** | |
| 712 | | C1QA | **4.87** | | | **0.0000** | | **3.51** | | **0.0000** | **2.70** | **0.0000** | |
| 713 | | C1QB | **6.51** | | | **0.0000** | | **3.83** | | **0.0000** | **2.89** | **0.0000** | |
| 714 | | C1QC | **6.52** | | | **0.0000** | | **3.52** | | **0.0000** | **2.87** | **0.0000** | |
| 718 | | C3 | **4.16** | | | **0.0000** | | **7.57** | | **0.0004** | **5.47** | **0.0000** | |
| 719 | | C3AR1 | **2.16** | | | **0.0000** | | **2.64** | | **0.0005** | **2.21** | **0.0000** | |
| 23632 | | CA14 | **0.43** | | | **0.0000** | | **0.25** | | **0.0000** | **0.66** | **0.0006** | |
| 793 | | CALB1 | **0.45** | | | **0.0000** | | **0.29** | | **0.0000** | **0.50** | **0.0000** | |
| 837 | | CASP4 | **2.05** | | | **0.0000** | | **3.39** | | **0.0004** | **1.92** | **0.0000** | |
| 6357 | | CCL13 | **1.78** | | | **0.0007** | | **2.83** | | **0.0000** | **2.02** | **0.0000** | |
| 6347 | | CCL2 | **3.45** | | | **0.0000** | | **3.11** | | **0.0000** | **3.71** | **0.0003** | |
| 6352 | | CCL5 | **3.27** | | | **0.0000** | | **10.59** | | **0.0008** | **2.70** | **0.0000** | |
| 9133 | | CCNB2 | **1.79** | | | **0.0004** | | **1.80** | | **0.0000** | **2.86** | **0.0003** | |
| 729230 | | CCR2 | **2.18** | | | **0.0000** | | **3.54** | | **0.0006** | **2.54** | **0.0000** | |
| 1234 | | CCR5 | **1.84** | | | **0.0000** | | **1.93** | | **0.0000** | **2.08** | **0.0000** | |
| 929 | | CD14 | **4.19** | | | **0.0000** | | **9.53** | | **0.0000** | **3.08** | **0.0000** | |
| 960 | | CD44 | **4.57** | | | **0.0000** | | **14.60** | | **0.0000** | **3.51** | **0.0000** | |
| 963 | | CD53 | **5.90** | | | **0.0000** | | **3.32** | | **0.0005** | **2.39** | **0.0000** | |
| 968 | | CD68 | **3.37** | | | **0.0000** | | **4.75** | | **0.0000** | **2.44** | **0.0000** | |
| 971 | | CD72 | **4.10** | | | **0.0000** | | **1.88** | | **0.0009** | **2.42** | **0.0000** | |
| 972 | | CD74 | **3.27** | | | **0.0000** | | **5.96** | | **0.0000** | **1.86** | **0.0000** | |
| 8832 | | CD84 | **1.84** | | | **0.0000** | | **1.73** | | **0.0000** | **1.50** | **0.0000** | |
| 991 | | CDC20 | **2.28** | | | **0.0000** | | **1.46** | | **0.0000** | **3.85** | **0.0003** | |
| 983 | | CDK1 | **1.77** | | | **0.0004** | | **1.66** | | **0.0000** | **3.05** | **0.0000** | |
| 494143 | | CHAC2 | **0.62** | | | **0.0006** | | **0.62** | | **0.0000** | **0.70** | **0.0009** | |
| 1140 | | CHRNB1 | **1.78** | | | **0.0000** | | **3.37** | | **0.0006** | **2.13** | **0.0000** | |
| 1163 | | CKS1B | **2.17** | | | **0.0000** | | **2.24** | | **0.0000** | **2.31** | **0.0000** | |
| 1364 | | CLDN4 | **1.97** | | | **0.0000** | | **4.31** | | **0.0000** | **1.81** | **0.0000** | |
| 50856 | | CLEC4A | **2.99** | | | **0.0000** | | **1.86** | | **0.0000** | **1.66** | **0.0000** | |
| 1192 | | CLIC1 | **1.51** | | | **0.0000** | | **1.92** | | **0.0000** | **1.47** | **0.0000** | |
| 134147 | | CMBL | **0.54** | | | **0.0000** | | **0.39** | | **0.0000** | **0.66** | **0.0000** | |
| 123920 | | CMTM3 | **1.98** | | | **0.0000** | | **2.67** | | **0.0004** | **1.77** | **0.0000** | |
| 84735 | | CNDP1 | **0.44** | | | **0.0000** | | **0.23** | | **0.0000** | **0.49** | **0.0000** | |
| 11151 | | CORO1A | **5.26** | | | **0.0000** | | **2.93** | | **0.0000** | **2.44** | **0.0000** | |
| 1356 | | CP | **4.26** | | | **0.0000** | | **8.94** | | **0.0000** | **2.73** | **0.0000** | |
| 1396 | | CRIP1 | **1.88** | | | **0.0000** | | **1.63** | | **0.0009** | **1.59** | **0.0000** | |
| 64109 | | CRLF2 | **1.41** | | | **0.0000** | | **1.74** | | **0.0000** | **1.40** | **0.0000** | |
| 1438 | | CSF2RA | **1.84** | | | **0.0000** | | **1.63** | | **0.0009** | **1.56** | **0.0000** | |
| 1075 | | CTSC | **2.67** | | | **0.0000** | | **3.39** | | **0.0000** | **2.17** | **0.0000** | |
| 1509 | | CTSD | **1.56** | | | **0.0000** | | **2.44** | | **0.0000** | **1.57** | **0.0000** | |
| 1520 | | CTSS | **5.22** | | | **0.0000** | | **4.71** | | **0.0000** | **2.61** | **0.0000** | |
| 1524 | | CX3CR1 | **1.59** | | | **0.0006** | | **2.24** | | **0.0000** | **1.63** | **0.0000** | |
| 3627 | | CXCL10 | **2.44** | | | **0.0004** | | **4.36** | | **0.0000** | **3.58** | **0.0006** | |
| 58191 | | CXCL16 | **1.69** | | | **0.0000** | | **2.23** | | **0.0000** | **1.98** | **0.0000** | |
| 1536 | | CYBB | **3.49** | | | **0.0000** | | **2.79** | | **0.0000** | **2.30** | **0.0000** | |
| 114757 | | CYGB | **2.06** | | | **0.0000** | | **2.43** | | **0.0006** | **1.73** | **0.0000** | |
| 1629 | | DBT | **0.67** | | | **0.0008** | | **0.59** | | **0.0005** | **0.70** | **0.0000** | |
| 84649 | | DGAT2 | **0.65** | | | **0.0000** | | **0.52** | | **0.0000** | **0.71** | **0.0000** | |
| 1733 | | DIO1 | **0.46** | | | **0.0000** | | **0.30** | | **0.0004** | **0.49** | **0.0000** | |
| 29958 | | DMGDH | **0.69** | | | **0.0004** | | **0.46** | | **0.0006** | **0.67** | **0.0000** | |
| 1773 | | DNASE1 | **0.36** | | | **0.0000** | | **0.11** | | **0.0000** | **0.32** | **0.0000** | |
| 55619 | | DOCK10 | **1.65** | | | **0.0000** | | **2.68** | | **0.0000** | **1.45** | **0.0000** | |
| 139818 | | DOCK11 | **2.56** | | | **0.0000** | | **1.71** | | **0.0009** | **1.61** | **0.0000** | |
| 1794 | | DOCK2 | **1.63** | | | **0.0000** | | **1.53** | | **0.0008** | **1.46** | **0.0000** | |
| 151636 | | DTX3L | **1.96** | | | **0.0000** | | **2.46** | | **0.0006** | **1.56** | **0.0003** | |
| 79180 | | EFHD2 | **2.31** | | | **0.0000** | | **3.04** | | **0.0000** | **1.88** | **0.0000** | |
| 2015 | | EMR1 | **3.57** | | | **0.0000** | | **3.39** | | **0.0000** | **2.11** | **0.0000** | |
| 23344 | | ESYT1 | **1.56** | | | **0.0000** | | **1.52** | | **0.0004** | **1.45** | **0.0000** | |
| 2123 | | EVI2A | **3.47** | | | **0.0000** | | **2.77** | | **0.0009** | **2.10** | **0.0004** | |
| 2165 | | F13B | **0.50** | | | **0.0004** | | **0.36** | | **0.0005** | **0.63** | **0.0006** | |
| 2149 | | F2R | **2.50** | | | **0.0000** | | **2.40** | | **0.0005** | **2.11** | **0.0002** | |
| 63901 | | FAM111A | **1.80** | | | **0.0000** | | **1.55** | | **0.0005** | **1.70** | **0.0000** | |
| 116496 | | FAM129A | **1.81** | | | **0.0000** | | **2.92** | | **0.0000** | **1.80** | **0.0000** | |
| 84331 | | FAM195A | **0.69** | | | **0.0008** | | **0.53** | | **0.0000** | **0.71** | **0.0000** | |
| 2195 | | FAT1 | **1.55** | | | **0.0004** | | **1.95** | | **0.0007** | **1.60** | **0.0000** | |
| 54751 | | FBLIM1 | **1.59** | | | **0.0000** | | **1.73** | | **0.0000** | **1.64** | **0.0000** | |
| 2207 | | FCER1G | **2.36** | | | **0.0000** | | **1.93** | | **0.0000** | **2.01** | **0.0000** | |
| 2209 | | FCGR1A | **1.52** | | | **0.0000** | | **1.75** | | **0.0000** | **1.51** | **0.0000** | |
| 2212 | | FCGR2A | **3.90** | | | **0.0000** | | **3.47** | | **0.0000** | **2.61** | **0.0000** | |
| 83706 | | FERMT3 | **3.03** | | | **0.0000** | | **2.24** | | **0.0005** | **1.81** | **0.0000** | |
| 2266 | | FGG | **4.25** | | | **0.0000** | | **6.14** | | **0.0000** | **2.74** | **0.0003** | |
| 2316 | | FLNA | **1.70** | | | **0.0000** | | **2.17** | | **0.0005** | **1.98** | **0.0000** | |
| 2327 | | FMO2 | **0.69** | | | **0.0006** | | **0.62** | | **0.0008** | **0.71** | **0.0009** | |
| 2358 | | FPR2 | **5.48** | | | **0.0000** | | **2.88** | | **0.0000** | **2.51** | **0.0000** | |
| 2533 | | FYB | **2.30** | | | **0.0000** | | **1.75** | | **0.0008** | **1.48** | **0.0000** | |
| 51280 | | GOLM1 | **1.67** | | | **0.0000** | | **3.11** | | **0.0000** | **2.24** | **0.0000** | |
| 257202 | | GPX6 | **0.55** | | | **0.0000** | | **0.44** | | **0.0006** | **0.42** | **0.0000** | |
| 2998 | | GYS2 | **0.56** | | | **0.0000** | | **0.35** | | **0.0005** | **0.61** | **0.0000** | |
| 26762 | | HAVCR1 | **2.58** | | | **0.0004** | | **51.42** | | **0.0000** | **2.43** | **0.0000** | |
| 3055 | | HCK | **3.76** | | | **0.0000** | | **2.29** | | **0.0000** | **2.15** | **0.0000** | |
| 3059 | | HCLS1 | **2.41** | | | **0.0000** | | **1.90** | | **0.0006** | **1.69** | **0.0000** | |
| 8820 | | HESX1 | **1.51** | | | **0.0006** | | **4.39** | | **0.0000** | **1.74** | **0.0000** | |
| 3107 | | HLA-C | **2.91** | | | **0.0000** | | **5.63** | | **0.0000** | **2.10** | **0.0000** | |
| 3108 | | HLA-DMA | **2.09** | | | **0.0000** | | **3.22** | | **0.0000** | **1.90** | **0.0000** | |
| 3109 | | HLA-DMB | **2.39** | | | **0.0000** | | **7.13** | | **0.0000** | **1.78** | **0.0000** | |
| 3119 | | HLA-DQB1 | **2.83** | | | **0.0000** | | **6.30** | | **0.0000** | **1.93** | **0.0000** | |
| 3242 | | HPD | **0.45** | | | **0.0008** | | **0.16** | | **0.0000** | **0.21** | **0.0005** | |
| 3383 | | ICAM1 | **2.59** | | | **0.0000** | | **3.78** | | **0.0000** | **1.70** | **0.0000** | |
| 3430 | | IFI35 | **1.71** | | | **0.0000** | | **1.82** | | **0.0009** | **1.46** | **0.0000** | |
| 10581 | | IFITM2 | **1.96** | | | **0.0000** | | **2.95** | | **0.0000** | **1.83** | **0.0000** | |
| 10261 | | IGSF6 | **1.75** | | | **0.0000** | | **1.66** | | **0.0005** | **1.51** | **0.0000** | |
| 146433 | | IL34 | **1.74** | | | **0.0000** | | **3.85** | | **0.0000** | **1.90** | **0.0000** | |
| 27179 | | IL36A | **1.95** | | | **0.0004** | | **3.71** | | **0.0000** | **2.47** | **0.0000** | |
| 3659 | | IRF1 | **1.66** | | | **0.0000** | | **2.13** | | **0.0000** | **1.47** | **0.0000** | |
| 3665 | | IRF7 | **2.47** | | | **0.0000** | | **2.80** | | **0.0009** | **2.41** | **0.0000** | |
| 10379 | | IRF9 | **1.97** | | | **0.0000** | | **2.33** | | **0.0000** | **1.51** | **0.0000** | |
| 345611 | | IRGM | **2.20** | | | **0.0000** | | **2.56** | | **0.0006** | **2.23** | **0.0000** | |
| 3875 | | KRT18 | **1.90** | | | **0.0000** | | **2.63** | | **0.0008** | **1.52** | **0.0009** | |
| 7805 | | LAPTM5 | **3.05** | | | **0.0000** | | **2.45** | | **0.0000** | **1.83** | **0.0000** | |
| 3934 | | LCN2 | **21.72** | | | **0.0000** | | **43.71** | | **0.0000** | **22.84** | **0.0000** | |
| 3956 | | LGALS1 | **1.50** | | | **0.0007** | | **3.09** | | **0.0000** | **1.89** | **0.0000** | |
| 3958 | | LGALS3 | **1.95** | | | **0.0000** | | **3.63** | | **0.0000** | **3.10** | **0.0000** | |
| 3959 | | LGALS3BP | **2.62** | | | **0.0000** | | **2.78** | | **0.0000** | **2.19** | **0.0000** | |
| 11025 | | LILRB3 | **2.72** | | | **0.0000** | | **1.86** | | **0.0005** | **1.47** | **0.0000** | |
| 9516 | | LITAF | **1.62** | | | **0.0004** | | **2.21** | | **0.0000** | **1.79** | **0.0000** | |
| 9404 | | LPXN | **2.93** | | | **0.0000** | | **2.09** | | **0.0006** | **1.85** | **0.0000** | |
| 116844 | | LRG1 | **4.24** | | | **0.0000** | | **4.12** | | **0.0005** | **1.83** | **0.0003** | |
| 440699 | | LRRC52 | **0.69** | | | **0.0000** | | **0.42** | | **0.0000** | **0.69** | **0.0004** | |
| 339977 | | LRRC66 | **0.58** | | | **0.0000** | | **0.39** | | **0.0000** | **0.63** | **0.0005** | |
| 4046 | | LSP1 | **2.44** | | | **0.0000** | | **1.65** | | **0.0009** | **1.45** | **0.0000** | |
| 4053 | | LTBP2 | **1.88** | | | **0.0000** | | **2.82** | | **0.0005** | **1.79** | **0.0000** | |
| 4061 | | LY6E | **2.67** | | | **0.0000** | | **4.51** | | **0.0000** | **2.34** | **0.0000** | |
| 9450 | | LY86 | **2.86** | | | **0.0000** | | **2.46** | | **0.0000** | **2.22** | **0.0000** | |
| 4066 | | LYL1 | **1.55** | | | **0.0000** | | **1.75** | | **0.0009** | **1.40** | **0.0000** | |
| 4085 | | MAD2L1 | **1.45** | | | **0.0006** | | **1.47** | | **0.0006** | **1.62** | **0.0000** | |
| 65108 | | MARCKSL1 | **2.05** | | | **0.0000** | | **1.77** | | **0.0004** | **1.57** | **0.0003** | |
| 56922 | | MCCC1 | **0.62** | | | **0.0000** | | **0.62** | | **0.0006** | **0.71** | **0.0000** | |
| 4323 | | MMP14 | **1.64** | | | **0.0000** | | **1.86** | | **0.0007** | **1.68** | **0.0000** | |
| 219972 | | MPEG1 | **3.42** | | | **0.0000** | | **2.96** | | **0.0004** | **2.00** | **0.0000** | |
| 4360 | | MRC1 | **2.66** | | | **0.0000** | | **3.27** | | **0.0005** | **2.31** | **0.0000** | |
| 64231 | | MS4A6A | **6.69** | | | **0.0000** | | **3.58** | | **0.0000** | **3.02** | **0.0000** | |
| 58475 | | MS4A7 | **6.38** | | | **0.0000** | | **3.71** | | **0.0004** | **3.03** | **0.0000** | |
| 4496 | | MT1H | **2.86** | | | **0.0000** | | **4.85** | | **0.0000** | **3.06** | **0.0000** | |
| 10797 | | MTHFD2 | **1.49** | | | **0.0000** | | **1.82** | | **0.0004** | **1.76** | **0.0000** | |
| 9961 | | MVP | **1.49** | | | **0.0000** | | **2.05** | | **0.0000** | **1.62** | **0.0000** | |
| 439921 | | MXRA7 | **1.41** | | | **0.0000** | | **3.91** | | **0.0000** | **1.65** | **0.0005** | |
| 133686 | | NADKD1 | **0.63** | | | **0.0000** | | **0.63** | | **0.0004** | **0.69** | **0.0000** | |
| 4689 | | NCF4 | **2.64** | | | **0.0000** | | **2.00** | | **0.0007** | **1.63** | **0.0000** | |
| 3071 | | NCKAP1L | **1.78** | | | **0.0000** | | **1.81** | | **0.0009** | **1.70** | **0.0000** | |
| 4794 | | NFKBIE | **2.30** | | | **0.0000** | | **3.78** | | **0.0000** | **1.81** | **0.0000** | |
| 64332 | | NFKBIZ | **2.19** | | | **0.0000** | | **3.84** | | **0.0000** | **1.71** | **0.0000** | |
| 390916 | | NUDT19 | **0.55** | | | **0.0000** | | **0.38** | | **0.0000** | **0.44** | **0.0000** | |
| 51203 | | NUSAP1 | **1.80** | | | **0.0009** | | **1.48** | | **0.0000** | **2.36** | **0.0000** | |
| 9180 | | OSMR | **2.14** | | | **0.0000** | | **3.02** | | **0.0000** | **1.74** | **0.0000** | |
| 5031 | | P2RY6 | **2.48** | | | **0.0000** | | **2.82** | | **0.0000** | **2.16** | **0.0000** | |
| 64761 | | PARP12 | **1.79** | | | **0.0000** | | **2.13** | | **0.0004** | **1.77** | **0.0000** | |
| 54625 | | PARP14 | **2.20** | | | **0.0000** | | **2.84** | | **0.0003** | **1.70** | **0.0000** | |
| 83666 | | PARP9 | **1.85** | | | **0.0000** | | **2.54** | | **0.0004** | **1.75** | **0.0000** | |
| 64081 | | PBLD | **0.57** | | | **0.0000** | | **0.44** | | **0.0000** | **0.59** | **0.0000** | |
| 5164 | | PDK2 | **0.61** | | | **0.0000** | | **0.49** | | **0.0000** | **0.66** | **0.0000** | |
| 8682 | | PEA15 | **1.88** | | | **0.0000** | | **2.81** | | **0.0000** | **2.13** | **0.0000** | |
| 118788 | | PIK3AP1 | **2.63** | | | **0.0000** | | **2.51** | | **0.0000** | **1.81** | **0.0000** | |
| 51316 | | PLAC8 | **2.81** | | | **0.0000** | | **3.76** | | **0.0000** | **2.55** | **0.0000** | |
| 122618 | | PLD4 | **2.75** | | | **0.0000** | | **2.45** | | **0.0000** | **1.97** | **0.0000** | |
| 5341 | | PLEK | **3.05** | | | **0.0000** | | **2.36** | | **0.0009** | **1.91** | **0.0000** | |
| 51177 | | PLEKHO1 | **1.82** | | | **0.0000** | | **1.91** | | **0.0000** | **1.62** | **0.0000** | |
| 84152 | | PPP1R1B | **0.67** | | | **0.0000** | | **0.56** | | **0.0007** | **0.66** | **0.0000** | |
| 5579 | | PRKCB | **1.95** | | | **0.0000** | | **1.66** | | **0.0004** | **1.51** | **0.0000** | |
| 5699 | | PSMB10 | **1.96** | | | **0.0000** | | **2.43** | | **0.0000** | **1.74** | **0.0000** | |
| 5696 | | PSMB8 | **3.17** | | | **0.0000** | | **4.35** | | **0.0004** | **2.06** | **0.0000** | |
| 145482 | | PTGR2 | **0.64** | | | **0.0000** | | **0.59** | | **0.0000** | **0.70** | **0.0000** | |
| 5788 | | PTPRC | **6.03** | | | **0.0000** | | **3.06** | | **0.0005** | **2.47** | **0.0000** | |
| 5791 | | PTPRE | **1.72** | | | **0.0000** | | **1.62** | | **0.0005** | **1.41** | **0.0000** | |
| 5816 | | PVALB | **0.44** | | | **0.0004** | | **0.17** | | **0.0004** | **0.30** | **0.0000** | |
| 29108 | | PYCARD | **1.97** | | | **0.0000** | | **1.53** | | **0.0004** | **1.87** | **0.0000** | |
| 11031 | | RAB31 | **1.47** | | | **0.0000** | | **1.81** | | **0.0000** | **1.71** | **0.0000** | |
| 5954 | | RCN1 | **1.72** | | | **0.0000** | | **2.27** | | **0.0007** | **1.85** | **0.0000** | |
| 5959 | | RDH5 | **0.68** | | | **0.0000** | | **0.45** | | **0.0000** | **0.70** | **0.0003** | |
| 5971 | | RELB | **1.80** | | | **0.0000** | | **3.11** | | **0.0000** | **1.78** | **0.0000** | |
| 768211 | | RELL1 | **1.95** | | | **0.0000** | | **2.26** | | **0.0008** | **1.44** | **0.0003** | |
| 10287 | | RGS19 | **1.88** | | | **0.0000** | | **2.08** | | **0.0009** | **1.75** | **0.0000** | |
| 388 | | RHOB | **1.59** | | | **0.0004** | | **2.64** | | **0.0000** | **1.52** | **0.0003** | |
| 58480 | | RHOU | **1.58** | | | **0.0006** | | **3.14** | | **0.0004** | **1.46** | **0.0000** | |
| 6237 | | RRAS | **1.65** | | | **0.0000** | | **2.65** | | **0.0000** | **1.64** | **0.0000** | |
| 64108 | | RTP4 | **1.96** | | | **0.0000** | | **2.52** | | **0.0004** | **1.88** | **0.0003** | |
| 6277 | | S100A6 | **2.06** | | | **0.0000** | | **2.23** | | **0.0000** | **2.13** | **0.0000** | |
| 1757 | | SARDH | **0.70** | | | **0.0000** | | **0.50** | | **0.0000** | **0.66** | **0.0000** | |
| 6382 | | SDC1 | **1.49** | | | **0.0000** | | **1.99** | | **0.0000** | **1.42** | **0.0000** | |
| 10512 | | SEMA3C | **1.69** | | | **0.0004** | | **3.42** | | **0.0004** | **1.42** | **0.0007** | |
| 51156 | | SERPINA10 | **3.52** | | | **0.0000** | | **7.77** | | **0.0000** | **4.87** | **0.0000** | |
| 12 | | SERPINA3 | **2.50** | | | **0.0004** | | **4.30** | | **0.0000** | **3.27** | **0.0000** | |
| 5269 | | SERPINB6 | **1.81** | | | **0.0000** | | **2.71** | | **0.0000** | **1.59** | **0.0000** | |
| 5272 | | SERPINB9 | **1.85** | | | **0.0000** | | **3.67** | | **0.0000** | **1.62** | **0.0000** | |
| 5270 | | SERPINE2 | **1.97** | | | **0.0000** | | **1.83** | | **0.0004** | **1.61** | **0.0000** | |
| 83442 | | SH3BGRL3 | **2.12** | | | **0.0000** | | **3.54** | | **0.0000** | **1.82** | **0.0000** | |
| 6472 | | SHMT2 | **0.66** | | | **0.0000** | | **0.56** | | **0.0005** | **0.65** | **0.0000** | |
| 23410 | | SIRT3 | **0.64** | | | **0.0000** | | **0.58** | | **0.0005** | **0.66** | **0.0000** | |
| 89886 | | SLAMF9 | **2.92** | | | **0.0000** | | **1.84** | | **0.0005** | **1.99** | **0.0000** | |
| 9058 | | SLC13A2 | **0.67** | | | **0.0006** | | **0.53** | | **0.0000** | **0.56** | **0.0000** | |
| 51296 | | SLC15A3 | **2.51** | | | **0.0000** | | **1.85** | | **0.0000** | **1.96** | **0.0000** | |
| 9122 | | SLC16A4 | **0.40** | | | **0.0000** | | **0.27** | | **0.0000** | **0.50** | **0.0000** | |
| 9194 | | SLC16A7 | **0.54** | | | **0.0000** | | **0.50** | | **0.0000** | **0.60** | **0.0000** | |
| 220963 | | SLC16A9 | **0.64** | | | **0.0006** | | **0.49** | | **0.0000** | **0.59** | **0.0000** | |
| 6570 | | SLC18A1 | **0.61** | | | **0.0000** | | **0.43** | | **0.0000** | **0.64** | **0.0005** | |
| 387775 | | SLC22A10 | **0.59** | | | **0.0004** | | **0.46** | | **0.0000** | **0.49** | **0.0000** | |
| 6583 | | SLC22A4 | **0.60** | | | **0.0000** | | **0.50** | | **0.0009** | **0.53** | **0.0000** | |
| 29957 | | SLC25A24 | **1.92** | | | **0.0000** | | **4.30** | | **0.0000** | **2.53** | **0.0000** | |
| 284439 | | SLC25A42 | **0.68** | | | **0.0007** | | **0.54** | | **0.0000** | **0.64** | **0.0000** | |
| 6518 | | SLC2A5 | **0.69** | | | **0.0008** | | **0.47** | | **0.0000** | **0.61** | **0.0000** | |
| 10568 | | SLC34A2 | **2.35** | | | **0.0000** | | **4.83** | | **0.0000** | **2.05** | **0.0000** | |
| 2542 | | SLC37A4 | **0.63** | | | **0.0000** | | **0.50** | | **0.0000** | **0.68** | **0.0000** | |
| 340024 | | SLC6A19 | **0.41** | | | **0.0000** | | **0.38** | | **0.0009** | **0.67** | **0.0000** | |
| 27293 | | SMPDL3B | **2.18** | | | **0.0000** | | **7.53** | | **0.0000** | **2.06** | **0.0000** | |
| 9021 | | SOCS3 | **5.20** | | | **0.0000** | | **7.62** | | **0.0000** | **2.74** | **0.0000** | |
| 170463 | | SSBP4 | **1.50** | | | **0.0000** | | **1.71** | | **0.0000** | **1.50** | **0.0000** | |
| 6774 | | STAT3 | **1.77** | | | **0.0000** | | **2.26** | | **0.0000** | **1.55** | **0.0000** | |
| 9262 | | STK17B | **2.37** | | | **0.0000** | | **1.67** | | **0.0004** | **1.68** | **0.0000** | |
| 8676 | | STX11 | **1.51** | | | **0.0000** | | **2.70** | | **0.0000** | **1.59** | **0.0000** | |
| 6821 | | SUOX | **0.66** | | | **0.0000** | | **0.54** | | **0.0005** | **0.61** | **0.0000** | |
| 56241 | | SUSD2 | **0.53** | | | **0.0000** | | **0.28** | | **0.0000** | **0.60** | **0.0000** | |
| 4070 | | TACSTD2 | **1.77** | | | **0.0000** | | **3.01** | | **0.0005** | **1.88** | **0.0000** | |
| 8407 | | TAGLN2 | **1.52** | | | **0.0000** | | **1.67** | | **0.0006** | **1.41** | **0.0000** | |
| 6891 | | TAP2 | **1.79** | | | **0.0000** | | **2.44** | | **0.0004** | **1.47** | **0.0000** | |
| 6892 | | TAPBP | **1.67** | | | **0.0000** | | **2.10** | | **0.0005** | **1.59** | **0.0000** | |
| 7040 | | TGFB1 | **1.96** | | | **0.0000** | | **1.64** | | **0.0004** | **1.56** | **0.0000** | |
| 7045 | | TGFBI | **3.91** | | | **0.0000** | | **2.87** | | **0.0000** | **2.94** | **0.0000** | |
| 55258 | | THNSL2 | **0.63** | | | **0.0000** | | **0.46** | | **0.0000** | **0.68** | **0.0000** | |
| 92610 | | TIFA | **1.76** | | | **0.0000** | | **2.59** | | **0.0000** | **1.70** | **0.0000** | |
| 7076 | | TIMP1 | **2.79** | | | **0.0000** | | **4.99** | | **0.0000** | **4.45** | **0.0000** | |
| 7097 | | TLR2 | **2.03** | | | **0.0000** | | **3.24** | | **0.0000** | **1.72** | **0.0000** | |
| 340061 | | TMEM173 | **2.15** | | | **0.0000** | | **3.30** | | **0.0000** | **1.72** | **0.0000** | |
| 144110 | | TMEM86A | **0.62** | | | **0.0000** | | **0.34** | | **0.0000** | **0.50** | **0.0000** | |
| 26022 | | TMEM98 | **1.53** | | | **0.0000** | | **1.60** | | **0.0009** | **1.53** | **0.0000** | |
| 7128 | | TNFAIP3 | **1.64** | | | **0.0000** | | **2.66** | | **0.0000** | **1.56** | **0.0000** | |
| 51330 | | TNFRSF12A | **2.32** | | | **0.0000** | | **5.47** | | **0.0000** | **1.87** | **0.0000** | |
| 203068 | | TUBB | **1.56** | | | **0.0000** | | **1.88** | | **0.0000** | **1.81** | **0.0000** | |
| 7305 | | TYROBP | **4.09** | | | **0.0000** | | **2.87** | | **0.0000** | **2.36** | **0.0000** | |
| 10537 | | UBD | **5.67** | | | **0.0000** | | **18.67** | | **0.0008** | **2.60** | **0.0000** | |
| 51733 | | UPB1 | **0.67** | | | **0.0008** | | **0.55** | | **0.0004** | **0.66** | **0.0000** | |
| 7412 | | VCAM1 | **2.75** | | | **0.0000** | | **10.71** | | **0.0000** | **2.72** | **0.0000** | |
| 7431 | | VIM | **2.19** | | | **0.0000** | | **2.92** | | **0.0000** | **2.56** | **0.0000** | |
| 220001 | | VWCE | **0.62** | | | **0.0000** | | **0.38** | | **0.0000** | **0.69** | **0.0000** | |
| **E. 240 genes regulated in NZB/W and NZM2410.** | | | | | | | | | | | | | |
| Human Entrez Gene ID | | Human Gene symbol | **NZB/W** | | | | | **NZM2410** | | | **NZW/BXSB** | | |
|  |  |  | Fold-change | | | q-value | | Fold-change | | q-value | Fold-change | q-value | |
| 97 | | ACYP1 | **0.70** | | | **0.0006** | | **0.68** | | **0.0005** | 0.79 | 0.0014 | |
| 183 | | AGT | **1.48** | | | **0.0004** | | **2.67** | | **0.0006** | 1.10 | 0.1837 | |
| 205 | | AK4 | **0.60** | | | **0.0006** | | **0.44** | | **0.0005** | 0.69 | 0.0056 | |
| 249 | | ALPL | **0.62** | | | **0.0000** | | **0.53** | | **0.0000** | 0.89 | 0.0310 | |
| 327 | | APEH | **0.64** | | | **0.0000** | | **0.58** | | **0.0004** | 0.75 | 0.0023 | |
| 384 | | ARG2 | **1.98** | | | **0.0004** | | **2.25** | | **0.0000** | 1.79 | 0.0710 | |
| 409 | | ARRB2 | **2.12** | | | **0.0000** | | **1.63** | | **0.0006** | 1.33 | 0.0005 | |
| 435 | | ASL | **0.56** | | | **0.0000** | | **0.52** | | **0.0000** | 0.93 | 0.1203 | |
| 467 | | ATF3 | **1.51** | | | **0.0006** | | **3.85** | | **0.0000** | 0.90 | 0.3591 | |
| 501 | | ALDH7A1 | **0.69** | | | **0.0000** | | **0.65** | | **0.0008** | 0.73 | 0.0000 | |
| 549 | | AUH | **0.70** | | | **0.0000** | | **0.59** | | **0.0000** | 0.73 | 0.0000 | |
| 594 | | BCKDHB | **0.55** | | | **0.0000** | | **0.51** | | **0.0005** | 0.75 | 0.0000 | |
| 762 | | CA4 | **0.57** | | | **0.0000** | | **0.60** | | **0.0004** | 0.79 | 0.0155 | |
| 771 | | CA12 | **0.62** | | | **0.0000** | | **0.60** | | **0.0000** | 1.02 | 0.9999 | |
| 785 | | CACNB4 | **0.69** | | | **0.0006** | | **0.60** | | **0.0004** | 0.73 | 0.0000 | |
| 865 | | CBFB | **1.47** | | | **0.0000** | | **1.51** | | **0.0005** | 1.05 | 0.3177 | |
| 866 | | SERPINA6 | **0.22** | | | **0.0000** | | **0.04** | | **0.0000** | np | | |
| 885 | | CCK | **0.68** | | | **0.0006** | | **0.41** | | **0.0000** | 0.74 | 0.0049 | |
| 886 | | CCKAR | **0.58** | | | **0.0000** | | **0.47** | | **0.0004** | 0.62 | 0.0043 | |
| 1137 | | CHRNA4 | **0.71** | | | **0.0000** | | **0.54** | | **0.0009** | 1.00 | 0.9999 | |
| 1316 | | KLF6 | **2.30** | | | **0.0000** | | **5.27** | | **0.0000** | 1.71 | 0.0019 | |
| 1355 | | COX15 | **0.63** | | | **0.0000** | | **0.71** | | **0.0007** | 0.84 | 0.0011 | |
| 1363 | | CPE | **2.55** | | | **0.0008** | | **5.80** | | **0.0006** | 1.77 | 0.0011 | |
| 1435 | | CSF1 | **1.49** | | | **0.0000** | | **2.05** | | **0.0000** | 1.34 | 0.0000 | |
| 1800 | | DPEP1 | **0.55** | | | **0.0000** | | **0.40** | | **0.0004** | 0.78 | 0.0027 | |
| 1892 | | ECHS1 | **0.64** | | | **0.0000** | | **0.56** | | **0.0005** | 0.80 | 0.0000 | |
| 2039 | | EPB49 | **0.65** | | | **0.0000** | | **0.57** | | **0.0000** | 0.93 | 0.1683 | |
| 2181 | | ACSL3 | **0.65** | | | **0.0000** | | **0.61** | | **0.0000** | 0.92 | 0.0944 | |
| 2203 | | FBP1 | **0.68** | | | **0.0000** | | **0.63** | | **0.0007** | 0.84 | 0.0019 | |
| 2230 | | FDX1 | **0.68** | | | **0.0000** | | **0.64** | | **0.0005** | 0.82 | 0.0047 | |
| 2243 | | FGA | **3.61** | | | **0.0000** | | **4.53** | | **0.0005** | 1.44 | 0.2188 | |
| 2244 | | FGB | **2.57** | | | **0.0004** | | **4.82** | | **0.0005** | 1.83 | 0.0273 | |
| 2326 | | FMO1 | **0.55** | | | **0.0000** | | **0.53** | | **0.0009** | 0.77 | 0.0003 | |
| 2329 | | FMO4 | **0.71** | | | **0.0004** | | **0.70** | | **0.0009** | 0.86 | 0.1065 | |
| 2593 | | GAMT | **0.70** | | | **0.0000** | | **0.54** | | **0.0000** | 0.77 | 0.0003 | |
| 2639 | | GCDH | **0.64** | | | **0.0000** | | **0.58** | | **0.0004** | 0.77 | 0.0000 | |
| 2896 | | GRN | **1.48** | | | **0.0000** | | **1.88** | | **0.0006** | 1.37 | 0.0003 | |
| 3029 | | HAGH | **0.70** | | | **0.0000** | | **0.70** | | **0.0004** | 0.80 | 0.0000 | |
| 3033 | | HADH | **0.69** | | | **0.0000** | | **0.71** | | **0.0004** | 0.82 | 0.0000 | |
| 3077 | | HFE | **1.42** | | | **0.0000** | | **1.59** | | **0.0000** | 1.31 | 0.0003 | |
| 3117 | | HLA-DQA1 | **2.82** | | | **0.0000** | | **4.53** | | **0.0000** | 1.53 | 0.0024 | |
| 3123 | | HLA-DRB1 | **2.49** | | | **0.0000** | | **4.27** | | **0.0000** | 1.51 | 0.0062 | |
| 3172 | | HNF4A | **0.69** | | | **0.0003** | | **0.59** | | **0.0005** | 0.90 | 0.0109 | |
| 3420 | | IDH3B | **0.65** | | | **0.0000** | | **0.64** | | **0.0000** | 0.81 | 0.0000 | |
| 3512 | | IGJ | **10.91** | | | **0.0000** | | **19.37** | | **0.0006** | 2.33 | 0.0019 | |
| 3710 | | ITPR3 | **1.45** | | | **0.0000** | | **1.84** | | **0.0009** | 1.30 | 0.0029 | |
| 3992 | | FADS1 | **0.66** | | | **0.0000** | | **0.59** | | **0.0000** | 1.03 | 0.3729 | |
| 3995 | | FADS3 | **0.68** | | | **0.0004** | | **0.66** | | **0.0005** | 0.98 | 0.9999 | |
| 4036 | | LRP2 | **0.56** | | | **0.0000** | | **0.52** | | **0.0004** | 0.87 | 0.0041 | |
| 4067 | | LYN | **1.75** | | | **0.0000** | | **1.44** | | **0.0009** | 1.39 | 0.0000 | |
| 4214 | | MAP3K1 | **1.41** | | | **0.0004** | | **2.18** | | **0.0004** | 1.38 | 0.0000 | |
| 4225 | | MEP1B | **0.54** | | | **0.0006** | | **0.31** | | **0.0000** | 0.56 | 0.0037 | |
| 4329 | | ALDH6A1 | **0.66** | | | **0.0006** | | **0.64** | | **0.0009** | 0.78 | 0.0000 | |
| 4482 | | MSRA | **0.68** | | | **0.0000** | | **0.64** | | **0.0004** | 0.90 | 0.0041 | |
| 4522 | | MTHFD1 | **0.66** | | | **0.0000** | | **0.61** | | **0.0000** | 0.82 | 0.0005 | |
| 4716 | | NDUFB10 | **0.69** | | | **0.0003** | | **0.65** | | **0.0000** | 0.89 | 0.0070 | |
| 4717 | | NDUFC1 | **0.70** | | | **0.0000** | | **0.70** | | **0.0007** | 0.89 | 0.0060 | |
| 4719 | | NDUFS1 | **0.67** | | | **0.0000** | | **0.59** | | **0.0005** | 0.81 | 0.0003 | |
| 4725 | | NDUFS5 | **0.71** | | | **0.0000** | | **0.71** | | **0.0000** | 0.87 | 0.0028 | |
| 5002 | | SLC22A18 | **0.56** | | | **0.0000** | | **0.53** | | **0.0006** | 0.77 | 0.0003 | |
| 5054 | | SERPINE1 | **2.93** | | | **0.0000** | | **5.83** | | **0.0005** | 1.72 | 0.0043 | |
| 5092 | | PCBD1 | **0.59** | | | **0.0000** | | **0.58** | | **0.0004** | 0.83 | 0.0087 | |
| 5095 | | PCCA | **0.66** | | | **0.0000** | | **0.55** | | **0.0000** | 0.76 | 0.0000 | |
| 5169 | | ENPP3 | **0.64** | | | **0.0000** | | **0.65** | | **0.0009** | 0.74 | 0.0009 | |
| 5174 | | PDZK1 | **0.62** | | | **0.0000** | | **0.57** | | **0.0000** | 0.88 | 0.0520 | |
| 5224 | | PGAM2 | **0.65** | | | **0.0004** | | **0.68** | | **0.0000** | 0.77 | 0.0000 | |
| 5447 | | POR | **0.66** | | | **0.0000** | | **0.54** | | **0.0000** | 0.84 | 0.0014 | |
| 5590 | | PRKCZ | **0.71** | | | **0.0000** | | **0.60** | | **0.0000** | 0.80 | 0.0106 | |
| 5608 | | MAP2K6 | **0.64** | | | **0.0000** | | **0.64** | | **0.0000** | 0.78 | 0.0046 | |
| 5627 | | PROS1 | **1.64** | | | **0.0000** | | **1.53** | | **0.0006** | 1.34 | 0.0005 | |
| 5634 | | PRPS2 | **0.65** | | | **0.0000** | | **0.70** | | **0.0009** | 0.78 | 0.0000 | |
| 5880 | | RAC2 | **1.80** | | | **0.0000** | | **1.59** | | **0.0007** | 1.34 | 0.0003 | |
| 5970 | | RELA | **1.46** | | | **0.0000** | | **1.85** | | **0.0000** | 1.27 | 0.0000 | |
| 6236 | | RRAD | **1.58** | | | **0.0000** | | **2.89** | | **0.0000** | 1.11 | 0.2499 | |
| 6281 | | S100A10 | **1.49** | | | **0.0000** | | **2.25** | | **0.0000** | 1.35 | 0.0003 | |
| 6389 | | SDHA | **0.71** | | | **0.0000** | | **0.69** | | **0.0005** | 0.81 | 0.0000 | |
| 6390 | | SDHB | **0.69** | | | **0.0000** | | **0.69** | | **0.0000** | 0.90 | 0.0000 | |
| 6470 | | SHMT1 | **0.70** | | | **0.0009** | | **0.68** | | **0.0008** | 0.97 | 0.2890 | |
| 6489 | | ST8SIA1 | **0.68** | | | **0.0000** | | **0.60** | | **0.0004** | 0.99 | 0.9999 | |
| 6517 | | SLC2A4 | **0.70** | | | **0.0000** | | **0.63** | | **0.0000** | 0.82 | 0.0104 | |
| 6520 | | SLC3A2 | **0.71** | | | **0.0006** | | **0.65** | | **0.0004** | 1.05 | 0.3191 | |
| 6524 | | SLC5A2 | **0.44** | | | **0.0000** | | **0.38** | | **0.0004** | 0.76 | 0.0035 | |
| 6546 | | SLC8A1 | **0.54** | | | **0.0000** | | **0.47** | | **0.0000** | 0.70 | 0.0032 | |
| 6555 | | SLC10A2 | **0.65** | | | **0.0000** | | **0.43** | | **0.0000** | 0.58 | 0.0035 | |
| 6569 | | SLC34A1 | **0.54** | | | **0.0000** | | **0.46** | | **0.0009** | 0.90 | 0.0399 | |
| 6648 | | SOD2 | **0.70** | | | **0.0000** | | **0.67** | | **0.0004** | 0.82 | 0.0000 | |
| 6652 | | SORD | **0.61** | | | **0.0000** | | **0.49** | | **0.0004** | 0.86 | 0.0000 | |
| 6694 | | SPP2 | **0.60** | | | **0.0004** | | **0.54** | | **0.0009** | 0.79 | 0.0129 | |
| 6909 | | TBX2 | **0.69** | | | **0.0006** | | **0.68** | | **0.0000** | 0.88 | 0.0399 | |
| 6920 | | TCEA3 | **0.61** | | | **0.0000** | | **0.55** | | **0.0000** | 0.74 | 0.0000 | |
| 7008 | | TEF | **0.69** | | | **0.0004** | | **0.56** | | **0.0000** | 0.68 | 0.0013 | |
| 7037 | | TFRC | **0.64** | | | **0.0000** | | **0.35** | | **0.0000** | 0.74 | 0.0520 | |
| 7078 | | TIMP3 | **0.69** | | | **0.0000** | | **0.61** | | **0.0005** | 0.83 | 0.0114 | |
| 7107 | | GPR137B | **0.61** | | | **0.0000** | | **0.56** | | **0.0004** | 0.81 | 0.0230 | |
| 7385 | | UQCRC2 | **0.69** | | | **0.0006** | | **0.71** | | **0.0000** | 0.87 | 0.0003 | |
| 7422 | | VEGFA | **0.71** | | | **0.0000** | | **0.61** | | **0.0004** | 0.71 | 0.0000 | |
| 7454 | | WAS | **2.07** | | | **0.0000** | | **1.48** | | **0.0009** | 1.33 | 0.0004 | |
| 7456 | | WIPF1 | **1.66** | | | **0.0000** | | **1.59** | | **0.0000** | 1.30 | 0.0000 | |
| 7511 | | XPNPEP1 | **0.70** | | | **0.0004** | | **0.65** | | **0.0009** | 1.09 | 0.0064 | |
| 8029 | | CUBN | **0.62** | | | **0.0000** | | **0.54** | | **0.0000** | 0.83 | 0.0005 | |
| 8209 | | C21orf33 | **0.68** | | | **0.0000** | | **0.63** | | **0.0000** | 0.82 | 0.0000 | |
| 8402 | | SLC25A11 | **0.69** | | | **0.0000** | | **0.67** | | **0.0004** | 0.84 | 0.0009 | |
| 8412 | | BCAR3 | **0.69** | | | **0.0000** | | **0.65** | | **0.0000** | 0.91 | 0.0406 | |
| 8528 | | DDO | **0.56** | | | **0.0000** | | **0.57** | | **0.0006** | 0.75 | 0.0520 | |
| 8572 | | PDLIM4 | **1.47** | | | **0.0000** | | **1.98** | | **0.0009** | 1.39 | 0.0003 | |
| 8659 | | ALDH4A1 | **0.60** | | | **0.0000** | | **0.53** | | **0.0000** | 0.72 | 0.0000 | |
| 8706 | | B3GALNT1 | **0.44** | | | **0.0000** | | **0.42** | | **0.0000** | 0.99 | 0.9999 | |
| 8803 | | SUCLA2 | **0.69** | | | **0.0004** | | **0.68** | | **0.0004** | 0.81 | 0.0000 | |
| 8858 | | PROZ | **0.68** | | | **0.0000** | | **0.49** | | **0.0005** | 0.80 | 0.0357 | |
| 8884 | | SLC5A6 | **0.56** | | | **0.0000** | | **0.46** | | **0.0004** | 0.67 | 0.0030 | |
| 9023 | | CH25H | **1.76** | | | **0.0000** | | **2.17** | | **0.0006** | 1.50 | 0.0095 | |
| 9054 | | NFS1 | **0.67** | | | **0.0000** | | **0.68** | | **0.0006** | 0.76 | 0.0000 | |
| 9086 | | EIF1AY | **1.42** | | | **0.0004** | | **1.80** | | **0.0004** | 1.25 | 0.0000 | |
| 9131 | | AIFM1 | **0.67** | | | **0.0000** | | **0.70** | | **0.0004** | 0.81 | 0.0000 | |
| 9197 | | SLC33A1 | **0.66** | | | **0.0007** | | **0.63** | | **0.0000** | 0.80 | 0.0484 | |
| 9365 | | KL | **0.46** | | | **0.0000** | | **0.31** | | **0.0000** | 0.69 | 0.0026 | |
| 9415 | | FADS2 | **0.58** | | | **0.0000** | | **0.52** | | **0.0004** | 0.82 | 0.0192 | |
| 9510 | | ADAMTS1 | **2.13** | | | **0.0009** | | **4.91** | | **0.0000** | 1.47 | 0.0084 | |
| 9512 | | PMPCB | **0.63** | | | **0.0000** | | **0.61** | | **0.0009** | 0.76 | 0.0003 | |
| 9843 | | HEPH | **0.71** | | | **0.0000** | | **0.55** | | **0.0000** | 0.95 | 0.2325 | |
| 9892 | | SNAP91 | **0.47** | | | **0.0000** | | **0.46** | | **0.0005** | 0.98 | 0.9999 | |
| 10110 | | SGK2 | **0.68** | | | **0.0007** | | **0.56** | | **0.0000** | 0.75 | 0.0000 | |
| 10128 | | LRPPRC | **0.68** | | | **0.0000** | | **0.66** | | **0.0000** | 0.84 | 0.0005 | |
| 10144 | | FAM13A | **0.62** | | | **0.0000** | | **0.59** | | **0.0000** | 0.71 | 0.0049 | |
| 10243 | | GPHN | **0.63** | | | **0.0000** | | **0.70** | | **0.0005** | 0.78 | 0.0029 | |
| 10312 | | TCIRG1 | **1.53** | | | **0.0000** | | **1.43** | | **0.0006** | 1.35 | 0.0000 | |
| 10542 | | HBXIP | **0.66** | | | **0.0000** | | **0.70** | | **0.0006** | 0.86 | 0.0056 | |
| 10659 | | CELF2 | **1.41** | | | **0.0000** | | **1.50** | | **0.0000** | 1.25 | 0.0000 | |
| 10669 | | CGREF1 | **0.68** | | | **0.0008** | | **0.51** | | **0.0006** | 0.75 | 0.0000 | |
| 10673 | | TNFSF13B | **2.06** | | | **0.0000** | | **1.56** | | **0.0006** | 1.36 | 0.0000 | |
| 10682 | | EBP | **0.70** | | | **0.0000** | | **0.58** | | **0.0000** | 0.73 | 0.0000 | |
| 11035 | | RIPK3 | **1.50** | | | **0.0000** | | **1.69** | | **0.0005** | 1.25 | 0.0005 | |
| 11136 | | SLC7A9 | **0.66** | | | **0.0000** | | **0.65** | | **0.0008** | 0.99 | 0.9999 | |
| 11274 | | USP18 | **2.34** | | | **0.0000** | | **2.17** | | **0.0000** | 2.36 | 0.0046 | |
| 22849 | | CPEB3 | **0.65** | | | **0.0000** | | **0.35** | | **0.0000** | 0.64 | 0.0011 | |
| 22921 | | MSRB2 | **0.69** | | | **0.0000** | | **0.67** | | **0.0006** | 0.89 | 0.0310 | |
| 23315 | | SLC9A8 | **0.60** | | | **0.0003** | | **0.54** | | **0.0008** | 0.66 | 0.0289 | |
| 23428 | | SLC7A8 | **0.46** | | | **0.0000** | | **0.38** | | **0.0000** | 0.87 | 0.0377 | |
| 23464 | | GCAT | **0.70** | | | **0.0000** | | **0.57** | | **0.0000** | 1.00 | 0.9999 | |
| 23566 | | LPAR3 | **0.35** | | | **0.0000** | | **0.44** | | **0.0005** | 0.88 | 0.0784 | |
| 23600 | | AMACR | **0.59** | | | **0.0000** | | **0.56** | | **0.0004** | 0.73 | 0.0533 | |
| 23705 | | CADM1 | **1.67** | | | **0.0000** | | **2.10** | | **0.0009** | 1.13 | 0.0165 | |
| 23764 | | MAFF | **1.54** | | | **0.0007** | | **2.09** | | **0.0005** | 1.05 | 0.3255 | |
| 25864 | | ABHD14A | **0.56** | | | **0.0000** | | **0.49** | | **0.0000** | 1.14 | 0.2413 | |
| 25974 | | MMACHC | **0.62** | | | **0.0000** | | **0.68** | | **0.0009** | 0.77 | 0.0000 | |
| 25975 | | EGFL6 | **0.50** | | | **0.0004** | | **0.36** | | **0.0000** | 0.64 | 0.0035 | |
| 26353 | | HSPB8 | **1.47** | | | **0.0006** | | **2.15** | | **0.0005** | 1.42 | 0.0011 | |
| 27253 | | PCDH17 | **0.59** | | | **0.0000** | | **0.53** | | **0.0007** | 0.86 | 0.0533 | |
| 28512 | | NKIRAS1 | **0.70** | | | **0.0004** | | **0.64** | | **0.0007** | 0.86 | 0.0036 | |
| 29760 | | BLNK | **1.43** | | | **0.0000** | | **1.96** | | **0.0006** | 1.25 | 0.0007 | |
| 50507 | | NOX4 | **0.42** | | | **0.0000** | | **0.41** | | **0.0005** | 0.88 | 0.0109 | |
| 50808 | | AK3 | **0.60** | | | **0.0000** | | **0.68** | | **0.0006** | 0.82 | 0.0000 | |
| 50814 | | NSDHL | **0.59** | | | **0.0000** | | **0.59** | | **0.0000** | 0.93 | 0.1512 | |
| 51008 | | ASCC1 | **0.68** | | | **0.0000** | | **0.59** | | **0.0000** | 0.93 | 0.2090 | |
| 51084 | | CRYL1 | **0.68** | | | **0.0000** | | **0.57** | | **0.0000** | 0.84 | 0.0004 | |
| 51181 | | DCXR | **0.64** | | | **0.0000** | | **0.41** | | **0.0000** | 0.73 | 0.0000 | |
| 51278 | | IER5 | **1.43** | | | **0.0000** | | **1.66** | | **0.0000** | 1.24 | 0.0135 | |
| 51537 | | MTFP1 | **0.62** | | | **0.0000** | | **0.57** | | **0.0006** | 0.81 | 0.0021 | |
| 51762 | | RAB8B | **1.61** | | | **0.0000** | | **1.54** | | **0.0004** | 1.27 | 0.0022 | |
| 54808 | | DYM | **0.59** | | | **0.0000** | | **0.49** | | **0.0000** | 0.87 | 0.0007 | |
| 54927 | | CHCHD3 | **0.67** | | | **0.0000** | | **0.65** | | **0.0000** | 0.88 | 0.0019 | |
| 54943 | | DNAJC28 | **0.68** | | | **0.0000** | | **0.61** | | **0.0006** | 0.73 | 0.0000 | |
| 54968 | | TMEM70 | **0.65** | | | **0.0000** | | **0.67** | | **0.0009** | 0.81 | 0.0000 | |
| 55245 | | UQCC | **0.68** | | | **0.0000** | | **0.64** | | **0.0000** | 0.80 | 0.0003 | |
| 55248 | | TMEM206 | **0.71** | | | **0.0000** | | **0.51** | | **0.0000** | 0.94 | 0.1512 | |
| 55337 | | C19orf66 | **1.47** | | | **0.0000** | | **1.66** | | **0.0004** | 1.27 | 0.0000 | |
| 55437 | | STRADB | **0.68** | | | **0.0000** | | **0.66** | | **0.0000** | 0.75 | 0.0011 | |
| 55576 | | STAB2 | **0.61** | | | **0.0000** | | **0.26** | | **0.0000** | 0.89 | 0.0474 | |
| 55620 | | STAP2 | **1.49** | | | **0.0000** | | **2.03** | | **0.0005** | 1.38 | 0.0000 | |
| 55825 | | PECR | **0.61** | | | **0.0000** | | **0.48** | | **0.0000** | 0.83 | 0.0668 | |
| 55843 | | ARHGAP15 | **1.41** | | | **0.0000** | | **1.48** | | **0.0000** | 1.28 | 0.0000 | |
| 55862 | | ECHDC1 | **0.63** | | | **0.0000** | | **0.67** | | **0.0009** | 0.88 | 0.0179 | |
| 55916 | | NXT2 | **0.63** | | | **0.0006** | | **0.69** | | **0.0005** | 0.83 | 0.0055 | |
| 56034 | | PDGFC | **0.60** | | | **0.0000** | | **0.65** | | **0.0000** | 1.02 | 0.3763 | |
| 56521 | | DNAJC12 | **0.49** | | | **0.0000** | | **0.37** | | **0.0000** | 0.65 | 0.0011 | |
| 56654 | | NPDC1 | **1.44** | | | **0.0000** | | **1.62** | | **0.0007** | 1.25 | 0.0035 | |
| 56898 | | BDH2 | **0.59** | | | **0.0000** | | **0.57** | | **0.0004** | 0.81 | 0.0011 | |
| 56954 | | NIT2 | **0.57** | | | **0.0000** | | **0.58** | | **0.0004** | 0.83 | 0.0043 | |
| 57017 | | COQ9 | **0.67** | | | **0.0000** | | **0.60** | | **0.0003** | 0.84 | 0.0047 | |
| 57698 | | KIAA1598 | **1.49** | | | **0.0000** | | **2.36** | | **0.0006** | 1.21 | 0.0047 | |
| 60488 | | MRPS35 | **0.71** | | | **0.0000** | | **0.67** | | **0.0000** | 0.85 | 0.0029 | |
| 63027 | | SLC22A23 | **0.62** | | | **0.0000** | | **0.60** | | **0.0000** | 1.06 | 0.1870 | |
| 63917 | | GALNT11 | **0.60** | | | **0.0000** | | **0.50** | | **0.0000** | 0.83 | 0.0325 | |
| 64087 | | MCCC2 | **0.64** | | | **0.0000** | | **0.57** | | **0.0006** | 0.73 | 0.0000 | |
| 64172 | | OSGEPL1 | **0.70** | | | **0.0006** | | **0.64** | | **0.0003** | 0.82 | 0.0000 | |
| 64857 | | PLEKHG2 | **1.41** | | | **0.0000** | | **1.44** | | **0.0000** | 1.32 | 0.0000 | |
| 64922 | | LRRC19 | **0.63** | | | **0.0004** | | **0.62** | | **0.0009** | 0.78 | 0.0000 | |
| 79944 | | L2HGDH | **0.60** | | | **0.0000** | | **0.52** | | **0.0006** | 0.80 | 0.0000 | |
| 81570 | | CLPB | **0.61** | | | **0.0000** | | **0.69** | | **0.0007** | 0.75 | 0.0000 | |
| 81693 | | AMN | **0.69** | | | **0.0006** | | **0.68** | | **0.0004** | 1.03 | 0.3549 | |
| 81889 | | FAHD1 | **0.58** | | | **0.0000** | | **0.58** | | **0.0006** | 0.82 | 0.0011 | |
| 83593 | | RASSF5 | **1.63** | | | **0.0000** | | **1.48** | | **0.0004** | 1.20 | 0.0254 | |
| 83660 | | TLN2 | **0.67** | | | **0.0000** | | **0.54** | | **0.0000** | 0.82 | 0.0037 | |
| 83853 | | ROPN1L | **0.68** | | | **0.0000** | | **0.68** | | **0.0006** | 0.79 | 0.0005 | |
| 84326 | | C16orf13 | **0.71** | | | **0.0004** | | **0.69** | | **0.0005** | 0.80 | 0.0014 | |
| 84340 | | GFM2 | **0.67** | | | **0.0000** | | **0.62** | | **0.0005** | 0.83 | 0.0151 | |
| 84693 | | MCEE | **0.69** | | | **0.0003** | | **0.53** | | **0.0000** | 0.78 | 0.0000 | |
| 84866 | | TMEM25 | **0.68** | | | **0.0000** | | **0.65** | | **0.0000** | 0.85 | 0.0310 | |
| 85441 | | PRIC285 | **1.44** | | | **0.0000** | | **1.70** | | **0.0004** | 1.34 | 0.0003 | |
| 90507 | | SCRN2 | **0.70** | | | **0.0003** | | **0.66** | | **0.0008** | 0.82 | 0.0026 | |
| 90835 | | C16orf93 | **0.69** | | | **0.0000** | | **0.62** | | **0.0000** | 0.83 | 0.0033 | |
| 91614 | | DEPDC7 | **0.63** | | | **0.0000** | | **0.67** | | **0.0007** | 0.92 | 0.0651 | |
| 112849 | | C14orf149 | **0.62** | | | **0.0000** | | **0.56** | | **0.0007** | 0.95 | 0.2064 | |
| 114880 | | OSBPL6 | **0.68** | | | **0.0000** | | **0.68** | | **0.0006** | 0.73 | 0.0000 | |
| 118427 | | OLFM3 | **0.53** | | | **0.0000** | | **0.57** | | **0.0006** | 1.05 | 0.3320 | |
| 120892 | | LRRK2 | **0.67** | | | **0.0000** | | **0.62** | | **0.0000** | 0.86 | 0.1230 | |
| 123876 | | ACSM2A | **0.57** | | | **0.0003** | | **0.51** | | **0.0005** | 0.84 | 0.0059 | |
| 126308 | | MOB3A | **1.63** | | | **0.0000** | | **1.84** | | **0.0000** | 1.24 | 0.0005 | |
| 127018 | | LYPLAL1 | **0.68** | | | **0.0000** | | **0.67** | | **0.0005** | 0.87 | 0.0984 | |
| 128989 | | C22orf25 | **0.71** | | | **0.0000** | | **0.56** | | **0.0004** | 0.85 | 0.0710 | |
| 130589 | | GALM | **0.64** | | | **0.0000** | | **0.58** | | **0.0000** | 0.83 | 0.0005 | |
| 130814 | | PQLC3 | **1.73** | | | **0.0000** | | **1.50** | | **0.0000** | 1.40 | 0.0000 | |
| 133522 | | PPARGC1B | **0.66** | | | **0.0000** | | **0.62** | | **0.0004** | 0.80 | 0.0302 | |
| 134429 | | STARD4 | **0.61** | | | **0.0000** | | **0.70** | | **0.0008** | 1.03 | 0.3268 | |
| 142680 | | SLC34A3 | **0.42** | | | **0.0000** | | **0.20** | | **0.0000** | 0.53 | 0.0013 | |
| 143941 | | TTC36 | **0.68** | | | **0.0007** | | **0.60** | | **0.0000** | 0.81 | 0.0019 | |
| 148808 | | MFSD4 | **0.66** | | | **0.0000** | | **0.59** | | **0.0000** | 0.70 | 0.0013 | |
| 148811 | | PM20D1 | **0.63** | | | **0.0000** | | **0.59** | | **0.0004** | 0.74 | 0.0000 | |
| 149466 | | C1orf210 | **0.60** | | | **0.0000** | | **0.63** | | **0.0004** | 0.74 | 0.0000 | |
| 155066 | | ATP6V0E2 | **0.66** | | | **0.0000** | | **0.66** | | **0.0000** | 0.77 | 0.0008 | |
| 170482 | | CLEC4C | **3.52** | | | **0.0000** | | **1.68** | | **0.0000** | 1.78 | 0.0024 | |
| 196410 | | METTL7B | **0.62** | | | **0.0000** | | **0.49** | | **0.0000** | 0.87 | 0.0881 | |
| 200159 | | C1orf100 | **1.67** | | | **0.0000** | | **3.29** | | **0.0000** | 1.26 | 0.0024 | |
| 220107 | | DLEU7 | **0.47** | | | **0.0000** | | **0.38** | | **0.0000** | 1.19 | 0.2735 | |
| 283238 | | SLC22A24 | **0.39** | | | **0.0000** | | **0.24** | | **0.0003** | 0.73 | 0.0066 | |
| 283375 | | SLC39A5 | **0.70** | | | **0.0004** | | **0.55** | | **0.0000** | 0.72 | 0.0000 | |
| 284541 | | CYP4A22 | **2.45** | | | **0.0000** | | **6.75** | | **0.0004** | 0.65 | 0.0032 | |
| 284612 | | SYPL2 | **0.69** | | | **0.0000** | | **0.46** | | **0.0003** | 0.84 | 0.0594 | |
| 285440 | | CYP4V2 | **1.66** | | | **0.0000** | | **2.67** | | **0.0005** | 1.34 | 0.0000 | |
| 339123 | | JMJD8 | **0.68** | | | **0.0000** | | **0.70** | | **0.0005** | 0.94 | 0.1601 | |
| 344838 | | PAQR9 | **0.61** | | | **0.0004** | | **0.43** | | **0.0000** | 0.74 | 0.0007 | |
| 373156 | | GSTK1 | **0.67** | | | **0.0006** | | **0.55** | | **0.0000** | 0.79 | 0.0060 | |
| 375287 | | RBM43 | **1.45** | | | **0.0000** | | **1.47** | | **0.0005** | 1.38 | 0.0000 | |
| 387700 | | SLC16A12 | **0.61** | | | **0.0000** | | **0.68** | | **0.0005** | 0.93 | 0.1808 | |
| 389434 | | IYD | **0.60** | | | **0.0000** | | **0.60** | | **0.0005** | 0.74 | 0.0000 | |
| 392465 | | GLOD5 | **0.71** | | | **0.0006** | | **0.60** | | **0.0005** | 0.80 | 0.0017 | |
| 439996 | | IFIT1B | **3.07** | | | **0.0000** | | **3.60** | | **0.0006** | 3.20 | 0.0013 | |
| 641371 | | ACOT1 | **0.65** | | | **0.0007** | | **0.53** | | **0.0007** | 0.67 | 0.1626 | |
| 729967 | | MORN2 | **0.61** | | | **0.0004** | | **0.50** | | **0.0000** | 0.81 | 0.0377 | |
| 8970/85236 | | HIST1H2BJ/HIST1H2BK | **0.69** | | | **0.0006** | | **0.55** | | **0.0004** | 0.88 | 0.0241 | |
| **F. 126 genes regulated in the same direction in NZM2410 and NZW/BXSB.** | | | | | | | | | | | | |  |
| Human Entrez Gene ID | Human Gene symbol | | **NZB/W** | | | | | **NZM2410** | | | **NZW/BXSB** | |  |
|  |  |  | Fold-change | | | q-value | | Fold-change | | q-value | Fold-change | q-value |  |
| 19 | ABCA1 | | 1.85 | | | 0.0013 | | **2.31** | | **0.0000** | **2.01** | **0.0000** |  |
| 34 | ACADM | | 0.75 | | | 0.0018 | | **0.66** | | **0.0000** | **0.71** | **0.0000** |  |
| 95 | ACY1 | | 0.62 | | | 0.0011 | | **0.34** | | **0.0000** | **0.59** | **0.0000** |  |
| 230 | ALDOC | | 0.87 | | | 0.0976 | | **0.62** | | **0.0007** | **0.68** | **0.0007** |  |
| 290 | ANPEP | | 0.72 | | | 0.0048 | | **0.37** | | **0.0000** | **0.68** | **0.0000** |  |
| 308 | ANXA5 | | 1.30 | | | 0.0006 | | **1.81** | | **0.0000** | **1.55** | **0.0000** |  |
| 364 | AQP7 | | 1.09 | | | 0.3176 | | **0.62** | | **0.0004** | **0.62** | **0.0000** |  |
| 389 | RHOC | | 1.47 | | | 0.0016 | | **2.03** | | **0.0000** | **1.44** | **0.0000** |  |
| 622 | BDH1 | | 0.66 | | | 0.0018 | | **0.49** | | **0.0004** | **0.69** | **0.0007** |  |
| 699 | BUB1 | | 1.26 | | | 0.0368 | | **1.72** | | **0.0000** | **2.67** | **0.0000** |  |
| 795 | S100G | | 0.66 | | | 0.0031 | | **0.48** | | **0.0000** | **0.44** | **0.0000** |  |
| 799 | CALCR | | 1.13 | | | 0.2401 | | **3.24** | | **0.0006** | **1.63** | **0.0003** |  |
| 874 | CBR3 | | 1.56 | | | 0.0160 | | **2.19** | | **0.0000** | **1.51** | **0.0003** |  |
| 890 | CCNA2 | | 1.43 | | | 0.0080 | | **1.52** | | **0.0000** | **2.38** | **0.0000** |  |
| 990 | CDC6 | | np | | | | | **1.53** | | **0.0000** | **2.19** | **0.0002** |  |
| 1080 | CFTR | | np | | | | | **2.35** | | **0.0000** | **1.56** | **0.0003** |  |
| 1191 | CLU | | 1.62 | | | 0.0048 | | **3.04** | | **0.0000** | **1.96** | **0.0000** |  |
| 1230 | CCR1 | | 1.32 | | | 0.0216 | | **1.57** | | **0.0004** | **1.53** | **0.0000** |  |
| 1366 | CLDN7 | | 1.56 | | | 0.0039 | | **2.71** | | **0.0004** | **1.55** | **0.0000** |  |
| 1476 | CSTB | | 1.28 | | | 0.0053 | | **2.30** | | **0.0000** | **1.63** | **0.0000** |  |
| 1595 | CYP51A1 | | 0.62 | | | 0.0039 | | **0.38** | | **0.0000** | **0.51** | **0.0000** |  |
| 1809 | DPYSL3 | | 1.69 | | | 0.0041 | | **3.75** | | **0.0006** | **2.08** | **0.0000** |  |
| 1906 | EDN1 | | 1.85 | | | 0.0018 | | **2.94** | | **0.0000** | **1.94** | **0.0000** |  |
| 2267 | FGL1 | | 1.93 | | | 0.0013 | | **2.59** | | **0.0006** | **1.77** | **0.0003** |  |
| 2444 | FRK | | 1.28 | | | 0.0623 | | **2.26** | | **0.0007** | **1.49** | **0.0000** |  |
| 2817 | GPC1 | | 0.90 | | | 0.0918 | | **0.57** | | **0.0000** | **0.67** | **0.0000** |  |
| 2823 | GPM6A | | 0.77 | | | 0.0138 | | **0.64** | | **0.0003** | **0.67** | **0.0000** |  |
| 2920 | CXCL2 | | 1.84 | | | 0.0341 | | **5.47** | | **0.0000** | **2.35** | **0.0000** |  |
| 2921 | CXCL3 | | 1.67 | | | 0.0250 | | **4.77** | | **0.0000** | **2.93** | **0.0000** |  |
| 3426 | CFI | | 1.48 | | | 0.0014 | | **3.74** | | **0.0000** | **3.46** | **0.0000** |  |
| 3712 | IVD | | 0.72 | | | 0.0007 | | **0.62** | | **0.0004** | **0.71** | **0.0000** |  |
| 3898 | LAD1 | | 1.27 | | | 0.0264 | | **1.68** | | **0.0000** | **1.45** | **0.0003** |  |
| 3918 | LAMC2 | | 1.23 | | | 0.0013 | | **1.71** | | **0.0007** | **1.65** | **0.0000** |  |
| 4023 | LPL | | 0.67 | | | 0.0015 | | **0.34** | | **0.0000** | **0.58** | **0.0000** |  |
| 4059 | BCAM | | 1.55 | | | 0.0038 | | **1.83** | | **0.0004** | **1.51** | **0.0003** |  |
| 4173 | MCM4 | | 1.37 | | | 0.0035 | | **1.41** | | **0.0009** | **1.78** | **0.0000** |  |
| 5069 | PAPPA | | 1.43 | | | 0.0023 | | **1.98** | | **0.0004** | **1.70** | **0.0000** |  |
| 5091 | PC | | 0.71 | | | 0.0048 | | **0.48** | | **0.0000** | **0.61** | **0.0000** |  |
| 5196 | PF4 | | 1.82 | | | 0.0080 | | **2.88** | | **0.0000** | **2.13** | **0.0000** |  |
| 5264 | PHYH | | 0.75 | | | 0.0006 | | **0.70** | | **0.0009** | **0.70** | **0.0000** |  |
| 5618 | PRLR | | 0.53 | | | 0.0013 | | **0.31** | | **0.0006** | **2.56** | **0.0000** |  |
| 5827 | PXMP2 | | 0.69 | | | 0.0041 | | **0.54** | | **0.0004** | **0.56** | **0.0000** |  |
| 5836 | PYGL | | 0.76 | | | 0.0013 | | **0.56** | | **0.0004** | **0.62** | **0.0000** |  |
| 5888 | RAD51 | | 1.33 | | | 0.0044 | | **1.79** | | **0.0009** | **2.25** | **0.0000** |  |
| 6376 | CX3CL1 | | 1.48 | | | 0.0145 | | **1.77** | | **0.0000** | **1.47** | **0.0000** |  |
| 6478 | SIAH2 | | 0.76 | | | 0.0000 | | **0.65** | | **0.0000** | **0.70** | **0.0000** |  |
| 6696 | SPP1 | | 1.18 | | | 0.0505 | | **1.57** | | **0.0000** | **1.89** | **0.0000** |  |
| 6876 | TAGLN | | 1.47 | | | 0.0053 | | **2.70** | | **0.0008** | **2.00** | **0.0000** |  |
| 7132 | TNFRSF1A | | 1.38 | | | 0.0004 | | **1.42** | | **0.0000** | **1.44** | **0.0000** |  |
| 7153 | TOP2A | | 1.57 | | | 0.0017 | | **1.42** | | **0.0000** | **2.87** | **0.0000** |  |
| 8309 | ACOX2 | | 0.69 | | | 0.0021 | | **0.36** | | **0.0000** | **0.58** | **0.0000** |  |
| 8310 | ACOX3 | | 0.89 | | | 0.1437 | | **0.64** | | **0.0005** | **0.48** | **0.0000** |  |
| 8638 | OASL | | 1.54 | | | 0.0023 | | **3.02** | | **0.0000** | **2.17** | **0.0000** |  |
| 8842 | PROM1 | | 1.01 | | | 0.9999 | | **1.42** | | **0.0000** | **1.74** | **0.0000** |  |
| 8848 | TSC22D1 | | 1.50 | | | 0.0041 | | **2.71** | | **0.0000** | **1.50** | **0.0002** |  |
| 9055 | PRC1 | | 1.34 | | | 0.0048 | | **1.45** | | **0.0007** | **1.88** | **0.0000** |  |
| 9076 | CLDN1 | | 1.45 | | | 0.1531 | | **2.89** | | **0.0000** | **1.79** | **0.0000** |  |
| 9390 | SLC22A13 | | 0.75 | | | 0.0129 | | **0.57** | | **0.0000** | **0.69** | **0.0000** |  |
| 9507 | ADAMTS4 | | 1.36 | | | 0.0128 | | **1.59** | | **0.0005** | **1.50** | **0.0000** |  |
| 9517 | SPTLC2 | | 1.48 | | | 0.0048 | | **2.02** | | **0.0004** | **1.69** | **0.0000** |  |
| 9518 | GDF15 | | 2.22 | | | 0.0038 | | **4.84** | | **0.0000** | **2.24** | **0.0000** |  |
| 9824 | ARHGAP11A | | 1.26 | | | 0.0019 | | **1.44** | | **0.0000** | **1.87** | **0.0000** |  |
| 9963 | SLC23A1 | | 0.73 | | | 0.0007 | | **0.57** | | **0.0005** | **0.65** | **0.0000** |  |
| 10406 | WFDC2 | | 1.46 | | | 0.0240 | | **2.01** | | **0.0006** | **2.23** | **0.0000** |  |
| 10460 | TACC3 | | 1.25 | | | 0.0068 | | **1.60** | | **0.0004** | **1.56** | **0.0000** |  |
| 10468 | FST | | 1.74 | | | 0.0021 | | **3.05** | | **0.0004** | **1.46** | **0.0002** |  |
| 10497 | UNC13B | | 1.21 | | | 0.0207 | | **2.14** | | **0.0007** | **1.42** | **0.0003** |  |
| 10686 | CLDN16 | | 1.10 | | | 0.3806 | | **1.78** | | **0.0004** | **1.88** | **0.0000** |  |
| 10733 | PLK4 | | 1.18 | | | 0.0113 | | **1.59** | | **0.0004** | **1.62** | **0.0000** |  |
| 10800 | CYSLTR1 | | np | | | | | **1.49** | | **0.0009** | **1.48** | **0.0000** |  |
| 10864 | SLC22A7 | | 0.60 | | | 0.0013 | | **0.43** | | **0.0006** | **0.34** | **0.0000** |  |
| 11170 | FAM107A | | 0.66 | | | 0.0019 | | **0.56** | | **0.0000** | **0.65** | **0.0000** |  |
| 11343 | MGLL | | 0.78 | | | 0.0145 | | **0.62** | | **0.0000** | **0.66** | **0.0003** |  |
| 22881 | ANKRD6 | | 1.27 | | | 0.0075 | | **2.42** | | **0.0000** | **1.84** | **0.0000** |  |
| 23175 | LPIN1 | | 0.73 | | | 0.0194 | | **0.64** | | **0.0000** | **0.58** | **0.0000** |  |
| 23498 | HAAO | | 0.68 | | | 0.0013 | | **0.40** | | **0.0004** | **0.64** | **0.0000** |  |
| 23597 | ACOT9 | | 1.40 | | | 0.0118 | | **1.98** | | **0.0006** | **1.47** | **0.0000** |  |
| 23743 | BHMT2 | | 0.64 | | | 0.0011 | | **0.53** | | **0.0008** | **0.66** | **0.0000** |  |
| 25902 | MTHFD1L | | 1.39 | | | 0.0004 | | **1.55** | | **0.0000** | **1.51** | **0.0000** |  |
| 26136 | TES | | 1.37 | | | 0.0004 | | **1.89** | | **0.0000** | **1.49** | **0.0000** |  |
| 26275 | HIBCH | | 0.79 | | | 0.0282 | | **0.64** | | **0.0006** | **0.64** | **0.0000** |  |
| 26509 | MYOF | | 1.65 | | | 0.0023 | | **2.33** | | **0.0000** | **2.15** | **0.0000** |  |
| 27010 | TPK1 | | 0.78 | | | 0.0007 | | **0.69** | | **0.0000** | **0.70** | **0.0003** |  |
| 27330 | RPS6KA6 | | 1.18 | | | 0.0900 | | **1.88** | | **0.0000** | **1.60** | **0.0000** |  |
| 28992 | MACROD1 | | 0.79 | | | 0.0306 | | **0.57** | | **0.0008** | **0.55** | **0.0000** |  |
| 29128 | UHRF1 | | 1.48 | | | 0.0027 | | **1.91** | | **0.0005** | **2.40** | **0.0000** |  |
| 30008 | EFEMP2 | | 1.38 | | | 0.0000 | | **1.64** | | **0.0000** | **1.62** | **0.0000** |  |
| 51171 | HSD17B14 | | 0.94 | | | 0.2221 | | **0.67** | | **0.0008** | **0.70** | **0.0000** |  |
| 51659 | GINS2 | | 1.22 | | | 0.0290 | | **1.60** | | **0.0000** | **1.57** | **0.0000** |  |
| 54443 | ANLN | | 1.52 | | | 0.0023 | | **2.19** | | **0.0000** | **3.14** | **0.0000** |  |
| 54474 | KRT20 | | 2.37 | | | 0.0030 | | **17.06** | | **0.0000** | **2.18** | **0.0000** |  |
| 55013 | CCDC109B | | 1.37 | | | 0.0006 | | **1.99** | | **0.0006** | **1.62** | **0.0000** |  |
| 55055 | ZWILCH | | 1.18 | | | 0.0061 | | **1.57** | | **0.0007** | **1.96** | **0.0000** |  |
| 55083 | KIF26B | | 1.24 | | | 0.1132 | | **2.57** | | **0.0000** | **1.61** | **0.0000** |  |
| 56256 | SERTAD4 | | 1.37 | | | 0.0038 | | **1.94** | | **0.0000** | **1.65** | **0.0000** |  |
| 57142 | RTN4 | | 1.19 | | | 0.0727 | | **1.91** | | **0.0000** | **1.47** | **0.0000** |  |
| 57582 | KCNT1 | | 0.87 | | | 0.0216 | | **0.66** | | **0.0000** | **0.60** | **0.0000** |  |
| 64222 | TOR3A | | 1.39 | | | 0.0000 | | **2.04** | | **0.0000** | **1.57** | **0.0000** |  |
| 79634 | SCRN3 | | 0.74 | | | 0.0007 | | **0.68** | | **0.0004** | **0.70** | **0.0008** |  |
| 80114 | BICC1 | | 1.14 | | | 0.2163 | | **1.58** | | **0.0009** | **1.49** | **0.0000** |  |
| 80724 | ACAD10 | | 0.76 | | | 0.0102 | | **0.56** | | **0.0000** | **0.67** | **0.0003** |  |
| 80781 | COL18A1 | | 1.41 | | | 0.0133 | | **2.34** | | **0.0000** | **1.79** | **0.0000** |  |
| 83461 | CDCA3 | | 1.17 | | | 0.1531 | | **1.51** | | **0.0008** | **1.88** | **0.0000** |  |
| 84617 | TUBB6 | | 1.48 | | | 0.0021 | | **1.71** | | **0.0008** | **1.76** | **0.0000** |  |
| 85301 | COL27A1 | | 0.80 | | | 0.0207 | | **0.56** | | **0.0008** | **0.54** | **0.0000** |  |
| 91775 | FAM55C | | 0.76 | | | 0.0133 | | **0.45** | | **0.0000** | **0.52** | **0.0004** |  |
| 114571 | SLC22A9 | | 0.87 | | | 0.1064 | | **0.33** | | **0.0000** | **0.39** | **0.0000** |  |
| 115361 | GBP4 | | 1.49 | | | 0.0521 | | **2.79** | | **0.0007** | **2.51** | **0.0000** |  |
| 125488 | TTC39C | | 0.92 | | | 0.1798 | | **0.65** | | **0.0009** | **0.55** | **0.0000** |  |
| 128239 | IQGAP3 | | 1.08 | | | 0.2797 | | **1.57** | | **0.0008** | **1.77** | **0.0000** |  |
| 134265 | AFAP1L1 | | 0.76 | | | 0.0013 | | **0.70** | | **0.0009** | **0.60** | **0.0000** |  |
| 138428 | PTRH1 | | 1.13 | | | 0.1906 | | **1.83** | | **0.0000** | **1.56** | **0.0000** |  |
| 140803 | TRPM6 | | 0.72 | | | 0.0000 | | **0.53** | | **0.0005** | **0.62** | **0.0000** |  |
| 145567 | TTC7B | | 0.75 | | | 0.0000 | | **0.65** | | **0.0006** | **0.71** | **0.0000** |  |
| 160428 | ALDH1L2 | | 1.06 | | | 0.3334 | | **1.49** | | **0.0000** | **1.42** | **0.0003** |  |
| 169166 | SNX31 | | 0.80 | | | 0.0027 | | **0.52** | | **0.0004** | **0.58** | **0.0004** |  |
| 197259 | MLKL | | 1.34 | | | 0.0024 | | **2.33** | | **0.0004** | **1.51** | **0.0000** |  |
| 203328 | SUSD3 | | 0.83 | | | 0.1035 | | **0.60** | | **0.0007** | **0.65** | **0.0003** |  |
| 254295 | PHYHD1 | | 0.81 | | | 0.0048 | | **0.66** | | **0.0006** | **0.69** | **0.0000** |  |
| 284252 | KCTD1 | | 1.21 | | | 0.0472 | | **1.91** | | **0.0000** | **1.53** | **0.0000** |  |
| 284340 | CXCL17 | | 1.49 | | | 0.0077 | | **1.76** | | **0.0004** | **1.40** | **0.0002** |  |
| 285755 | PPIL6 | | 0.84 | | | 0.0707 | | **0.54** | | **0.0000** | **0.57** | **0.0000** |  |
| 374569 | ASPG | | 0.82 | | | 0.0341 | | **0.51** | | **0.0007** | **0.45** | **0.0000** |  |
| 388403 | YPEL2 | | 1.64 | | | 0.0015 | | **2.95** | | **0.0005** | **1.52** | **0.0005** |  |
| 389941 | C1QL3 | | 1.04 | | | 0.4374 | | **2.88** | | **0.0000** | **1.54** | **0.0000** |  |
| 404217 | CTXN1 | | 1.32 | | | 0.0029 | | **2.33** | | **0.0004** | **1.82** | **0.0000** |  |
| **G. 103 genes regulated in NZW/BXSB and NZB/W.** | | | | | | | | | | | | |  |
| Human Entrez Gene ID | Human Gene symbol | | | **NZB/W** | | | **NZM2410** | | | | **NZW/BXSB** | |  |
|  |  |  |  | Fold-change | q-value | | Fold-change | | q-value | | Fold-change | q-value |  |
| 113 | ADCY7 | | | **2.49** | **0.0000** | | 2.11 | | 0.0028 | | **1.93** | **0.0000** |  |
| 397 | ARHGDIB | | | **2.13** | **0.0000** | | 1.57 | | 0.0010 | | **1.62** | **0.0000** |  |
| 633 | BGN | | | **1.89** | **0.0000** | | 1.59 | | 0.0337 | | **1.66** | **0.0000** |  |
| 710 | SERPING1 | | | **2.01** | **0.0000** | | 1.30 | | 0.2267 | | **1.53** | **0.0000** |  |
| 917 | CD3G | | | **3.09** | **0.0000** | | 2.53 | | 0.0014 | | **1.77** | **0.0003** |  |
| 962 | CD48 | | | **4.90** | **0.0000** | | 2.40 | | 0.0046 | | **1.85** | **0.0003** |  |
| 1265 | CNN2 | | | **1.68** | **0.0000** | | 1.42 | | 0.0194 | | **1.61** | **0.0000** |  |
| 1278 | COL1A2 | | | **2.16** | **0.0004** | | 1.43 | | 0.2100 | | **2.89** | **0.0000** |  |
| 1282 | COL4A1 | | | **1.59** | **0.0000** | | 1.13 | | 0.1691 | | **1.76** | **0.0000** |  |
| 1284 | COL4A2 | | | **1.82** | **0.0000** | | 0.94 | | 0.3455 | | **1.85** | **0.0000** |  |
| 1291 | COL6A1 | | | **1.74** | **0.0007** | | 1.08 | | 0.4600 | | **2.21** | **0.0000** |  |
| 1326 | MAP3K8 | | | **1.72** | **0.0000** | | 1.38 | | 0.0040 | | **1.45** | **0.0000** |  |
| 1513 | CTSK | | | **1.56** | **0.0000** | | 1.25 | | 0.1130 | | **1.58** | **0.0000** |  |
| 1545 | CYP1B1 | | | **2.03** | **0.0000** | | 1.63 | | 0.0013 | | **1.56** | **0.0000** |  |
| 1633 | DCK | | | **1.54** | **0.0004** | | 1.34 | | 0.0009 | | **1.63** | **0.0003** |  |
| 1950 | EGF | | | **0.50** | **0.0000** | | 0.68 | | 0.0212 | | **0.55** | **0.0000** |  |
| 2014 | EMP3 | | | **2.08** | **0.0000** | | 1.58 | | 0.0043 | | **1.87** | **0.0000** |  |
| 2152 | F3 | | | **1.68** | **0.0006** | | 2.43 | | 0.0012 | | **1.50** | **0.0003** |  |
| 2153 | F5 | | | **0.57** | **0.0000** | | 0.52 | | 0.0040 | | **0.70** | **0.0000** |  |
| 2200 | FBN1 | | | **1.76** | **0.0004** | | 1.19 | | 0.3166 | | **2.27** | **0.0000** |  |
| 2214 | FCGR3A | | | **3.87** | **0.0000** | | 1.65 | | 0.0015 | | **2.06** | **0.0005** |  |
| 2335 | FN1 | | | **1.99** | **0.0000** | | 1.61 | | 0.0029 | | **1.94** | **0.0000** |  |
| 2819 | GPD1 | | | **0.62** | **0.0000** | | 0.56 | | 0.0012 | | **0.66** | **0.0000** |  |
| 3371 | TNC | | | **1.69** | **0.0000** | | 1.75 | | 0.0139 | | **2.16** | **0.0000** |  |
| 3433 | IFIT2 | | | **1.75** | **0.0007** | | 2.35 | | 0.0032 | | **2.08** | **0.0000** |  |
| 3459 | IFNGR1 | | | **1.52** | **0.0000** | | 1.63 | | 0.0015 | | **1.50** | **0.0000** |  |
| 3587 | IL10RA | | | **1.54** | **0.0000** | | 1.28 | | 0.0378 | | **1.50** | **0.0000** |  |
| 3669 | ISG20 | | | **2.42** | **0.0000** | | 1.51 | | 0.0049 | | **1.41** | **0.0000** |  |
| 3684 | ITGAM | | | **2.30** | **0.0000** | | 2.01 | | 0.0012 | | **2.45** | **0.0000** |  |
| 3687 | ITGAX | | | **2.55** | **0.0000** | | 1.61 | | 0.0032 | | **1.58** | **0.0003** |  |
| 3689 | ITGB2 | | | **3.88** | **0.0000** | | 2.29 | | 0.0010 | | **2.29** | **0.0000** |  |
| 3795 | KHK | | | **0.62** | **0.0000** | | 0.57 | | 0.0010 | | **0.70** | **0.0000** |  |
| 3855 | KRT7 | | | **1.42** | **0.0007** | | 1.75 | | 0.0032 | | **1.51** | **0.0005** |  |
| 3880 | KRT19 | | | **1.79** | **0.0006** | | 2.53 | | 0.0144 | | **1.90** | **0.0003** |  |
| 3936 | LCP1 | | | **3.48** | **0.0000** | | 1.79 | | 0.0050 | | **2.08** | **0.0000** |  |
| 3965 | LGALS9 | | | **1.63** | **0.0000** | | 1.22 | | 0.0184 | | **1.61** | **0.0000** |  |
| 4016 | LOXL1 | | | **1.89** | **0.0000** | | 1.55 | | 0.0129 | | **1.69** | **0.0000** |  |
| 4035 | LRP1 | | | **1.50** | **0.0000** | | 1.16 | | 0.2742 | | **1.42** | **0.0000** |  |
| 4082 | MARCKS | | | **1.61** | **0.0006** | | 1.22 | | 0.2389 | | **1.58** | **0.0000** |  |
| 4174 | MCM5 | | | **1.55** | **0.0004** | | 1.35 | | 0.0048 | | **2.47** | **0.0000** |  |
| 4256 | MGP | | | **2.53** | **0.0000** | | 2.21 | | 0.0031 | | **2.41** | **0.0000** |  |
| 4313 | MMP2 | | | **1.66** | **0.0000** | | 1.42 | | 0.0101 | | **1.87** | **0.0000** |  |
| 4627 | MYH9 | | | **1.41** | **0.0000** | | 1.65 | | 0.0013 | | **1.41** | **0.0000** |  |
| 4759 | NEU2 | | | **0.69** | **0.0000** | | 0.77 | | 0.0004 | | **0.66** | **0.0000** |  |
| 4811 | NID1 | | | **1.40** | **0.0000** | | 1.12 | | 0.2598 | | **1.53** | **0.0000** |  |
| 5199 | CFP | | | **2.30** | **0.0000** | | 1.57 | | 0.0109 | | **1.68** | **0.0000** |  |
| 5360 | PLTP | | | **1.96** | **0.0000** | | 1.21 | | 0.1534 | | **1.40** | **0.0000** |  |
| 5698 | PSMB9 | | | **3.02** | **0.0000** | | 2.50 | | 0.0040 | | **1.74** | **0.0007** |  |
| 6001 | RGS10 | | | **2.04** | **0.0000** | | 1.68 | | 0.0013 | | **1.60** | **0.0000** |  |
| 6241 | RRM2 | | | **1.96** | **0.0000** | | 1.64 | | 0.0073 | | **3.84** | **0.0000** |  |
| 6275 | S100A4 | | | **2.53** | **0.0000** | | 1.86 | | 0.0098 | | **2.18** | **0.0000** |  |
| 6404 | SELPLG | | | **1.74** | **0.0000** | | 1.54 | | 0.0162 | | **1.54** | **0.0000** |  |
| 6503 | SLA | | | **2.35** | **0.0000** | | 1.51 | | 0.0032 | | **1.72** | **0.0000** |  |
| 6556 | SLC11A1 | | | **2.26** | **0.0000** | | 1.58 | | 0.0013 | | **2.07** | **0.0000** |  |
| 6584 | SLC22A5 | | | **0.59** | **0.0000** | | 0.69 | | 0.0032 | | **0.69** | **0.0000** |  |
| 6678 | SPARC | | | **1.64** | **0.0000** | | 0.93 | | 0.3754 | | **1.84** | **0.0000** |  |
| 6688 | SPI1 | | | **1.75** | **0.0000** | | 1.34 | | 0.0092 | | **1.53** | **0.0000** |  |
| 6890 | TAP1 | | | **2.03** | **0.0000** | | 2.32 | | 0.0057 | | **1.52** | **0.0000** |  |
| 6941 | TCF19 | | | **1.42** | **0.0000** | | 1.21 | | 0.0474 | | **2.25** | **0.0000** |  |
| 7058 | THBS2 | | | **1.62** | **0.0006** | | 1.73 | | 0.0112 | | **1.59** | **0.0000** |  |
| 7070 | THY1 | | | **2.08** | **0.0000** | | 2.20 | | 0.0043 | | **1.84** | **0.0000** |  |
| 8034 | SLC25A16 | | | **0.63** | **0.0000** | | 0.70 | | 0.0012 | | **0.70** | **0.0000** |  |
| 8477 | GPR65 | | | **2.16** | **0.0000** | | 1.78 | | 0.0029 | | **1.97** | **0.0000** |  |
| 9246 | UBE2L6 | | | **1.90** | **0.0000** | | 1.88 | | 0.0129 | | **1.52** | **0.0005** |  |
| 9641 | IKBKE | | | **1.72** | **0.0000** | | 1.64 | | 0.0012 | | **1.43** | **0.0000** |  |
| 10186 | LHFP | | | **1.73** | **0.0000** | | 1.29 | | 0.1926 | | **1.72** | **0.0000** |  |
| 10434 | LYPLA1 | | | **0.61** | **0.0000** | | 0.69 | | 0.0058 | | **0.70** | **0.0003** |  |
| 10563 | CXCL13 | | | **6.22** | **0.0000** | | 1.78 | | 0.0079 | | **2.50** | **0.0000** |  |
| 10630 | PDPN | | | **1.43** | **0.0004** | | 1.98 | | 0.0012 | | **1.65** | **0.0000** |  |
| 10875 | FGL2 | | | **2.87** | **0.0000** | | 2.23 | | 0.0016 | | **1.91** | **0.0000** |  |
| 10970 | CKAP4 | | | **1.45** | **0.0000** | | 1.41 | | 0.0032 | | **1.41** | **0.0000** |  |
| 11082 | ESM1 | | | **0.43** | **0.0000** | | 0.50 | | 0.0027 | | **0.51** | **0.0000** |  |
| 11117 | EMILIN1 | | | **1.57** | **0.0004** | | 1.17 | | 0.3616 | | **1.57** | **0.0000** |  |
| 11167 | FSTL1 | | | **1.61** | **0.0000** | | 1.06 | | 0.4703 | | **2.23** | **0.0000** |  |
| 27128 | CYTH4 | | | **3.13** | **0.0000** | | 2.15 | | 0.0070 | | **1.98** | **0.0000** |  |
| 29992 | PILRA | | | **1.50** | **0.0000** | | 1.11 | | 0.2517 | | **1.73** | **0.0000** |  |
| 50486 | G0S2 | | | **0.63** | **0.0007** | | 0.68 | | 0.0066 | | **0.61** | **0.0000** |  |
| 51155 | HN1 | | | **1.96** | **0.0000** | | 1.95 | | 0.0032 | | **1.91** | **0.0000** |  |
| 51166 | AADAT | | | **0.64** | **0.0003** | | 0.71 | | 0.0170 | | **0.63** | **0.0000** |  |
| 51284 | TLR7 | | | **1.50** | **0.0000** | | 1.46 | | 0.0011 | | **1.61** | **0.0000** |  |
| 53827 | FXYD5 | | | **2.15** | **0.0000** | | 1.48 | | 0.0028 | | **1.79** | **0.0000** |  |
| 54069 | MIS18A | | | **1.47** | **0.0000** | | 1.43 | | 0.0036 | | **1.43** | **0.0000** |  |
| 55194 | FAM176B | | | **1.51** | **0.0000** | | 1.37 | | 0.0511 | | **1.49** | **0.0000** |  |
| 55251 | PCMTD2 | | | **0.68** | **0.0004** | | 0.72 | | 0.0009 | | **0.71** | **0.0000** |  |
| 56925 | LXN | | | **1.54** | **0.0000** | | 1.77 | | 0.0029 | | **1.56** | **0.0000** |  |
| 64005 | MYO1G | | | **1.85** | **0.0000** | | 1.59 | | 0.0016 | | **1.48** | **0.0003** |  |
| 64115 | C10orf54 | | | **1.72** | **0.0000** | | 1.40 | | 0.0092 | | **1.46** | **0.0000** |  |
| 64135 | IFIH1 | | | **1.58** | **0.0000** | | 1.91 | | 0.0015 | | **1.75** | **0.0000** |  |
| 64581 | CLEC7A | | | **2.30** | **0.0000** | | 2.33 | | 0.0048 | | **1.60** | **0.0000** |  |
| 79783 | C7orf10 | | | **0.48** | **0.0000** | | 0.55 | | 0.0017 | | **0.59** | **0.0000** |  |
| 81552 | VOPP1 | | | **1.47** | **0.0000** | | 1.91 | | 0.0016 | | **1.45** | **0.0000** |  |
| 90865 | IL33 | | | **2.56** | **0.0000** | | 1.76 | | 0.0032 | | **2.03** | **0.0000** |  |
| 93978 | CLEC6A | | | **1.84** | **0.0000** | | 2.62 | | 0.0232 | | **1.81** | **0.0000** |  |
| 146722 | CD300LF | | | **1.55** | **0.0000** | | 1.19 | | 0.0366 | | **1.40** | **0.0000** |  |
| 171425 | CLYBL | | | **0.60** | **0.0000** | | 0.65 | | 0.0012 | | **0.69** | **0.0000** |  |
| 171586 | ABHD3 | | | **0.50** | **0.0000** | | 0.60 | | 0.0024 | | **0.68** | **0.0000** |  |
| 266812 | NAP1L5 | | | **0.54** | **0.0000** | | 0.54 | | 0.0046 | | **0.63** | **0.0000** |  |
| 286827 | TRIM59 | | | **1.42** | **0.0000** | | 1.15 | | 0.0337 | | **1.72** | **0.0003** |  |
| 375387 | LRRC33 | | | **1.82** | **0.0000** | | 1.41 | | 0.0303 | | **1.51** | **0.0003** |  |
| 388646 | GBP7 | | | **2.05** | **0.0000** | | 2.16 | | 0.0040 | | **1.62** | **0.0000** |  |
| 641700 | ECSCR | | | **1.69** | **0.0000** | | 1.30 | | 0.0194 | | **1.53** | **0.0000** |  |
| 653361 | NCF1 | | | **1.41** | **0.0000** | | 1.42 | | 0.0028 | | **1.40** | **0.0000** |  |
| 1.01E+08 | CASP12 | | | **1.50** | **0.0004** | | 2.23 | | 0.0016 | | **1.52** | **0.0000** |  |
