# Supplementary material for: Comparative Transcriptional Profiling of 3 Murine Models of SLE Nephritis Reveals Both Unique and Shared Regulatory Networks
Source: PLoS One. 2013 Oct 22;8(10):e77489. doi: 10.1371/journal.pone.0077489 (PMC3805607; doi:10.1371/journal.pone.0077489)
Supplement: Table S2 — Stat3 regulated genes in NZB/W, NZM2410, NZW/BXSB and in human LN renal biopsies. (DOCX) [file pone.0077489.s002.docx]

**Table S2:** Stat3 regulated genes in NZB/W, NZM2410, NZW/BXSB and in human LN renal biopsies (1).

ns = not significantly regulated; #N/A = not on the Human Affymetrix array or not passing the expression cut-off. Genes discordantly regulated in the 3 mouse models and in humans are highlighted in italic - bold.

| Gene ID | Gene symbol | NZB/W | | NZM2410 | | NZW/BXSB | | Human LN  glomeruli | | Human LN tubulointerstitium | |
| --- | --- | --- | --- | --- | --- | --- | --- | --- | --- | --- | --- |
|  |  | Fold-change | q-value | Fold-change | q-value | Fold-change | q-value | Fold-change | q-value | Fold-change | q-value |
| 27063 | ANKRD1 | 3.16 | 0.000 | 7.33 | 0.000 | 2.09 | 0.000 | ***0.92*** | ***0.005*** | #N/A | #N/A |
| 301 | ANXA1 | 2.20 | 0.000 | 3.59 | 0.000 | 2.48 | 0.000 | 1.27 | 0.019 | 2.00 | 0.000 |
| 302 | ANXA2 | 1.84 | 0.000 | 3.51 | 0.000 | 2.08 | 0.000 | ***0.93*** | ***0.029*** | 1.89 | 0.000 |
| 306 | ANXA3 | 2.31 | 0.000 | 6.10 | 0.000 | 2.24 | 0.000 | ns | ns | 1.64 | 0.000 |
| 558 | AXL | 2.14 | 0.000 | 1.70 | 0.000 | 1.71 | 0.000 | ns | ns | 1.22 | 0.000 |
| 602 | BCL3 | 2.17 | 0.000 | 2.53 | 0.000 | 2.07 | 0.000 | ***0.88*** | ***0.007*** | ***0.79*** | ***0.000*** |
| 330 | BIRC3 | 1.89 | 0.000 | 2.49 | 0.000 | 1.78 | 0.000 | 1.52 | 0.007 | ns | ns |
| 332 | BIRC5 | 2.11 | 0.000 | 2.00 | 0.000 | 3.23 | 0.000 | ns | ns | ns | ns |
| 684 | BST2 | 2.07 | 0.000 | 2.42 | 0.000 | 1.79 | 0.000 | ns | ns | 3.78 | 0.000 |
| 718 | C3 | 4.16 | 0.000 | 7.57 | 0.000 | 5.47 | 0.000 | 2.17 | 0.014 | 1.96 | 0.005 |
| 6347 | CCL2 | 3.45 | 0.000 | 3.11 | 0.000 | 3.71 | 0.000 | ns | ns | ns | ns |
| 6352 | CCL5 | 3.27 | 0.000 | 10.59 | 0.001 | 2.70 | 0.000 | 2.89 | 0.000 | 1.81 | 0.001 |
| 729230 | CCR2 | 2.18 | 0.000 | 3.54 | 0.001 | 2.54 | 0.000 | 1.88 | 0.002 | ns | ns |
| 1234 | CCR5 | 1.84 | 0.000 | 1.93 | 0.000 | 2.08 | 0.000 | 2.00 | 0.000 | ns | ns |
| 960 | CD44 | 4.57 | 0.000 | 14.60 | 0.000 | 3.51 | 0.000 | 2.73 | 0.000 | 1.25 | 0.004 |
| 983 | CDK1=cdc2 | 1.77 | 0.000 | 1.66 | 0.000 | 3.05 | 0.000 | 1.27 | 0.001 | ns | ns |
| 1163 | CKS1B | 2.17 | 0.000 | 2.24 | 0.000 | 2.31 | 0.000 | #N/A | #N/A | #N/A | #N/A |
| 1364 | CLDN4 | 1.97 | 0.000 | 4.31 | 0.000 | 1.81 | 0.000 | ***0.76*** | ***0.018*** | ns | ns |
| 1356 | CP | 4.26 | 0.000 | 8.94 | 0.000 | 2.73 | 0.000 | ns | ns | ns | ns |
| 1438 | CSF2RA | 1.84 | 0.000 | 1.63 | 0.001 | 1.56 | 0.000 | #N/A | #N/A | #N/A | #N/A |
| 1509 | CTSD | 1.56 | 0.000 | 2.44 | 0.000 | 1.57 | 0.000 | ***0.65*** | ***0.001*** | ns | ns |
| 1520 | CTSS | 5.22 | 0.000 | 4.71 | 0.000 | 2.61 | 0.000 | 4.47 | 0.000 | 1.86 | 0.000 |
| 3627 | CXCL10 | 2.44 | 0.000 | 4.36 | 0.000 | 3.58 | 0.001 | 1.85 | 0.003 | 3.01 | 0.000 |
| 1536 | CYBB | 3.49 | 0.000 | 2.79 | 0.000 | 2.30 | 0.000 | 4.07 | 0.000 | 1.70 | 0.000 |
| 2149 | F2R | 2.50 | 0.000 | 2.40 | 0.001 | 2.11 | 0.000 | ns | ns | 1.24 | 0.000 |
| 2209 | FCGR1A | 1.52 | 0.000 | 1.75 | 0.000 | 1.51 | 0.000 | #N/A | #N/A | #N/A | #N/A |
| 2266 | FGG | 4.25 | 0.000 | 6.14 | 0.000 | 2.74 | 0.000 | ***0.20*** | ***0.030*** | ***0.09*** | ***0.043*** |
| 2358 | FPR2 | 5.48 | 0.000 | 2.88 | 0.000 | 2.51 | 0.000 | 1.38 | 0.001 | ***0.87*** | ***0.000*** |
| 3055 | HCK | 3.76 | 0.000 | 2.29 | 0.000 | 2.15 | 0.000 | 6.62 | 0.000 | 1.37 | 0.000 |
| 3383 | ICAM1 | 2.59 | 0.000 | 3.78 | 0.000 | 1.70 | 0.000 | ns | ns | ns | ns |
| 3659 | IRF1 | 1.66 | 0.000 | 2.13 | 0.000 | 1.47 | 0.000 | ns | ns | ns | ns |
| 3665 | IRF7 | 2.47 | 0.000 | 2.80 | 0.001 | 2.41 | 0.000 | 3.87 | 0.000 | 1.66 | 0.000 |
| 10379 | IRF9 | 1.97 | 0.000 | 2.33 | 0.000 | 1.51 | 0.000 | 1.61 | 0.000 | 2.65 | 0.000 |
| 3875 | KRT18 | 1.90 | 0.000 | 2.63 | 0.001 | 1.52 | 0.001 | ***0.58*** | ***0.000*** | ns | ns |
| 3934 | LCN2 | 21.72 | 0.000 | 43.71 | 0.000 | 22.84 | 0.000 | ns | ns | ns | ns |
| 3958 | LGALS3 | 1.95 | 0.000 | 3.63 | 0.000 | 3.10 | 0.000 | #N/A | #N/A | #N/A | #N/A |
| 3959 | LGALS3BP | 2.62 | 0.000 | 2.78 | 0.000 | 2.19 | 0.000 | 1.55 | 0.000 | 2.19 | 0.000 |
| 9516 | LITAF | 1.62 | 0.000 | 2.21 | 0.000 | 1.79 | 0.000 | 1.14 | 0.047 | 1.18 | 0.002 |
| 4323 | MMP14 | 1.64 | 0.000 | 1.86 | 0.001 | 1.68 | 0.000 | ***0.92*** | ***0.007*** | ***0.86*** | ***0.000*** |
| 64332 | NFKBIZ | 2.19 | 0.000 | 3.84 | 0.000 | 1.71 | 0.000 | #N/A | #N/A | #N/A | #N/A |
| 9180 | OSMR | 2.14 | 0.000 | 3.02 | 0.000 | 1.74 | 0.000 | ns | ns | 1.14 | 0.029 |
| 5341 | PLEK | 3.05 | 0.000 | 2.36 | 0.001 | 1.91 | 0.000 | 2.81 | 0.000 | ns | ns |
| 5579 | PRKCB | 1.95 | 0.000 | 1.66 | 0.000 | 1.51 | 0.000 | 2.02 | 0.000 | ns | ns |
| 5696 | PSMB8 | 3.17 | 0.000 | 4.35 | 0.000 | 2.06 | 0.000 | 1.67 | 0.000 | 2.74 | 0.000 |
| 5788 | PTPRC | 6.03 | 0.000 | 3.06 | 0.001 | 2.47 | 0.000 | 6.07 | 0.000 | 2.03 | 0.000 |
| 29108 | PYCARD | 1.97 | 0.000 | 1.53 | 0.000 | 1.87 | 0.000 | 4.14 | 0.000 | 1.56 | 0.000 |
| 5971 | RELB | 1.80 | 0.000 | 3.11 | 0.000 | 1.78 | 0.000 | ns | ns | ***0.87*** | ***0.001*** |
| 58480 | RHOU | 1.58 | 0.001 | 3.14 | 0.000 | 1.46 | 0.000 | #N/A | #N/A | #N/A | #N/A |
| 6382 | SDC1 | 1.49 | 0.000 | 1.99 | 0.000 | 1.42 | 0.000 | 0.47 | 0.000 | ***0.67*** | ***0.000*** |
| 12 | SERPINA3 | 2.50 | 0.000 | 4.30 | 0.000 | 3.27 | 0.000 | 0.23 | 0.045 | ns | ns |
| 5269 | SERPINB6 | 1.81 | 0.000 | 2.71 | 0.000 | 1.59 | 0.000 | 0.90 | 0.034 | ***0.90*** | ***0.001*** |
| 6518 | SLC2A5 | 0.69 | 0.001 | 0.47 | 0.000 | 0.61 | 0.000 | 0.62 | 0.000 | ***0.79*** | ***0.012*** |
| 10568 | SLC34A2 | 2.35 | 0.000 | 4.83 | 0.000 | 2.05 | 0.000 | #N/A | #N/A | #N/A | #N/A |
| 9021 | SOCS3 | 5.20 | 0.000 | 7.62 | 0.000 | 2.74 | 0.000 | ***0.78*** | ***0.000*** | ***0.81*** | ***0.000*** |
| 7040 | TGFB1 | 1.96 | 0.000 | 1.64 | 0.000 | 1.56 | 0.000 | ns | ns | ***0.88*** | ***0.000*** |
| 7076 | TIMP1 | 2.79 | 0.000 | 4.99 | 0.000 | 4.45 | 0.000 | 1.64 | 0.000 | 2.73 | 0.000 |
| 7097 | TLR2 | 2.03 | 0.000 | 3.24 | 0.000 | 1.72 | 0.000 | 4.32 | 0.000 | 1.22 | 0.002 |
| 7128 | TNFAIP3 | 1.64 | 0.000 | 2.66 | 0.000 | 1.56 | 0.000 | 1.19 | 0.047 | ***0.85*** | ***0.008*** |
| 7412 | VCAM1 | 2.75 | 0.000 | 10.71 | 0.000 | 2.72 | 0.000 | 1.40 | 0.030 | 1.85 | 0.000 |
| 7431 | VIM | 2.19 | 0.000 | 2.92 | 0.000 | 2.56 | 0.000 | ns | ns | 2.37 | 0.000 |

*(1) Reference: Cross-species transcriptional network analysis defines shared inflammatory responses in murine and human lupus nephritis. Berthier CC et al., J Immunol, 2012, 189(2):988-1001. PMID: 22723521.*
