# Supplementary material for: Comparative Transcriptional Profiling of 3 Murine Models of SLE Nephritis Reveals Both Unique and Shared Regulatory Networks
Source: PLoS One. 2013 Oct 22;8(10):e77489. doi: 10.1371/journal.pone.0077489 (PMC3805607; doi:10.1371/journal.pone.0077489)
Supplement: Table S3 — Comparison of microarray data and qPCR data. (DOC) [file pone.0077489.s003.doc]

**Table S3:** Comparison of microarray data and qPCR data.

nc: not on the Affymetrix Brain Array annotation version 10. np: not passing the Affymetrix negative control cut-off. ns.: not significant.

Discordant genes between microarray and PCR are in **bold**.

| **GeneID** | **Gene Symbol** | **NZB/W** | | | | **NZW/BXSB** | | | | | | **NZM2410** | | | |
| --- | --- | --- | --- | --- | --- | --- | --- | --- | --- | --- | --- | --- | --- | --- | --- |
|  |  | Arrays | | RT-PCR | | Arrays | | | | RT-PCR | | Arrays | | RT-PCR | |
|  |  | neph vs 16w | | neph vs 6+16w | | neph vs 8+17w | | neph vs 8w | | neph vs 8w | | neph vs 8w | | neph vs 8w | |
|  |  | **Fold-change** | **q-value** | **Fold-change** | **p-value** | **Fold-change** | **q-value** | **Fold-change** | **q-value** | **Fold-change** | **p-value** | **Fold-change** | **q-value** | **Fold-change** | **p-value** |
| 11504 | Adamts1 | 2.13 | 0.0009 | 1.95 | 0.049 | 1.47 | 0.0084 | 1.95 | 0.0000 | 1.58 | ns | 4.91 | 0.0000 | 3.44 | 0.008 |
| 107765 | Ankrd1 | 3.16 | 0.0000 | 6.88 | 0.000 | 2.09 | 0.0000 | 2.45 | 0.0000 | 2.83 | 0.000 | 7.33 | 0.0000 | 6.17 | 0.001 |
| 11745 | Anxa3 | 2.31 | 0.0000 | 1.69 | ns | 2.24 | 0.0000 | 2.15 | 0.0000 | 5.00 | 0.000 | 6.10 | 0.0000 | 3.74 | 0.003 |
| 11865 | Arntl | 1.34 | 0.2221 | **0.39** | 0.043 | 1.27 | 0.0959 | 2.29 | 0.0000 | 8.25 | 0.000 | 1.64 | 0.0366 | 2.92 | 0.002 |
| 226641 | Atf6 | 0.98 | 0.3334 | **0.54** | 0.008 | 1.00 | ns. | 0.98 | ns | 0.67 | ns | 0.98 | 0.3022 | **2.70** | 0.001 |
| 11799 | Birc5 | 2.11 | 0.0000 | 1.72 | ns | 3.23 | 0.0000 | 2.84 | 0.0437 | 2.43 | 0.011 | 2.00 | 0.0000 | 3.61 | 0.010 |
| 12259 | C1qa | 4.87 | 0.0000 | 5.23 | 0.000 | 2.70 | 0.0000 | 3.10 | 0.0000 | 4.99 | 0.000 | 3.51 | 0.0000 | 1.61 | 0.033 |
| 12266 | C3 | 4.16 | 0.0000 | 2.52 | 0.005 | 5.47 | 0.0000 | 4.48 | 0.0060 | 5.87 | 0.000 | 7.57 | 0.0004 | 6.12 | 0.006 |
| 20290 | Ccl1 | 1.15 | 0.2401 | 1.45 | ns | 0.96 | 0.1808 | 0.96 | ns | **2.91** | 0.018 | 0.93 | 0.1926 | 0.81 | ns |
| 20292 | Ccl11 | np | np | 1.58 | ns | 1.21 | 0.0544 | 1.17 | ns | 1.24 | ns | np | np | 2.86 | ns |
| 20293 | Ccl12 | 3.45 | 0.0000 | 10.91 | 0.000 | 3.71 | 0.0003 | 3.51 | 0.0414 | 9.71 | 0.000 | 3.11 | 0.0000 | 2.68 | 0.008 |
| 20295 | Ccl17 | 1.03 | 0.4238 | 1.32 | ns | 1.21 | 0.0043 | 1.15 | ns | **2.79** | 0.027 | 1.11 | 0.3540 | 1.06 | ns |
| 24047 | CCL19 | nc | nc | 3.10 | 0.000 | nc | nc | nc | nc | 1.62 | ns | nc | nc | 2.06 | ns |
| 20296 | Ccl2 | 1.78 | 0.0007 | 4.65 | 0.001 | 2.02 | 0.0000 | 1.82 | 0.0088 | 7.46 | 0.001 | 2.83 | 0.0000 | 5.94 | 0.000 |
| 20297 | Ccl20 | 1.26 | 0.0041 | 37.08 | 0.000 | 1.37 | 0.0186 | 1.24 | ns | 1.29 | ns | 1.67 | 0.0007 | 1.40 | ns |
| 20299 | Ccl22 | 0.98 | 0.3550 | 1.85 | ns | 0.98 | ns. | 0.97 | ns | 1.96 | ns | 0.97 | 0.4009 | 0.53 | ns |
| 56838 | Ccl28 | nc | nc | 2.39 | ns | nc | nc | nc | nc | 0.39 | 0.020 | nc | nc | 4.78 | 0.052 |
| 20302 | Ccl3 | 1.67 | 0.0000 | 20.82 | 0.000 | 1.19 | 0.0357 | 1.30 | 0.0414 | 5.17 | 0.002 | np | np | 2.77 | 0.005 |
| 20303 | Ccl4 | 1.29 | 0.0207 | 16.36 | 0.000 | 1.18 | 0.0582 | 1.26 | ns | 4.66 | 0.003 | 1.23 | 0.1053 | **5.35** | 0.012 |
| 20304 | Ccl5 | 3.27 | 0.0000 | 13.32 | 0.000 | 2.70 | 0.0000 | 2.68 | 0.0086 | 7.13 | 0.000 | 10.59 | 0.0008 | 9.40 | 0.003 |
| 20307 | Ccl8 | 7.64 | 0.0000 | 5.63 | 0.004 | 2.21 | 0.0032 | 2.37 | 0.0566 | 1.49 | ns | 2.76 | 0.0089 | **0.72** | NS |
| 20308 | Ccl9 | 2.90 | 0.0000 | 7.83 | 0.000 | 2.29 | 0.0000 | 2.15 | 0.0023 | 4.61 | 0.000 | 2.66 | 0.0006 | 2.48 | 0.004 |
| 12768 | Ccr1 | 1.32 | 0.0216 | 3.62 | 0.002 | 1.53 | 0.0000 | 1.41 | 0.0159 | 8.66 | 0.000 | 1.57 | 0.0004 | **0.63** | ns |
| 12777 | Ccr10 | 1.06 | 0.3298 | **3.02** | 0.005 | 1.04 | 0.3131 | 1.10 | ns | **2.91** | 0.018 | 1.03 | 0.4734 | 1.60 | ns |
| 12772 | Ccr2 | 2.18 | 0.0000 | 6.68 | 0.000 | 2.54 | 0.0000 | 2.24 | 0.0000 | 3.83 | 0.012 | 3.54 | 0.0006 | 1.99 | ns |
| 12774 | Ccr5 | 1.84 | 0.0000 | 13.80 | 0.000 | 2.08 | 0.0000 | 1.98 | 0.0056 | 5.07 | 0.000 | 1.93 | 0.0000 | 2.46 | ns |
| 12458 | Ccr6 | 1.48 | 0.0006 | 8.62 | 0.000 | 1.00 | ns. | 1.05 | ns | 1.35 | ns | 1.04 | 0.3922 | 1.07 | ns |
| 12775 | Ccr7 | 1.25 | 0.0023 | 3.03 | 0.000 | 1.11 | 0.2259 | 1.08 | ns | 2.08 | ns | np | np | 0.98 | ns |
| 12776 | Ccr8 | np | np | 8.73 | 0.000 | np | np | np | np | 3.73 | ns | np | np | 1.02 | ns |
| 12475 | Cd14 | 4.19 | 0.0000 | 6.88 | 0.000 | 3.08 | 0.0000 | 3.37 | 0.0000 | 5.70 | 0.000 | 9.53 | 0.0000 | 7.75 | 0.001 |
| 60533 | Cd274 | 1.50 | 0.0053 | 1.15 | ns | 1.04 | ns. | 1.03 | ns. | **1.60** | 0.046 | 1.26 | 0.0042 | **0.83** | ns |
| 12501 | Cd3e | 1.34 | 0.0194 | 3.45 | 0.008 | 0.98 | 0.2922 | 1.02 | ns. | **4.92** | 0.000 | 1.14 | 0.1491 | **2.55** | 0.010 |
| 21939 | Cd40 | 1.30 | 0.0013 | 2.77 | 0.000 | 1.26 | 0.0000 | 1.21 | 0.0288 | 2.28 | 0.023 | 1.53 | 0.0004 | **0.80** | ns |
| 12505 | Cd44 | 4.57 | 0.0000 | 1.91 | ns | 3.51 | 0.0000 | 3.06 | 0.0000 | 3.39 | 0.000 | 14.60 | 0.0000 | 4.22 | 0.008 |
| 23833 | Cd52 | 5.45 | 0.0000 | 11.20 | 0.000 | 2.52 | 0.0000 | 2.65 | 0.0061 | 4.75 | 0.000 | 3.12 | 0.0005 | 2.60 | ns |
| 12514 | Cd68 | 3.37 | 0.0000 | 6.71 | 0.000 | 2.44 | 0.0000 | 2.43 | 0.0021 | 4.17 | 0.000 | 4.75 | 0.0000 | 3.16 | 0.000 |
| 12517 | Cd72 | 4.10 | 0.0000 | 4.55 | 0.001 | 2.42 | 0.0000 | 2.68 | 0.0048 | 4.19 | 0.001 | 1.88 | 0.0009 | 1.35 | ns |
| 26887 | Chst4 | np | np | 3.17 | 0.021 | np | np | np | np | 0.79 | ns | np | np | 1.41 | ns |
| 26888 | Clec4a2 | 2.99 | 0.0000 | 8.08 | 0.000 | 1.66 | 0.0000 | 1.58 | 0.0073 | 3.93 | 0.001 | 1.86 | 0.0000 | 1.44 | ns |
| 73149 | Clec4a3 | 8.18 | 0.0000 | 13.15 | 0.000 | 2.46 | 0.0000 | 3.09 | 0.0029 | 5.78 | 0.000 | 3.55 | 0.0000 | 2.16 | 0.000 |
| 56619 | Clec4e | 1.39 | 0.0031 | 58.79 | 0.000 | 1.40 | 0.0000 | 1.43 | 0.0021 | 10.00 | 0.000 | np | np | 65.27 | 0.000 |
| 56620 | Clec4n | 1.84 | 0.0000 | 17.48 | 0.000 | 1.81 | 0.0000 | 1.72 | 0.0000 | 8.46 | ns | 2.62 | 0.0232 | 3.25 | 0.000 |
| 12753 | Clock | 0.92 | 0.1464 | **0.65** | 0.046 | 0.96 | 0.2466 | 1.05 | ns | 0.72 | ns | 1.14 | 0.1691 | **3.05** | 0.011 |
| 69634 | Clybl | 0.60 | 0.0000 | 0.23 | 0.029 | 0.69 | 0.0000 | 0.73 | 0.0086 | 0.38 | 0.002 | 0.65 | 0.0012 | **1.07** | ns |
| 12843 | Col1a2 | 2.16 | 0.0004 | 1.48 | ns | 2.89 | 0.0000 | 2.07 | 0.0000 | 2.06 | 0.007 | 1.43 | 0.2100 | **0.56** | 0.008 |
| 12721 | Coro1a | 5.26 | 0.0000 | 5.01 | 0.000 | 2.44 | 0.0000 | 2.50 | 0.0000 | 3.30 | 0.000 | 2.93 | 0.0000 | 1.46 | ns |
| 226139 | Cox15 | 0.63 | 0.0000 | 0.50 | 0.008 | 0.84 | 0.0011 | 0.80 | 0.0088 | 0.37 | 0.000 | 0.71 | 0.0007 | **0.94** | ns |
| 12952 | Cry1 | 1.14 | 0.3928 | 1.11 | ns | 1.43 | 0.0095 | 1.63 | 0.0154 | 1.45 | ns | 1.73 | 0.1163 | 3.38 | 0.004 |
| 12953 | Cry2 | 0.83 | 0.0258 | 0.44 | 0.002 | 0.90 | 0.1065 | 0.84 | 0.0689 | 0.35 | 0.001 | 0.82 | 0.0073 | 0.84 | ns |
| 12977 | Csf1 | 1.49 | 0.0000 | 2.52 | 0.008 | 1.34 | 0.0000 | 1.32 | 0.0154 | 1.44 | ns | 2.05 | 0.0000 | 9.11 | 0.001 |
| 13040 | Ctss | 5.22 | 0.0000 | 8.38 | 0.000 | 2.61 | 0.0000 | 3.38 | 0.0000 | 6.39 | 0.000 | 4.71 | 0.0000 | 2.54 | 0.001 |
| 20312 | Cx3cl1 | 1.48 | 0.0145 | 1.89 | ns | 1.47 | 0.0000 | 1.42 | 0.0057 | **0.98** | ns | 1.77 | 0.0000 | **0.73** | ns |
| 13051 | Cx3cr1 | 1.59 | 0.0006 | 4.15 | 0.000 | 1.63 | 0.0000 | 1.55 | 0.0000 | 2.06 | 0.023 | 2.24 | 0.0000 | 2.47 | 0.001 |
| 15945 | Cxcl10 | 2.44 | 0.0004 | 2.52 | 0.002 | 3.58 | 0.0006 | 3.83 | 0.0502 | 3.14 | 0.004 | 4.36 | 0.0000 | **1.07** | ns |
| 56066 | Cxcl11 | 1.31 | 0.0128 | 5.05 | 0.000 | 1.17 | 0.0094 | 1.16 | ns | **2.60** | 0.004 | 1.15 | 0.2065 | 0.96 | ns |
| 20315 | Cxcl12 | 0.87 | 0.0492 | 0.78 | ns | 0.76 | 0.0005 | 0.88 | 0.0689 | 0.95 | ns | 0.68 | 0.0179 | 0.73 | ns |
| 55985 | Cxcl13 | 6.22 | 0.0000 | 62.17 | 0.000 | 2.50 | 0.0000 | 2.72 | 0.0112 | 16.12 | 0.000 | 1.78 | 0.0079 | 3.33 | 0.008 |
| 66102 | Cxcl16 | 1.69 | 0.0000 | 2.88 | 0.000 | 1.98 | 0.0000 | 1.72 | 0.0000 | 1.98 | 0.004 | 2.23 | 0.0000 | **1.16** | ns |
| 17329 | Cxcl9 | 2.43 | 0.0008 | 4.07 | 0.001 | 1.71 | 0.0129 | 1.70 | ns | **3.97** | 0.002 | 1.63 | 0.0474 | **0.84** | ns |
| 12766 | Cxcr3 | 1.47 | 0.0021 | 7.14 | 0.000 | 1.16 | 0.0325 | 1.16 | ns | **3.10** | 0.004 | 1.44 | 0.0684 | 1.91 | 0.056 |
| 12767 | Cxcr4 | 2.25 | 0.0000 | 2.62 | 0.013 | 1.01 | 0.3749 | 1.20 | ns | 2.14 | 0.008 | 1.36 | 0.2100 | 1.07 | ns |
| 12145 | Cxcr5 | np | np | 18.69 | 0.000 | np | np | np | np | 4.18 | ns | np | np | 1.51 | ns |
| 80901 | Cxcr6 | 1.30 | 0.0429 | 2.63 | 0.007 | np | np | np | np | 1.39 | ns | 2.30 | 0.0179 | 1.25 | ns |
| 13058 | Cybb | 3.49 | 0.0000 | 6.55 | 0.000 | 2.30 | 0.0000 | 2.54 | 0.0000 | 3.61 | 0.000 | 2.79 | 0.0000 | 1.56 | .037 |
| 13170 | Dbp | 0.58 | 0.0029 | 0.33 | 0.032 | 0.35 | 0.0151 | 0.23 | 0.0000 | 0.08 | 0.000 | 0.31 | 0.0007 | 0.06 | 0.001 |
| 13198 | Ddit3=CHOP10 | 1.14 | 0.0472 | 0.87 | ns | 0.82 | 0.0905 | 1.00 | ns. | 1.58 | ns | 1.24 | 0.0203 | **0.82** | ns |
| 67819 | Derl1 | 0.88 | 0.0297 | 0.49 | 0.001 | 0.93 | 0.0582 | 0.90 | 0.0519 | **0.46** | 0.015 | 0.89 | 0.0121 | 0.64 | 0.023 |
| 27362 | Dnajb9 | 1.10 | 0.3806 | 1.31 | ns | 0.90 | 0.1808 | 0.98 | ns | 1.23 | ns | 1.28 | 0.0162 | 1.53 | ns |
| 13419 | Dnase1 | 0.36 | 0.0000 | 0.14 | 0.009 | 0.32 | 0.0000 | 0.36 | 0.0315 | 0.19 | 0.014 | 0.11 | 0.0000 | 0.07 | 0.012 |
| 192193 | Edem1 | 1.21 | 0.0004 | 0.90 | ns | 0.99 | ns. | 0.94 | ns | **0.26** | 0.016 | 0.94 | 0.2212 | **0.41** | 0.038 |
| 13645 | Egf | 0.50 | 0.0000 | 0.17 | 0.001 | 0.55 | 0.0000 | 0.63 | 0.0111 | 0.35 | 0.012 | 0.68 | 0.0212 | 0.13 | 0.012 |
| 26918 | Ern2=IRE1b | np |  | 0.51 | ns | 0.92 | 0.0582 | 0.95 | ns | 0.50 | ns | np | np | 2.83 | ns |
| 13982 | Esr1 | 0.92 | 0.1008 | **0.08** | 0.002 | 0.79 | 0.0003 | 0.80 | 0.0078 | 0.09 | 0.000 | 0.86 | 0.0631 | 0.08 | 0.010 |
| 14131 | Fcgr3 | 3.90 | 0.0000 | 3.93 | 0.000 | 2.61 | 0.0000 | 2.69 | 0.0000 | 4.75 | 0.000 | 3.47 | 0.0000 | 3.08 | 0.012 |
| 14173 | Fgf2 | np | np | 0.49 | 0.004 | np | np | np | np | 1.55 | 0.036 | np | np | 0.39 | ns |
| 14182 | Fgfr1 | 1.05 | 0.3856 | **0.54** | 0.008 | 1.00 | ns. | 0.99 | ns | 1.09 | ns | 0.93 | 0.0397 | 0.36 | ns |
| 20371 | Foxp3 | np | np | 10.28 | 0.001 | np | np | np | np | 1.23 | ns | np | np | 1.40 | ns |
| 14289 | Fpr2 | 5.48 | 0.0000 | 18.74 | 0.000 | 2.51 | 0.0000 | 2.62 | 0.0025 | 4.74 | 0.004 | 2.88 | 0.0000 | 3.80 | 0.003 |
| 240921 | Gm4955 | nc | nc | 2.53 | 0.013 | nc | nc | nc | nc | 3.33 | 0.000 | nc | nc | 2.33 | 0.020 |
| 23892 | Grem1 | 1.72 | 0.0013 | 20.29 | 0.000 | 1.16 | 0.1545 | 1.16 | ns | **4.99** | 0.009 | 7.22 | 0.0035 | 30.26 | 0.000 |
| 171283 | Havcr1 | 2.58 | 0.0004 | 3.47 | 0.010 | 2.43 | 0.0000 | 1.99 | 0.0424 | 3.21 | 0.010 | 51.42 | 0.0000 | 87.70 | 0.000 |
| 15201 | Hells | 1.67 | 0.0011 | 1.86 | 0.000 | 3.38 | 0.0000 | 2.89 | 0.0390 | 2.38 | ns | 1.80 | 0.0016 | **0.63** | ns |
| 15251 | Hif1a | 1.05 | 0.4089 | 0.57 | ns | 1.08 | 0.1482 | 1.01 | ns. | 1.06 | ns | 1.13 | 0.1163 | 1.93 | 0.041 |
| 260423 | Hist1h3f | 0.91 | 0.1641 | 2.27 | ns | 0.89 | 0.1016 | 0.87 | ns | **4.74** | 0.004 | 0.82 | 0.1097 | 1.41 | ns |
| 217082 | Hlf | 0.68 | 0.0013 | 0.50 | 0.023 | 0.74 | 0.0032 | 0.72 | 0.0086 | 0.45 | 0.012 | 0.57 | 0.0005 | 0.80 | ns |
| 14828 | Hspa5 | 0.93 | 0.3136 | **0.52** | 0.003 | 1.09 | 0.2952 | 1.05 | ns | 0.58 | ns | 1.35 | 0.0124 | 3.09 | ns |
| 15894 | Icam1 | 2.59 | 0.0000 | 4.28 | 0.002 | 1.70 | 0.0000 | 1.76 | 0.0000 | 2.62 | 0.003 | 3.78 | 0.0000 | 2.79 | 0.006 |
| 54167 | Icos | 1.34 | 0.0016 | 6.43 | 0.000 | 1.08 | 0.2153 | 1.08 | ns | **2.91** | 0.001 | 1.18 | 0.1163 | 1.37 | ns |
| 50723 | Icosl | 1.21 | 0.0071 | 6.19 | 0.000 | 1.07 | 0.0377 | 1.05 | ns | 1.44 | ns | 1.02 | 0.4734 | 0.64 | ns |
| 15978 | IFNG | np | np | 7.49 | 0.002 | np | np | np | np | 2.46 | 0.005 | np | np | 5.85 | 0.009 |
| 16153 | IL10 | np | np | 14.18 | 0.000 | np | np | np | np | 10.77 | 0.000 | np | np | 4.51 | 0.002 |
| 16159 | IL12a=P35 | np | np | 4.28 | 0.045 | np | np | np | np | 2.48 | ns | np | np | 1.15 | ns |
| 16160 | IL12b=P40 | np | np | 3.52 | 0.030 | np | np | np | np | 2.33 | 0.038 | np | np | 0.71 | ns |
| 16171 | Il17a | 1.07 | 0.2460 | 5.67 | ns | 0.94 | 0.1230 | 0.95 | ns | **8.16** | 0.007 | 0.90 | 0.1341 | 2.03 | ns |
| 16173 | Il18 | 1.33 | 0.0623 | 1.20 | ns | 1.09 | 0.3090 | 0.97 | ns | 1.20 | ns | 1.43 | 0.0421 | 4.52 | 0.002 |
| 16176 | IL1b | 2.28 | 0.0000 | 9.42 | 0.000 | 1.36 | 0.0325 | 1.36 | ns | **5.37** | 0.003 | 1.530 | 0.0170 | 2.63 | 0.010 |
| 54448 | Il1f6 | 1.95 | 0.0004 | 485.70 | 0.000 | 2.47 | 0.0000 | 2.14 | 0.0063 | 28.66 | 0.001 | 3.71 | 0.0000 | 229.58 | 0.000 |
| 16178 | Il1r2 | 1.31 | 0.0019 | 4.06 | 0.003 | 1.09 | 0.1292 | 1.08 | ns | **2.92** | 0.001 | 1.06 | 0.3540 | 1.70 | ns |
| 60505 | Il21 | np | np | 25.68 | 0.004 | np | np | np | np | 9.71 | 0.010 | np | np | 3.10 | 0.041 |
| 16189 | IL4 | np | np | 3.38 | ns | 0.91 | 0.0668 | 0.97 | ns | 1.06 | ns | np | np | 0.19 | ns |
| 16193 | IL6 | np | np | 40.05 | 0.000 | 1.99 | 0.0003 | 1.68 | 0.0783 | 13.40 | 0.002 | np | np | 4.59 | 0.004 |
| 16198 | Il9 | 1.02 | 0.4395 | 4.85 | ns | 0.95 | 0.1311 | 0.99 | ns. | **8.17** | 0.000 | 1.01 | ns. | 1.48 | ns |
| 54123 | Irf7 | 2.47 | 0.0000 | 1.88 | ns | 2.41 | 0.0000 | 2.52 | 0.0058 | 2.80 | 0.002 | 2.80 | 0.0009 | 1.89 | 0.030 |
| 16409 | Itgam | 2.30 | 0.0000 | 15.16 | 0.000 | 2.45 | 0.0000 | 2.30 | 0.0000 | 7.56 | 0.000 | 2.01 | 0.0012 | 3.74 | 0.000 |
| 16414 | Itgb2 | 3.88 | 0.0000 | 3.20 | 0.006 | 2.29 | 0.0000 | 2.63 | 0.0000 | not detected | ns | 2.29 | 0.0010 | **1.16** | ns |
| 16476 | Jun | 1.10 | 0.4309 | 1.34 | ns | 0.85 | 0.2382 | 1.00 | ns. | 0.96 | ns | 2.17 | 0.0000 | 6.25 | ns |
| 66809 | Krt20 | 2.37 | 0.0030 | 8.91 | 0.004 | 2.18 | 0.0000 | 2.05 | 0.0120 | 2.34 | ns | 17.06 | 0.0000 | 4.81 | 0.047 |
| 16819 | Lcn2 | 21.72 | 0.0000 | 67.58 | 0.000 | 22.84 | 0.0000 | 16.12 | 0.0023 | 21.19 | 0.001 | 43.71 | 0.0000 | 41.94 | 0.000 |
| 50930 | Light | np | np | 4.68 | 0.001 | np | np | np | np | 2.71 | 0.001 | np | np | 0.86 | ns |
| 16992 | Lta | 1.04 | 0.4252 | **2.56** | 0.004 | 0.98 | 0.2188 | 0.99 | ns | 0.80 | ns | 0.97 | 0.4151 | 1.64 | ns |
| 17084 | Ly86 | 2.86 | 0.0000 | 5.52 | 0.000 | 2.22 | 0.0000 | 2.17 | 0.0000 | 4.63 | 0.000 | 2.46 | 0.0000 | 1.97 | 0.035 |
| 17123 | Madcam1 | np | np | 3.47 | 0.009 | 1.07 | 0.2618 | 1.06 | ns | **2.35** | 0.020 | np | np | 1.40 | ns |
| 17319 | MIF | nc | nc | 0.52 | ns | nc | nc | nc | nc | 0.67 | ns | nc | nc | 1.18 | ns |
| 17345 | Mki67=Ki67 | 2.39 | 0.0014 | 3.56 | 0.025 | 2.72 | 0.0000 | 2.39 | 0.0424 | 3.45 | 0.003 | 1.64 | 0.0000 | 1.73 | ns |
| 17390 | Mmp2 | 1.66 | 0.0000 | 1.37 | ns | 1.87 | 0.0000 | 1.55 | 0.0066 | 2.45 | 0.003 | 1.42 | 0.0101 | 2.83 | 0.035 |
| 17395 | Mmp9 | np | np | 0.53 | 0.038 | 1.07 | 0.0766 | 1.07 | ns | 1.36 | ns | np | np | 1.66 | ns |
| 68774 | Ms4a6d | 6.69 | 0.0000 | 24.45 | 0.000 | 3.02 | 0.0000 | 3.14 | 0.0000 | 11.95 | 0.000 | 3.58 | 0.0000 | 2.89 | 0.017 |
| 18127 | Nos3 | 1.11 | 0.2501 | 0.88 | ns | 1.06 | 0.2153 | 1.10 | ns | 1.20 | ns | 0.77 | 0.0058 | 0.70 | ns |
| 18131 | Notch3 | 1.19 | 0.0297 | **0.48** | ns | 1.41 | 0.0000 | 1.38 | 0.0084 | 3.47 | 0.016 | 1.11 | 0.2267 | 0.81 | ns |
| 18132 | Notch4 | 1.06 | 0.4019 | **0.31** | 0.000 | 0.82 | 0.0021 | 0.81 | 0.0162 | **1.48** | ns | 0.70 | 0.0004 | 0.81 | ns |
| 54631 | Nphs1 | 0.92 | 0.1843 | 0.69 | ns | 0.86 | 0.0302 | 0.96 | ns | 1.05 | ns | 0.64 | 0.0003 | 0.20 | 0.000 |
| 217166 | Nr1d1 | 0.85 | 0.0949 | 0.45 | 0.013 | 0.50 | 0.0037 | 0.52 | 0.0058 | 0.18 | 0.000 | 0.63 | 0.0161 | **1.43** | ns |
| 18534 | Pck1 | 0.60 | 0.0017 | 0.36 | ns | 0.41 | 0.0000 | 0.54 | 0.0061 | 0.23 | 0.002 | 0.61 | 0.0184 | 0.42 | 0.028 |
| 18566 | Pdcd1 | np | np | 29.23 | 0.000 | 1.03 | 0.3782 | 1.05 | ns. | **15.21** | 0.000 | np | np | 3.50 | 0.001 |
| 58205 | Pdcd1lg2 | 1.13 | 0.1308 | **7.13** | 0.001 | np | np | np | np | 6.58 | 0.009 | np. | np | 0.71 | ns |
| 18626 | Per1 | 1.27 | 0.2751 | 1.83 | ns | 1.27 | 0.2670 | 1.31 | ns | 1.30 | ns | 0.90 | 0.3685 | 1.49 | ns |
| 18627 | Per2 | 0.73 | 0.0415 | 0.74 | ns | 0.93 | 0.2259 | 0.79 | ns | 0.34 | 0.000 | 0.75 | 0.0437 | **4.27** | 0.003 |
| 18628 | Per3 | 0.78 | 0.0000 | 0.57 | 0.042 | 0.76 | 0.0083 | 0.67 | 0.0029 | 0.19 | 0.000 | 0.52 | 0.0000 | 0.75 | ns |
| 18722 | PIRA1 | nc | nc | 13.34 | 0.000 | nc | nc | nc | nc | 7.06 | 0.000 | nc | nc | 3.03 | 0.005 |
| 16913 | Psmb8 | 3.17 | 0.0000 | 2.39 | ns | 2.06 | 0.0000 | 2.10 | 0.0017 | 3.70 | 0.000 | 4.35 | 0.0004 | 1.82 | ns |
| 19698 | Relb | 1.80 | 0.0000 | 1.48 | ns | 1.78 | 0.0000 | 1.75 | 0.0000 | 2.43 | 0.038 | 3.11 | 0.0000 | **1.03** | ns |
| 68585 | Rtn4 | 1.19 | 0.0727 | 0.91 | ns | 1.47 | 0.0000 | 1.42 | 0.0061 | 0.74 | ns | 1.91 | 0.0000 | 2.62 | 0.001 |
| 20969 | Sdc1 | 1.49 | 0.0000 | 4.08 | 0.000 | 1.42 | 0.0000 | 1.39 | 0.0187 | 1.80 | 0.012 | 1.99 | 0.0000 | 1.28 | ns |
| 20339 | Sele | np | np | 7.15 | 0.000 | 1.15 | 0.0571 | 1.24 | ns | 2.19 | ns | np | np | 0.76 | ns |
| 20343 | Sell | 1.61 | 0.0004 | 6.40 | 0.001 | 1.05 | 0.3255 | 1.10 | ns | 1.61 | ns | 0.97 | 0.4081 | 1.23 | ns |
| 20344 | Selp | np | np | 8.96 | 0.001 | np | np | np | np | 4.01 | ns | np | np | 3.13 | 0.005 |
| 217847 | Serpina10 | 3.52 | 0.0000 | 6.24 | 0.000 | 4.87 | 0.0000 | 3.74 | 0.0000 | 9.68 | 0.002 | 7.77 | 0.0000 | 7.56 | 0.000 |
| 20715 | Serpina3g | 7.52 | 0.0000 | 18.01 | 0.000 | 2.67 | 0.0000 | 2.83 | 0.0000 | 6.98 | 0.000 | 4.04 | 0.0000 | 2.09 | ns |
| 18787 | Serpine1 | 2.93 | 0.0000 | 3.96 | 0.004 | 1.72 | 0.0043 | 2.37 | 0.0000 | 2.54 | 0.012 | 5.83 | 0.0005 | 23.88 | 0.000 |
| 100340 | Smpdl3b | 2.18 | 0.0000 | 3.25 | 0.047 | 2.06 | 0.0000 | 1.77 | 0.0021 | 3.37 | 0.001 | 7.53 | 0.0000 | 7.91 | 0.002 |
| 12702 | Socs3 | 5.20 | 0.0000 | 9.21 | 0.000 | 2.74 | 0.0000 | 2.82 | 0.0000 | 3.88 | 0.005 | 7.62 | 0.0000 | 5.06 | 0.003 |
| 20656 | Sod2 | 0.70 | 0.0000 | 0.40 | 0.006 | 0.82 | 0.0000 | 0.84 | 0.0061 | 0.50 | 0.021 | 0.67 | 0.0004 | **1.22** | ns |
| 20657 | Sod3 | 0.80 | 0.0250 | 0.34 | 0.000 | 0.94 | 0.1747 | 0.91 | ns | 0.45 | 0.009 | 0.59 | 0.0005 | 0.49 | ns |
| 20845 | Star | 0.63 | 0.0039 | 0.03 | 0.001 | 1.37 | 0.2922 | 0.87 | ns | 0.24 | ns | 0.29 | 0.3922 | 0.41 | ns |
| 21685 | Tef | 0.69 | 0.0004 | 0.65 | ns | 0.68 | 0.0013 | 0.59 | 0.0000 | 0.29 | 0.000 | 0.56 | 0.0000 | 0.97 | ns |
| 21803 | Tgfb1 | 1.96 | 0.0000 | 2.62 | 0.005 | 1.56 | 0.0000 | 1.42 | 0.0026 | 3.88 | 0.005 | 1.64 | 0.0004 | 3.28 | 0.006 |
| 21810 | Tgfbi | 3.91 | 0.0000 | 2.35 | 0.014 | 2.94 | 0.0000 | 3.02 | 0.0000 | 1.95 | 0.008 | 2.87 | 0.0000 | 1.16 | ns |
| 21857 | Timp1 | 2.79 | 0.0000 | 30.56 | 0.000 | 4.45 | 0.0000 | 3.54 | 0.0000 | 2272.40 | 0.011 | 4.99 | 0.0000 | 22.63 | 0.000 |
| 81897 | Tlr9 | 0.99 | ns. | **4.07** | 0.001 | 0.96 | 0.1246 | 0.95 | ns | **3.84** | 0.000 | 1.09 | 0.2954 | 1.46 | ns |
| 21926 | Tnf | 1.27 | 0.0216 | 10.32 | 0.000 | 1.01 | ns. | 1.01 | ns. | **3.37** | 0.001 | 1.19 | 0.0315 | 4.52 | 0.002 |
| 27279 | Tnfrsf12a | 2.32 | 0.0000 | 89.25 | 0.000 | 1.87 | 0.0000 | 2.03 | 0.0028 | 2.86 | ns | 5.47 | 0.0000 | 9.85 | 0.000 |
| 69583 | Tnfsf13=APRIL | nc | nc | 2.25 | 0.024 | nc | nc | nc | nc | 2.02 | 0.002 | nc | nc | 1.11 | ns |
| 24099 | Tnfsf13b=BAFF | 2.06 | 0.0000 | 4.81 | 0.000 | 1.36 | 0.0000 | 1.32 | 0.0000 | 3.11 | 0.001 | 1.56 | 0.0006 | 1.49 | ns |
| 22164 | Tnfsf4 | np | np | 7.59 | 0.000 | 0.96 | 0.1870 | 1.01 | ns. | not detected | ns | np | np | 2.15 | ns |
| 110253 | Triobp | 1.23 | 0.0138 | 1.09 | ns | 1.24 | 0.0000 | 1.22 | 0.0098 | **0.70** | ns | 1.40 | 0.0010 | 1.16 | ns |
| 22228 | Ucp2 | 1.85 | 0.0000 | 1.69 | 0.010 | 0.73 | 0.0418 | 0.92 | ns | 0.74 | ns | 2.01 | 0.0051 | 4.38 | 0.005 |
| 22329 | Vcam1 | 2.75 | 0.0000 | 6.54 | 0.000 | 2.72 | 0.0000 | 2.41 | 0.0000 | 4.44 | 0.001 | 10.71 | 0.0000 | 12.56 | 0.000 |
| 22339 | Vegfa | 0.71 | 0.0000 | 0.36 | 0.014 | 0.71 | 0.0000 | 0.74 | 0.0058 | 0.30 | 0.001 | 0.61 | 0.0004 | 0.79 | ns |
